# Infection with hepatitis C virus depends on TACSTD2, a regulator of claudin-1 and occludin highly downregulated in hepatocellular carcinoma

**DOI:** 10.1371/journal.ppat.1006916

**Published:** 2018-03-14

**Authors:** Vandana Sekhar, Teresa Pollicino, Giacomo Diaz, Ronald E. Engle, Farah Alayli, Marta Melis, Juraj Kabat, Ashley Tice, Anna Pomerenke, Nihal Altan-Bonnet, Fausto Zamboni, Paolo Lusso, Suzanne U. Emerson, Patrizia Farci

**Affiliations:** 1 Hepatic Pathogenesis Section, Laboratory of Infectious Diseases, National Institute of Allergy and Infectious Diseases, National Institutes of Health, Bethesda, Maryland, United States of America; 2 Division of Clinical and Molecular Hepatology, Department of Human Pathology, University of Messina, Messina, Italy; 3 Department of Biomedical Sciences, University of Cagliari, Cagliari, Italy; 4 Biological Imaging Facility/Research Technologies Branch, National Institute of Allergy and Infectious Disease, National Institutes of Health, Bethesda, Maryland, United States of America; 5 Laboratory of Host-Pathogen Dynamics, National Heart, Lung, and Blood Institute, National Institutes of Health, Bethesda, Maryland, United States of America; 6 Liver Transplantation Center, Brotzu Hospital, Cagliari, Italy; 7 Viral Pathogenesis Section, Laboratory of Immunoregulation, National Institute of Allergy and Infectious Diseases, National Institutes of Health, Bethesda, Maryland, United States of America; 8 Molecular Hepatitis Section, Laboratory of Infectious Diseases, National Institute of Allergy and Infectious Diseases, National Institutes of Health, Bethesda, Maryland, United States of America; The University of Chicago, UNITED STATES

## Abstract

Entry of hepatitis C virus (HCV) into hepatocytes is a complex process that involves numerous cellular factors, including the scavenger receptor class B type 1 (SR-B1), the tetraspanin CD81, and the tight junction (TJ) proteins claudin-1 (CLDN1) and occludin (OCLN). Despite expression of all known HCV-entry factors, *in vitro* models based on hepatoma cell lines do not fully reproduce the *in vivo* susceptibility of liver cells to primary HCV isolates, implying the existence of additional host factors which are critical for HCV entry and/or replication. Likewise, HCV replication is severely impaired within hepatocellular carcinoma (HCC) tissue *in vivo*, but the mechanisms responsible for this restriction are presently unknown. Here, we identify *tumor-associated calcium signal transducer 2 (TACSTD2)*, one of the most downregulated genes in primary HCC tissue, as a host factor that interacts with CLDN1 and OCLN and regulates their cellular localization. *TACSTD2* gene silencing disrupts the typical linear distribution of CLDN1 and OCLN along the cellular membrane in both hepatoma cells and primary human hepatocytes, recapitulating the pattern observed *in vivo* in primary HCC tissue. Mechanistic studies suggest that TACSTD2 is involved in the phosphorylation of CLDN1 and OCLN, which is required for their proper cellular localization. Silencing of *TACSTD2* dramatically inhibits HCV infection with a pan-genotype effect that occurs at the level of viral entry. Our study identifies TACSTD2 as a novel regulator of two major HCV-entry factors, CLDN1 and OCLN, which is strongly downregulated in malignant hepatocytes. These results provide new insights into the complex process of HCV entry into hepatocytes and may assist in the development of more efficient cellular systems for HCV propagation *in vitro*.

## Author summary

Infection with hepatitis C virus (HCV) is associated with a high risk of developing hepatocellular carcinoma (HCC). However, it is unknown whether HCV promotes HCC indirectly, through chronic inflammation, fibrosis and liver regeneration, or directly, in a manner similar to other oncogenic viruses. We recently demonstrated that HCV replication within HCC tissue is severely hampered, and the present study identifies a potential mechanism for this restriction. We found that HCV RNA suppression in the tumor correlates with disruption of the typical linear distribution of two major HCV-entry cofactors, claudin-1 (CLDN1) and occludin (OCLN), which mediate viral entry into hepatocytes. This abnormal pattern was associated with downregulation of *tumor-associated calcium signal transducer 2 (TACSTD2)*, an essential gene for maintaining the proper cellular localization of CLDN1 and OCLN. Thus, this study identifies TACSTD2 as a novel host factor that regulates the cellular localization of two major HCV-entry cofactors. A major highlight of our study is that our *in vitro* observations recapitulate the *in vivo* findings in patients with HCC, corroborating their biological relevance. These results shed new light on the complex process of HCV entry and may have relevance for the pathogenesis, treatment and prevention of HCV infection.

## Introduction

Infection with hepatitis C virus (HCV) is a major public health problem worldwide because it is a primary cause of chronic hepatitis, cirrhosis and hepatocellular carcinoma (HCC) [1]. Although there have been remarkable advances in our knowledge of HCV and its related disease manifestations, several challenges have hampered pathogenesis studies. The lack of small animal models and efficient *in vitro* systems are certainly among the most daunting obstacles [2]. The current HCV cell culture systems based on hepatoma cell lines have major limitations, including their inability to recapitulate the features of functional liver tissue and to support the replication of primary HCV isolates. Likewise, infection of primary hepatocytes cultured *ex vivo* has been challenging. Thus, the development of a fully permissive HCV cell culture system continues to be a formidable task. Despite these hurdles, a large number of studies have started to define the HCV life cycle, including viral entry, which is a complex and finely orchestrated multi-step process [3]. Several host proteins that permit HCV entry have been identified, including the two tight junction (TJ) proteins claudin-1 (CLDN1) [4] and occludin (OCLN) [5], the tetraspanin CD81 [6], and the scavenger receptor class B type1 (SR-B1) [7]. Moreover, additional cellular cofactors, such as the receptor tyrosine kinases epithelial growth factor receptor (EGFR) and ephrin receptor A2 (EphA2) [8], the low density lipoprotein receptor (LDLR) [9], the cholesterol transporter Niemann-Pick C1-like 1 (NPC1L1) [10], the iron-uptake protein transferrin receptor 1 (TfR1) [11], serum response factor binding protein 1 (SRFBP1) [12] and E-cadherin [13] have been shown to regulate the complex multistep HCV-entry process.

Despite expression of all known HCV-entry factors, *in vitro* models based on hepatoma cell lines do not fully reproduce the *in vivo* susceptibility of liver cells to primary HCV isolates, implying the existence of additional host factors which are critical for HCV entry and/or replication [2, 14]. Likewise, we and others have reported that HCV replication is severely impaired within HCC tissue *in vivo* [15–18], but the mechanisms responsible for this restriction are presently unknown. In a recent comprehensive study examining HCV RNA levels in different liver compartments of patients with HCV-associated HCC, including the tumor, the perilesional area and the surrounding nontumorous tissue, we documented a sharp, significant drop of HCV RNA levels in primary HCC tissues within millimeters of the perilesional area [18]. This dramatic reduction in HCV RNA was confined to malignant hepatocytes and was not related to changes in miR122 expression [18], an essential host cofactor for HCV replication [19, 20], suggesting that malignant hepatocytes express factors or, more likely, have lost expression of factors that regulate HCV infection and/or replication. To elucidate the nature of such factors, we performed transcriptomics analysis in multiple liver specimens spanning the entire liver of patients with HCV-associated HCC. Among the genes differentially expressed between the tumor and nontumorous areas that showed the strongest correlation with HCV RNA levels, we focused on the *tumor-associated calcium signal transducer 2 (TACSTD2*), a gene previously reported to be important for the proper cellular localization of CLDN1 and OCLN in human corneal epithelial cells [21]. The objective of the current study was to investigate if TACSTD2 plays a role in modulating HCV entry and/or replication. The results of this study identify TACSTD2 as a novel host factor for HCV infection that acts via regulation of two critical HCV-entry cofactors.

## Results

### Restricted HCV replication in malignant hepatocytes of patients with HCC correlates with disrupted localization of the HCV-entry cofactors CLDN1 and OCLN

**Fig 1:**
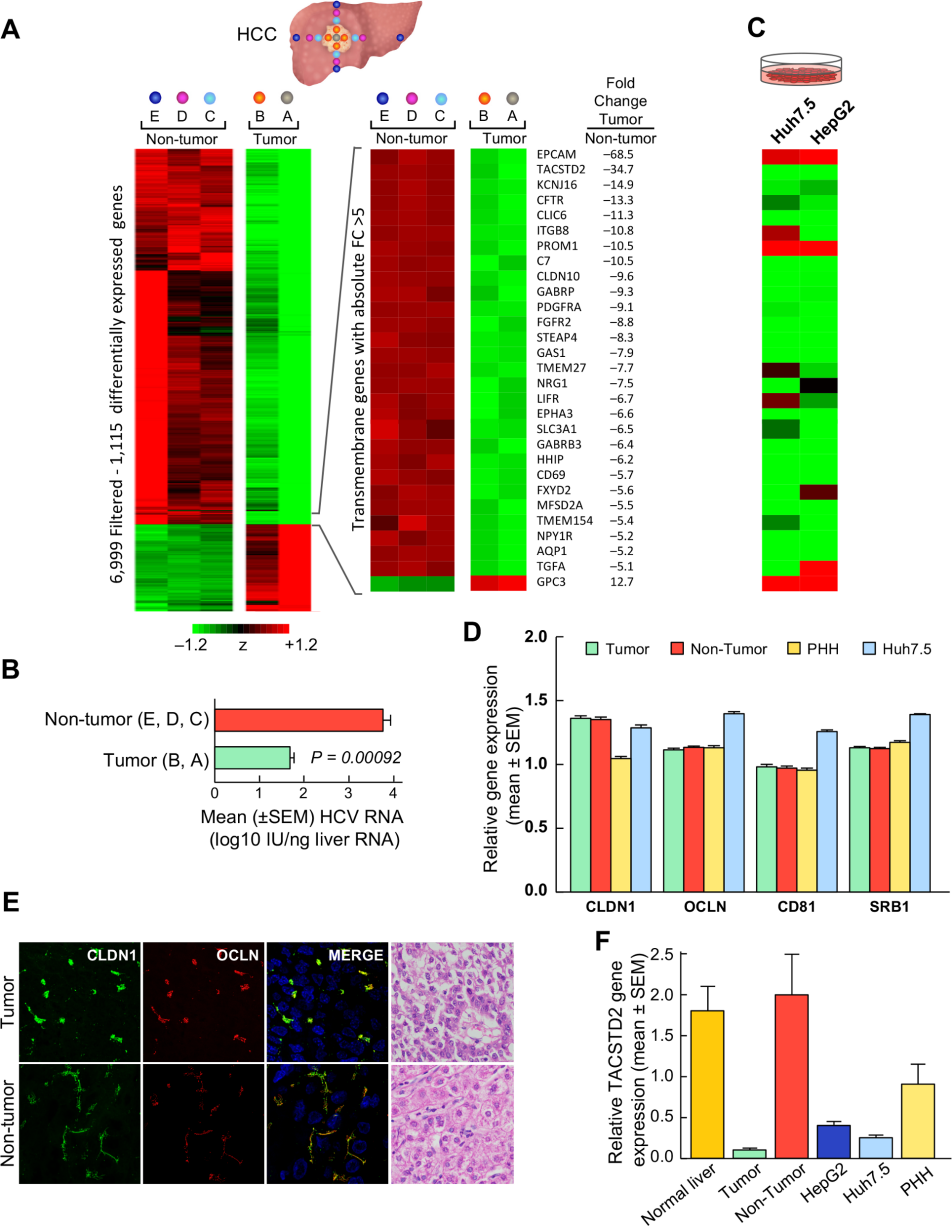
Gene expression profiling and HCV RNA in tumor and nontumorous liver tissue of patients with HCV-associated HCC. (A) Liver samples were obtained from five different liver areas: the center of the tumor (A, 8 samples), the periphery of the tumor (B, 29 samples), the perilesional nontumorous area outside the tumor margin (C, 21 samples), the area at a distance of 2–3 cm from the margin of the tumor (D, 22 samples), and the most distant nontumorous area, on the edge of the liver (E, 23 samples). Data from the center of the tumor area (A) represent individual samples, whereas data from each of the other liver areas (B to E) represent the average of multiple specimens obtained in the 4 directions. Differentially expressed genes (n = 1,115) were identified by comparing A (tumor) and E (non-tumor) areas (FDR<1%). The heatmap shows the standardized expression of the 1,115 genes in all five liver areas. Genes whose expression was above the mean are shown in shades of red, and genes whose expression was below the mean are shown in shades of green. The full range of colors, from green to red, is ± 1.2 SD. The heat map and fold changes of the most upregulated transmembranes genes are also shown. (B) HCV RNA shows a significant drop in the tumorous areas (A, B), as compared to the surrounding nontumorous areas (C, D, E). The bars represent the mean ± SEM. The data presented in this figure on the levels of HCV RNA in the tumor and surrounding nontumorous tissue from these patients were previously reported [18]. (C) Heat map representing the most upregulated transmembrane genes in hepatoma cell lines: Huh7.5 and HepG2. (D) Quantitative RT-PCR of the 4 HCV-entry cofactors CLDN1, CD81, OCLN, and SR-B1 in tumor, nontumorous tissue, Huh7.5 cells, and primary human hepatocytes (PHH). Data represent the log2-transformed ratio of each co-receptor to GAPDH. No differences were found between tumor and non-tumor in any of the 4 HCV-entry cofactors. Comparable levels were also observed for PHH except for a lower expression of CLDN1, whereas Huh7.5 showed a higher expression of 3 entry cofactors (CD81, OCLN and SR-B1). (E) Visualization of CLDN1 (green surface rendering) and OCLN (red surface rendering) in tumor and non-tumor specimens in a representative patient who exhibited a 3 log drop in HCV RNA levels within the tumor by immunofluorescence staining. Two patterns of CLDN1 and OCLN cellular distribution were documented: the tumor shows a clumpy distribution of CLDN1 and OCLN whereas the matching nontumorous tissue shows a regular linear distribution along the cellular membrane. DAPI is represented in blue. Overlay images show all channels in 3D volume rendering. The H&E images show the tumor and nontumorous liver specimens of the same patient (40X). (F) Quantitative RT-PCR of TACSTD2. Mean relative quantities are normalized to the non-tumor tissue, expressed as 2^-ΔΔCT^, where ΔΔC_T_ is the average difference between the target tissue ΔC_T_ and the non-tumor tissue ΔC_T_.

To elucidate the role of host factors in restricting viral replication in the tumor tissue of patients with HCV-associated HCC, we performed gene expression profiling on a series of liver specimens that we had previously used for measuring the levels of viral replication [18]. Up to 17 liver specimens, collected at the time of liver transplantation from each of 8 well-characterized patients with HCV-associated HCC, were analyzed, including 5 samples from the tumor and 12 from the surrounding nontumorous tissue. A sharp change in gene expression was observed between the tumor periphery and the immediate perilesional area, whereas no significant changes were found between different nontumorous areas (Fig 1A). This change in gene expression was paralleled by a significant drop of HCV replication within the tumor (Fig 1B). To identify the genes responsible for the marked difference between tumor and nontumorous tissue, the center of the tumor (area A) and the most distant nontumorous area (area E) were compared by a multivariate permutation F-test (FDR<5%). The analysis identified 1,115 differentially expressed genes (S1 Table) with a large preponderance (76%) of downregulated genes within the tumor. The vast majority of these genes are involved in cell-mediated immune responses, including but not limited to T-cell receptor signaling, CD28 and iCOS-iCOSL signaling in T-helper cells, NFAT regulation of immune responses, CTLA4 signaling in CTL, cytokine/chemokine-related signaling, and STAT3 pathway (S1A and S1B Fig). Other top-scored pathways included hepatic fibrosis/HSC activation, ErB signaling, IGF1 signaling and GH signaling. All categories showed a striking majority (on average 90%) of downregulated genes. A heat map of immune genes showed an abrupt separation between tumor and nontumorous tissue with almost all genes downregulated within the tumor (S2 Fig), Another feature of the malignant hepatocytes was downregulation of transmembrane genes, most of which were also downregulated in hepatoma cell lines (Fig 1A–1C). However, none of the four major HCV-entry cofactors, SR-B1, CD81, CLDN1 and OCLN, was differentially expressed between the tumor and the nontumorous tissue, as also confirmed by real-time PCR analysis (Fig 1D), thus ruling out a role of downregulation of these major entry cofactors in the restriction of HCV replication within the tumor. Other host factors more recently described to be involved in regulating cellular proteins important for HCV entry, including EGFR, LDLR, NPC1L1, TfR1, SRFBP1, as well as epithelial-to-mesenchymal transition (EMT) markers such as E-cadherin and vimentin were analyzed, but none of them were differentially expressed between tumor and nontumorous tissue (S3 Fig).

Next, we investigated the pattern of expression of the 4 major HCV-entry cofactors in primary liver tissue from patients with HCV-associated HCC by confocal microscopy. Strikingly, we found that the TJ proteins CLDN1 and OCLN showed an abnormal distribution within the tumor tissue, appearing clumpy and aggregated compared to the typical linear pattern lining the cellular membranes observed in the surrounding nontumorous tissue (Fig 1E). These differences were specific for CLDN1 and OCLN, as both SR-B1 and CD81 displayed typical linear distributions both within and outside the tumor (S4 Fig). We also investigated the expression of SEC14L2, a recently identified intracellular factor that enables RNA replication of diverse HCV genotypes in hepatoma cell lines [22]. *SEC14L2* gene expression showed no significant difference between tumor and nontumorous tissues (*P* = 0.16), and no correlation with the levels of HCV replication (*r* = 0.275, *P* = 0.442) (S5A Fig). Likewise, other genes reported to be important for HCV replication *in vitro*, such as cyclophilin A, phosphatidylinositol 4-kinase (PI4KIII)-α, and DEAD-box helicase 3, X-linked (DDX3X) [23], were not differentially expressed between the tumor and the nontumorous tissue.

### TACSTD2 is among the most downregulated genes in HCV-associated HCC

Among the transmembrane genes differentially expressed between the tumor and the surrounding nontumorous areas, we focused on *tumor-associated calcium signal transducer 2* (*TACSTD2*) [24], also known as *trophoblast antigen 2* (*Trop2*), because it was the second most downregulated gene within the tumor (Fig 1A and S1 Table) and showed a very strong correlation with HCV RNA levels (*r* = 0.71, *P*<0.00001) (S5B Fig). The decreased expression of *TACSTD2* within the tumor compared to the surrounding nontumorous areas was confirmed by real-time PCR (Fig 1F). *TACSTD2* is an intronless gene encoding a type-I cell-surface glycoprotein that functions as an intracellular calcium signal transducer [24] and is dysregulated in many epithelial cancers [25–27]. TACSTD2 has previously been reported to impact the cellular localization of CLDN1 and OCLN in human corneal epithelial cells [21]. Mutations in the *TACSTD2* gene result in gelatinous drop-like corneal dystrophy [28], an autosomal recessive disease that causes blindness due to amyloid deposition arising from the loss of epithelial barrier function [29]. These data, along with the significant downregulation of *TACSTD2* and the abnormal distribution of CLDN1 and OCLN in primary liver tissue from HCV-associated HCC patients, prompted us to further investigate the role of TACSTD2 in HCV infection. Among all known host factors reported to date that regulate cellular proteins important for HCV entry, TACSTD2 was the only one to be differentially expressed between tumor and surrounding nontumorous tissue (S3 Fig).

### TACSTD2 interacts with the HCV-entry cofactors CLDN1 and OCLN in Huh7.5 cells

**Fig 2:**
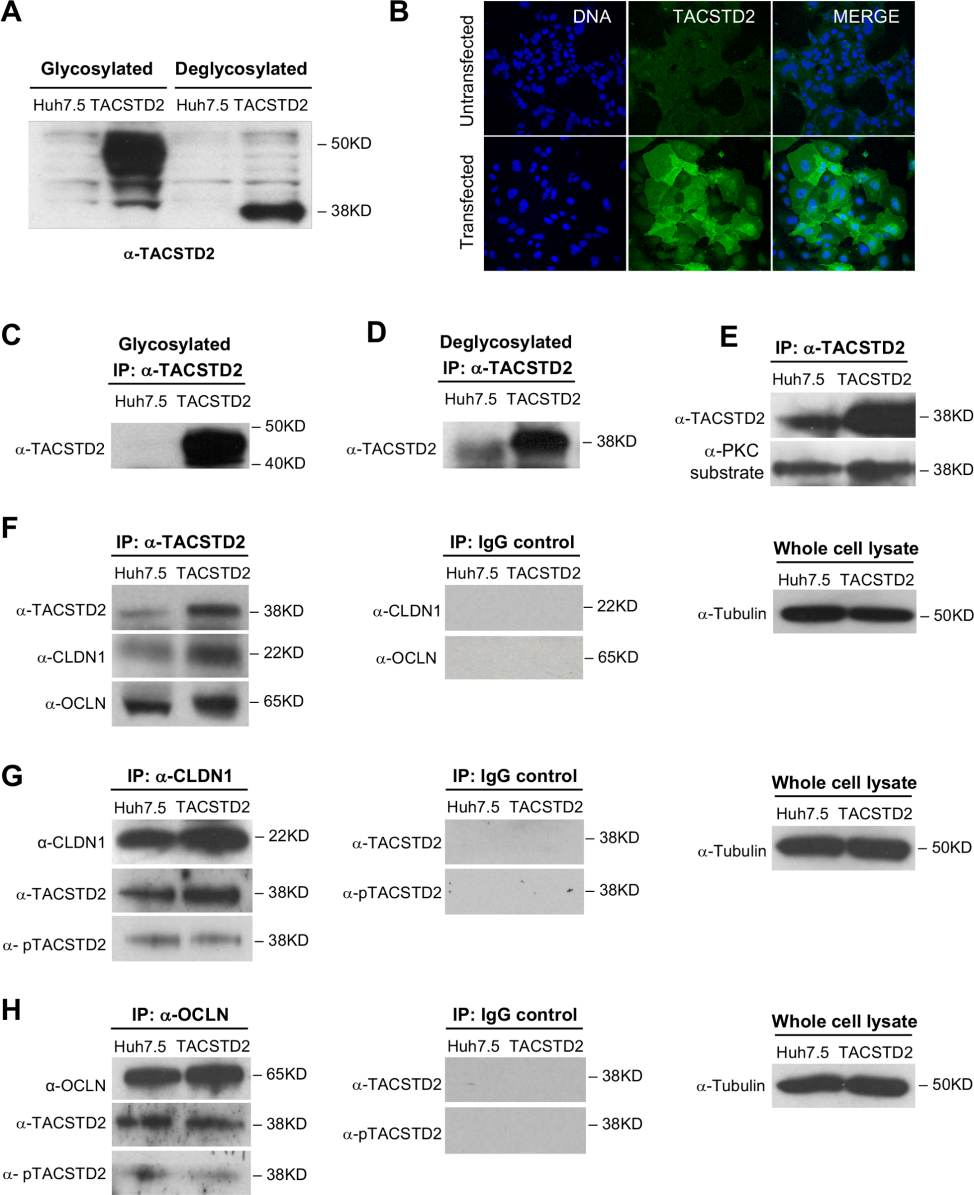
TACSTD2 expression and interaction with HCV-entry cofactors in hepatoma cells. (A) Western blot analysis of TACSTD2 expression in parental Huh7.5 cells and TACSTD2-overexpressing Huh7.5 cells (TACSTD2). TACSTD2 expression was below the detection limit of Western blot in parental cells (lane 1) but was visible in TACSTD2-overexpressing cells as a broad band due to its poly-glycoslyated state (lane 2). Enzymatic deglycosylation resulted in the protein migration as a single band of 37KD, as seen in lane 4. (B) Immunofluorescence detection of TACSTD2 (green) in parental and TACSTD2-overexpressing Huh7.5 cells. No TACSTD2 could be visualized in parental Huh7.5 (top panel), whereas in stably transfected cells, TACSTD2 was localized both in the cytoplasm and along the cellular membrane (lower panel). (C) Detection of TACSTD2 in parental and TACSTD2-overexpressing Huh7.5 cells by immunoprecipitation with an anti-TACSTD2 antibody without deglycosylation. (D) Detection of TACSTD2 in parental and TACSTD2-overexpressing Huh7.5 cells by immunoprecipitation with an anti-TACSTD2 antibody followed by deglycosylation. (E) Phosphorylation of TACSTD2 in parental and TACSTD2-overexpressing Huh7.5 cells. Cell lysates were immunoprecipitated with anti-TACSTD2 antibody and then deglycosylated. Phosphorylated TACSTD2 was detected using an anti-PKC substrate-specific antibody; total TACSTD2 was detected using biotinylated anti-TACSTD2 antibody. Phosphorylated TACSTD2 was detected in both parental and TACSTD2-overexpressing Huh7.5 cells. (F) Co-immunoprecipitation of CLDN1 and OCLN by anti-TACSTD2 antibody in parental and TACSTD2-overexpressing Huh7.5 cells (lanes 1, 2). Lysates prepared from both parental and TACSTD2-overexpressing Huh7.5 cells were immunoprecipitated with anti-TACSTD2 antibody or control IgG, deglycosylated, and probed with antibodies specific for TACSTD2, CLDN1, and OCLN. (G) Reciprocal co-immunoprecipitation of TACSTD2 by anti-CLDN1 antibody in parental and TACSTD2-overexpressing Huh7.5 cells (lanes 1, 2). Cell lysates were immunoprecipitated with anti-CLDN1 antibody or control IgG. After deglycosylation, total TACSTD2 was detected using biotinylated anti-TACSTD2 antibody, phosphorylated TACSTD2 was detected using an anti-PKC substrate-specific antibody, and CLDN1 was detected using an anti-CLDN1 specific antibody. (H) Reciprocal co-immunoprecipitation of TACSTD2 by anti-OCLN antibody in parental and TACSTD2-overexpressing Huh7.5 cells (lanes 1, 2). Cell lysates were immunoprecipitated with anti-OCLN antibody or control IgG, deglycosylated, and probed with biotinylated anti-TACSTD2 antibody, anti-PKC substrate-specific antibody to detect phosphorylated TACSTD2, and anti-OCLN specific antibody.

Comparative analysis by RT-PCR revealed that primary tumor tissue from patients with HCV-associated HCC expressed lower levels of TACSTD2 mRNA compared to the hepatoma cell lines Huh7.5 and HepG2, whereas the surrounding nontumorous tissue, normal livers, and primary human hepatocytes expressed the highest levels (Fig 1F). Although the levels of TACSTD2 expression in Huh7.5 cells were higher than in primary tumor tissue, they were below the threshold of detection by conventional Western blot (Fig 2A) and immunofluorescence (Fig 2B). Thus, we generated a Huh7.5 cell line stably overexpressing *TACSTD2*, in which the protein could be detected by both methods. In these cells, by Western blot, the protein migrated as a broad band due to heterogeneous glycosylation, while it appeared as a single band upon deglycosylation (Fig 2A); by confocal microscopy, the protein was visualized along the cellular membrane, as well as in the cytoplasm, most likely as a result of abnormal accumulation of the overexpressed protein (Fig 2B). By immunoprecipitation without deglycosylation (Fig 2C), a strong TACSTD2 band was visible only in TACSTD2 overexpressing cells, but not in parental Huh7.5 cells where the band became detectable only by immunoprecipitation followed by deglycosylation (Fig 2D).

TACSTD2 has been reported to be phosphorylated by protein kinase C (PKC), an important cellular kinase involved in multiple biological processes [27]. Thus, we studied the phosphorylation status of TACSTD2 in hepatoma cells by immunoprecipitation with anti-TACSTD2 antibody followed by deglycosylation and Western blot analysis using a PKC substrate-specific antibody. Phosphorylated TACSTD2 bands were detected in both parental and TACSTD2-overexpressing Huh7.5 cells (Fig 2E), indicating that in hepatoma cells the protein is functionally competent, as previously reported [27].

Since TACSTD2 was reported to interact with the HCV-entry cofactor CLDN1 in human corneal epithelial cells [21], we examined the interaction between TACSTD2 and CLDN1 in hepatoma cells by co-immunoprecipitation. In both parental and TACSTD2-overexpressing Huh7.5 cells, CLDN1 was co-immunoprecipitated by an anti-TACSTD2 antibody (Fig 2F), indicating that the two proteins interact, either directly or indirectly, in hepatoma cells. Reciprocal co-immunoprecipitation with an anti-CLDN1 antibody confirmed the interaction between TACSTD2 and CLDN1 in both cell lines (Fig 2G). To further examine the molecular interaction of TACSTD2 with CLDN1, we assessed the role played by TACSTD2 phosphorylation in this interaction. Western blot analysis documented the presence of phosphorylated TACSTD2 in the complex immunoprecipitated with anti-CLDN1 antibody in both parental and TACSTD2-overexpressing Huh7.5 cells, indicating that phosphorylated TACSTD2 interacts with CLDN1 (Fig 2G). To elucidate the spatial interactions between TACSTD2 and CLDN1, we examined their localization in hepatoma cells by confocal microscopy. In parental Huh7.5 cells, although TACSTD2 could not be visualized, CLDN1 showed the typical linear pattern along the cellular membrane (S6 Fig). Interestingly, in *TACSTD2*-overexpressing cells CLDN1 co-localized with TACSTD2 along the cellular membrane, but not in the cytoplasm (S6 Fig).

Next, we examined whether TACSTD2 also interacts with the other TJ protein that functions as HCV-entry cofactor, OCLN. When lysates from parental and TACSTD2-overexpressing Huh7.5 cells were immunoprecipitated with anti-TACSTD2 antibody, an OCLN-specific band was detected in both cell lines (Fig 2F), indicating that TACSTD2 interacts with OCLN. Reciprocal co-immunoprecipitation with an anti-OCLN antibody followed by Western blot analysis confirmed the interaction between the two proteins (Fig 2H). Moreover, we documented the presence of phosphorylated TACSTD2 in the complex immunoprecipitated with anti-OCLN antibody in both parental and TACSTD2-overexpressing Huh7.5 cells (Fig 2H), indicating that the phosphorylated form of TACSTD2 interacts not only with CLDN1 but also with OCLN. As a negative control, we tested the retinoblastoma protein, which did not co-immunoprecipitated with TACSTD2 (S7 Fig).

### TACSTD2 affects the subcellular localization of CLDN1 and OCLN

**Fig 3:**
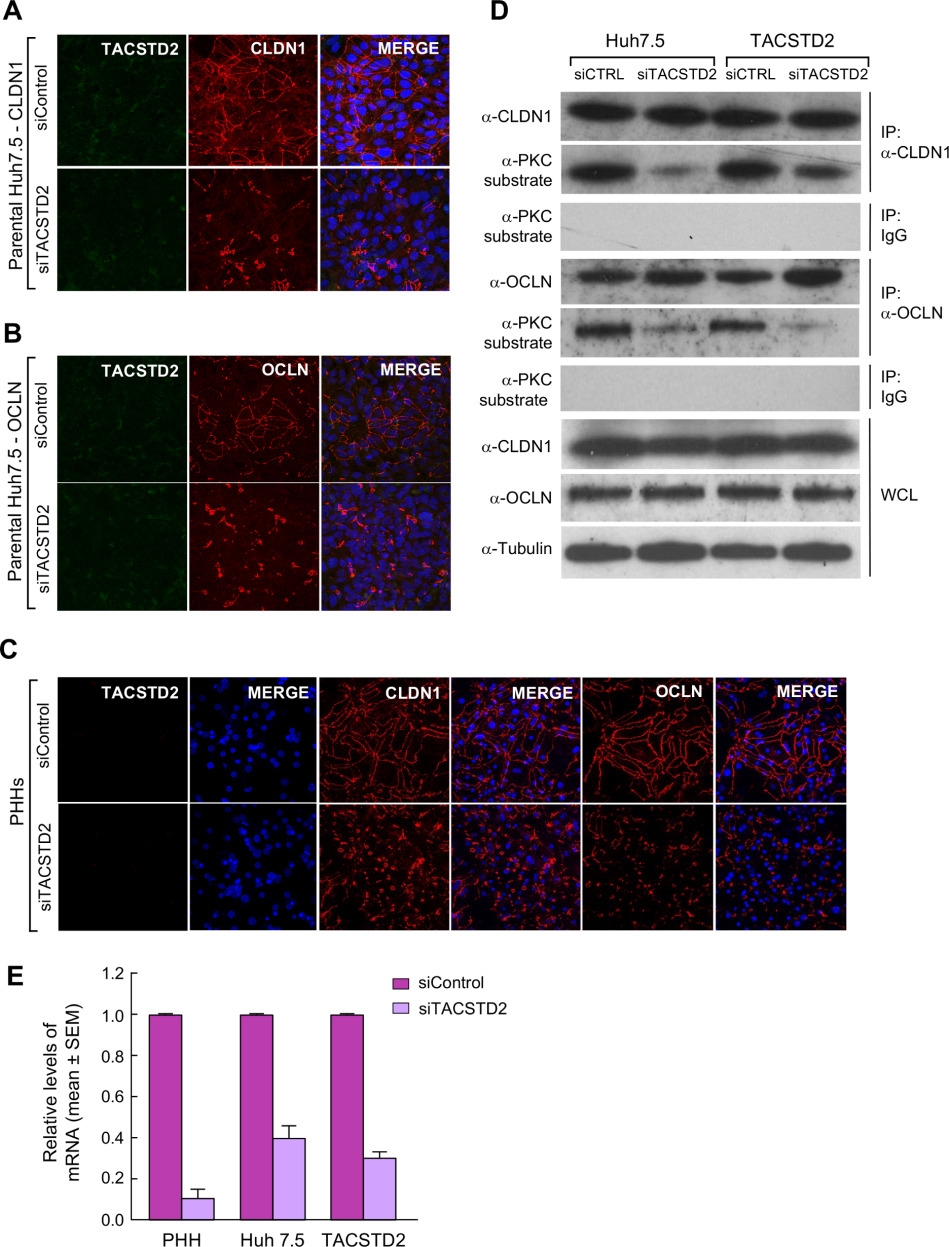
*TACSTD2* gene silencing disrupts CLDN1 and OCLN cellular localization in hepatoma cells and primary human hepatocytes. (A) Visualization of TACSTD2 (green) and CLDN1 (red) in parental Huh7.5 cells transfected with siTACSTD2 or an irrelevant siRNA (siControl). CLDN1 appears speckled and fragmented in siTACSTD2-treated cells but maintains its regular linear pattern along the cellular membrane in siControl-treated cells. (B) Visualization of TACSTD2 (green) and OCLN (red) in parental Huh7.5 cells transfected with siTACSTD2 or siControl. OCLN appears speckled and fragmented in siTACSTD2-treated cells but maintains its regular linear pattern along the cellular membrane in siControl-treated cells. (C) Visualization of CLDN1 and OCLN in primary human hepatocytes transfected with siTACSTD2 or siControl. Both CLDN1 and OCLN appear speckled and fragmented in siTACSTD2-treated cells but maintain their regular linear distribution along the cellular membrane in siControl-treated cells. (D) *TACSTD2* gene silencing results in the reduction of phosphorylation levels of CLDN1 and OCLN in both parental and TACSTD2-overexpressing Huh7.5 cells. Cell lysates from both parental and TACSTD2-overexpressing Huh7.5 cells were immunoprecipitated with anti-CLDN1 and anti-OCLN antibodies or control IgG, at 72h after transfection with either siControl or siTACSTD2. Phosphorylated CLDN1 and OCLN were detected using an anti-PKC substrate-specific antibody; total CLDN1 and total OCLN were detected using anti-CLDN1 and anti-OCLN specific antibodies, respectively. Whole cellular lysates were simultaneously subjected to immunoblotting using anti-CLDN1 and anti-OCLN antibodies. (E) RT-PCR quantification of TACSTD2 in siControl- and siTACSTD2-transfected cells, expressed as 2^-ΔΔCT^, where ΔΔC_T_ is the average difference between the siTACSTD2 ΔC_T_ and siControl ΔC_T_.

To investigate the role of TACSTD2 in regulating CLDN1 and OCLN distribution in hepatoma cells, we performed *TACSTD2* gene silencing using a pool of 4 siRNA (siTACSTD2) and examined the distribution of the two HCV-entry cofactors by confocal microscopy. While cells transfected with control siRNA (siControl) maintained the typical linear pattern of CLDN1 distribution along the cellular membrane, *TACSTD2* silencing induced disruption of CLDN1 cellular localization, yielding a fragmented and clumpy pattern in both parental Huh7.5 cells (Fig 3A) and TACSTD2-overexpressing cells (S8A Fig). Likewise, OCLN lost its typical linear pattern and appeared clumpy and aggregated in both parental Huh7.5 cells (Fig 3B) and TACSTD2-overexpressing cells (S8B Fig) treated with siTACSTD2. Neither overexpression nor silencing of *TACSTD2* had significant effects on the survival and proliferation of Huh7.5 cells (S8C and S8D Fig). Because the level of endogenous TACSTD2 expression in parental Huh 7.5 cells was very low, as shown by real-time PCR (Fig 1F), TACSTD2 silencing in parental Huh 7.5 cells was assessed by a combination of IP-WB, immunofluorescence and real-time PCR (S9 Fig). Although the results of the three analysis were generally concordant, the most sensitive and consistent method to assess TACSTD2 silencing was the disruption of CLDN1, OCLN, and zonula occludens-1 (ZO-1) by confocal microscopy (S9 Fig). In contrast to parental Huh7.5 cells, *TACSTD2* gene silencing in overexpressed Huh7.5 cells was also clearly visible by immunofluorescence (S8A and S8B Fig). To confirm the biological relevance of CLDN1 and OCLN disruption upon *TACSTD2* silencing, we studied the same effect in primary human hepatocytes, where TACSTD2 was not detectable by confocal microscopy (Fig 3C). While in siControl-transfected primary hepatocytes, both CLDN1 and OCLN showed the typical linear localization along the cellular membrane, their pattern was markedly altered in TACSTD2-silenced hepatocytes, with both CLDN1 and OCLN appearing clumpy and aggregated as observed in Huh7.5 cells (Fig 3C). Strikingly, the abnormal localization of CLDN1 and OCLN that we documented following *TACSTD2* silencing both in hepatoma cell lines and in primary human hepatocytes was analogous to that observed in tumor tissue of patients with HCV-associated HCC (Fig 1E).

Next, we examined whether *TACSTD2* gene silencing could impact the cellular localization of other TJ or adherens junction (AJ) proteins. We found that the TJ proteins ZO-1 and junctional adhesion molecule-A (JAM-A) showed a disrupted cellular localization in Huh7.5 cells upon TACSTD2 gene silencing, compared with siControl-transfected cells (S10A and S10B Fig), indicating that TACSTD2 is critical for the proper cellular localization not only of CLDN1 and OCLN but also of other TJ proteins such as ZO-1 and JAM-A. Then, we investigated the effects of *TACSTD2* gene silencing on the expression and localization of E-cadherin. Following TACSTD2 silencing in Huh7.5 cells, we found no significant alteration in the expression of E-cadherin by WB (S11A Fig), as well as no change in E-cadherin localization by confocal microscopy, as compared with siControl-transfected cells (S11B Fig). Moreover, silencing of TACSTD2 was not associated with signs of epithelial-to-mesenchymal transition (EMT), i.e., E-cadherin downregulation associated with vimentin upregulation, as Li et al. recently reported upon HCV infection [13]. By real-time PCR, we observed an opposite trend, with moderate increase of E-cadherin and decrease of vimentin (S12 Fig). Furthermore, in primary HCC tumor tissue we found no alteration in the expression of EMT markers compared to the surrounding nontumorous tissue, in spite of the significant reduction of HCV RNA and downregulation of TACSTD2 (S3 Fig).

### TACSTD2 regulates the phosphorylation and intracellular localization of CLDN1 and OCLN at the post-translational level

To gain further insights into the mechanistic interaction of TACSTD2 with CLDN1 and OCLN, we investigated whether TACSTD2 regulates these proteins at the transcriptional or post-transcriptional level. No significant differences were observed in CLDN1 and OCLN mRNA and protein levels between siControl- and siTACSTD2-transfected hepatocytes (S13A and S13B Fig). Since it has been reported that phosphorylation of CLDN1 and OCLN plays a crucial role in maintaining the proper cellular localization of these two proteins [30, 31], we hypothesized that TACSTD2, which is an intracellular calcium signal transducer that activates the calcium-dependent kinase PKC [27, 32], may participate in the regulation of CLDN1 and OCLN phosphorylation. Immunoprecipitation with anti-CLDN1 and anti-OCLN antibodies followed by Western blot analysis using a PKC substrate-specific antibody showed a significant reduction of CLDN1 and OCLN phosphorylation in *TACSTD2*-silenced cells compared with siControl-transfected cells (Fig 3D and 3E). These data suggest that TACSTD2 plays an important role in the PKC-mediated phosphorylation of CLDN1 and OCLN, thereby affecting their cellular localization.

### *TACSTD2* gene silencing inhibits HCV infection with a pan-genotype effect

**Fig 4:**
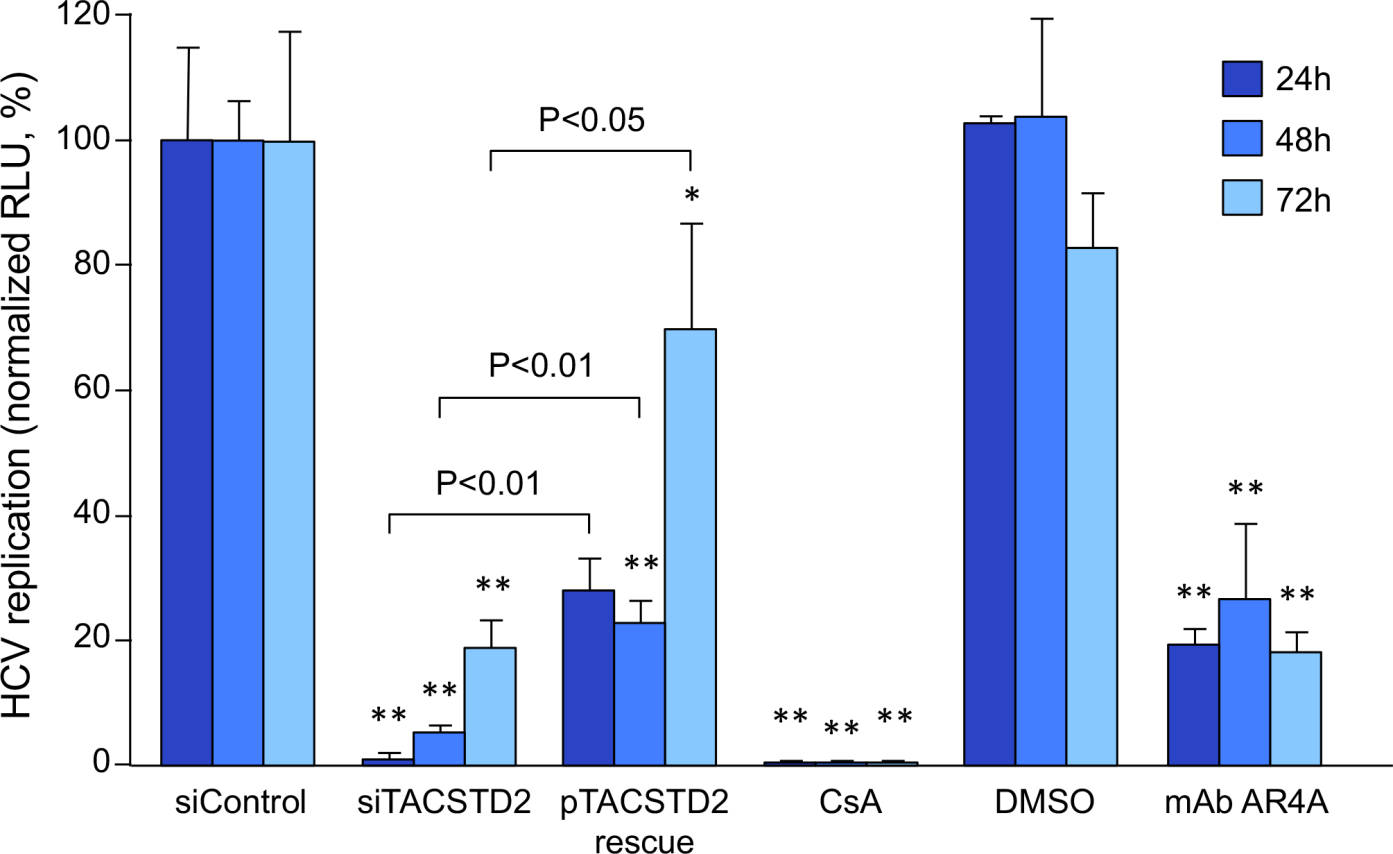
*TACSTD2* gene silencing results in inhibition of HCV infection. The replication-competent virus HCV-Luc was used to infect parental Huh7.5 cells transfected with either siControl or siTACSTD2, or cotransfected with siTACSTD2 plus an expression vector encoding siRNA-resistant TACSTD2 (pTACSTD2). The AR4A neutralizing anti-HCV envelope monoclonal antibody (50 μg/mL), as well as the inhibitor of HCV replication, cyclosporin A (CsA; 10 μM) and DMSO (solvent of CsA) served as controls. At 24h, 48h, and 72h post-infection the cells were lysed, and viral replication was assessed by measurement of luciferase reporter gene activity. Values are normalized to relative light unit (RLU) from siControl-treated cells (raw RLU values [mean ± SEM] for siC: 24h, 3390 ± 264; 48h, 74397 ± 2744; 72h, 16107 ± 1603) and represent the mean ± SEM (n = 3). In all panels, asterisks indicate statistically significant differences versus siControl, by t-test (*P<0.05; **P<0.01).

**Fig 5:**
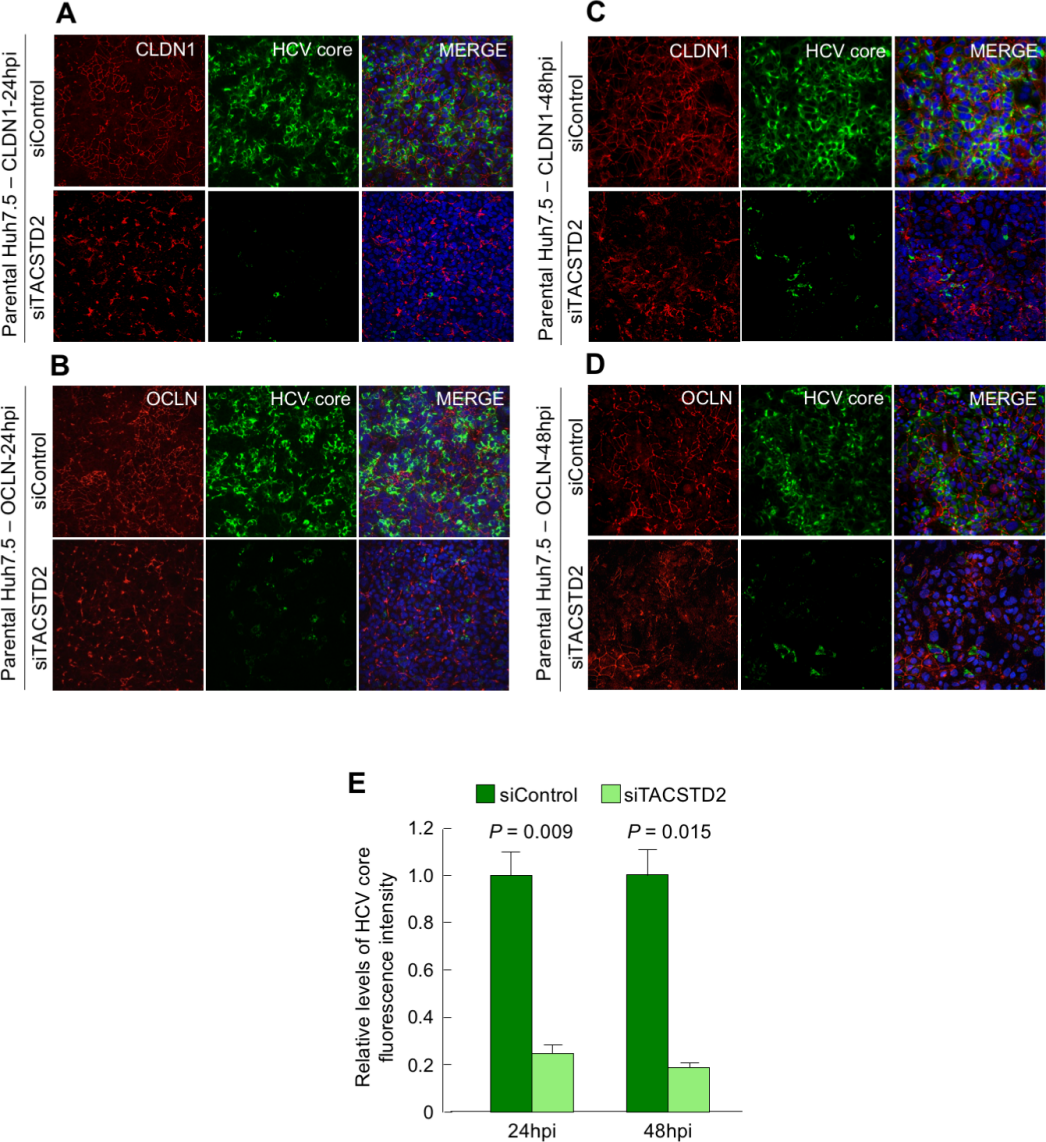
Effect of TACSTD2 gene silencing on HCV infection in parental Huh7.5 cells. (A-D) Parental Huh7.5 cells were transfected with either siControl or siTACSTD2 for 48h before infection with HCV. Cells were fixed with methanol either at 24h (A, B) or 48h post-infection (C, D) and visualized by immunofluorescence for CLDN1 (red)/HCV core (green) or OCLN (red)/HCV core (green) staining. (E) Relative levels of HCV infection at 24h and at 48h post-infection as shown by the semiquantitative representation of HCV core positive cells in parental Huh7.5 cells. Data represent the normalized mean ± SEM of the HCV core fluorescence intensity of triplicate experiments. A significant difference in HCV core fluorescence intensity between siControl- and siTACSTD2-treated cells was detected by paired t-test both at 24h and at 48h post-infection.

Having demonstrated that *TACSTD2* gene silencing disrupts the cellular localization of two critical HCV-entry cofactors, CLDN1 and OCLN, we investigated the role of TACSTD2 in HCV infection. Parental Huh7.5 cells were transfected with siTACSTD2 or siControl and then infected with the replication-competent virus HCV-Luc [J6/JFH(p7-Rluc2A)], which expresses a reporter gene (Renilla luciferase) [33, 34]. To exclude non-specific off-target effects, the cells were also co-transfected with siTACSTD2 plus an expression vector encoding siRNA-resistant *TACSTD2*. As additional controls, we used a broadly neutralizing anti-HCV envelope monoclonal antibody (mAb), AR4A [35], as well as the inhibitor of HCV replication, cyclosporin A [34, 36]. *TACSTD2* silencing specifically inhibited HCV-Luc infection at all the time points examined, with even greater efficiency than mAb AR4A (Fig 4). Importantly, the effect of endogenous *TACSTD2* silencing on HCV infection was rescued by expression of a siRNA-resistant exogenous *TACSTD2* (Fig 4). The role of TACSTD2 in HCV infection was confirmed by immunofluorescence using a replication-competent variant of the HCV strain JFH1 (JFH1-AM2) selected *in vitro* for its efficiency of replication in hepatoma cells [37]. A marked reduction in HCV-core antigen expression was observed in siTACSTD2-treated parental Huh7.5 cells at both 24h and 48h post-infection (Fig 5A–5D), as corroborated by semi-quantitative fluorescence analysis (*P* = 0.009 and P = 0.015, respectively; Fig 5E). Inhibition of HCV infection was associated with disruption of the proper cellular localization of both CLDN1 (Fig 5A–5C) and OCLN (Fig 5B–5D).

**Fig 6:**
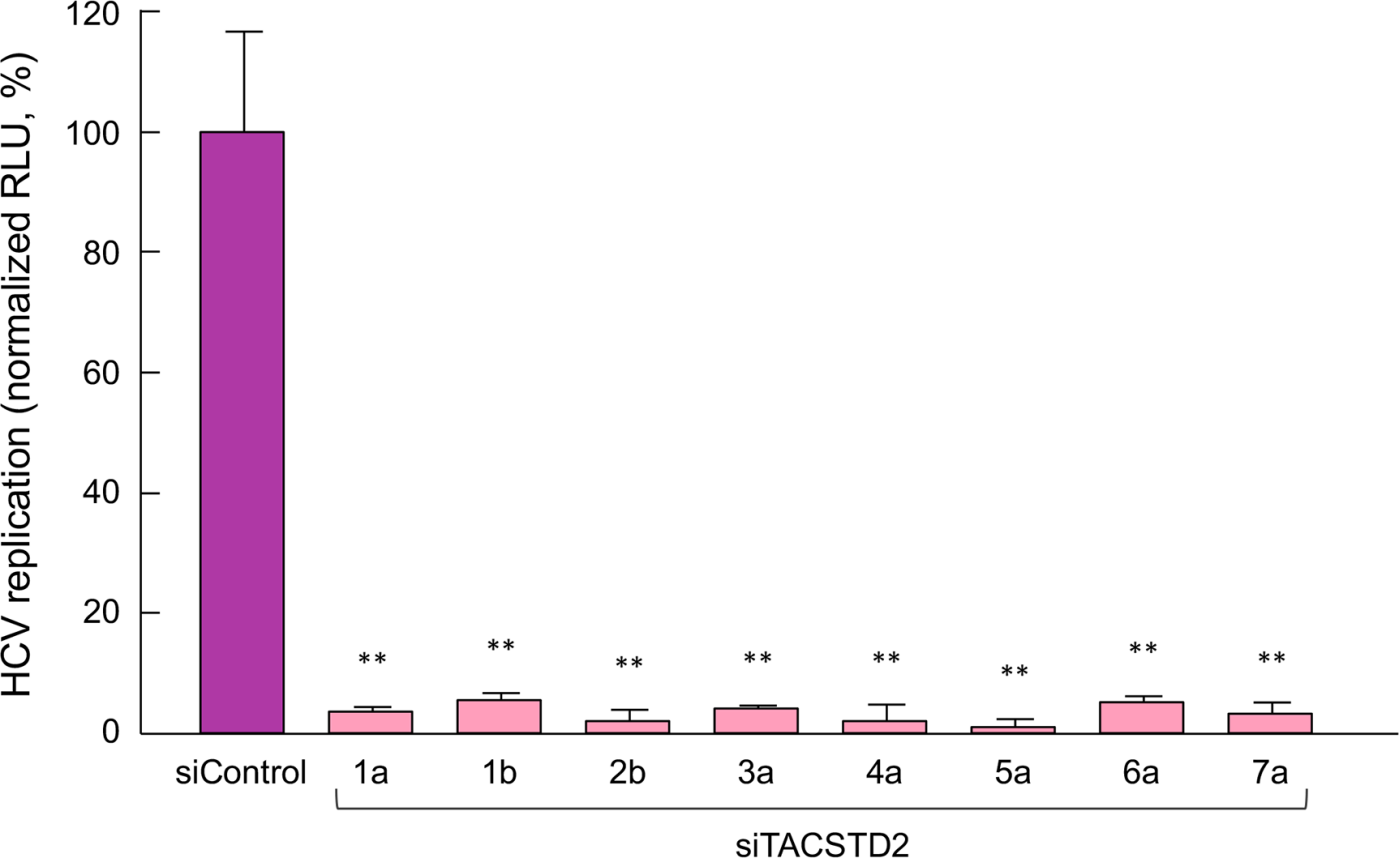
TACSTD2 gene silencing inhibits HCV infection with a pan-genotype effect. Parental Huh7.5 cells were transfected with either siControl or siTACSTD2 and after 72 h were infected with HCV of various genotypes, comprising 1a, 1b, 2b, 3a, 4a, 5a, 6a, and 7a. At 48 h post-infection, virus replication was assessed by measurement of luciferase activity. Values are normalized to relative light unit (RLU) from siControl-infected cells, and represent the mean ± SEM (n = 3). In all panels, asterisks indicate statistically significant differences versus siControl, by t-test (**P<0.01).

**Fig 7:**
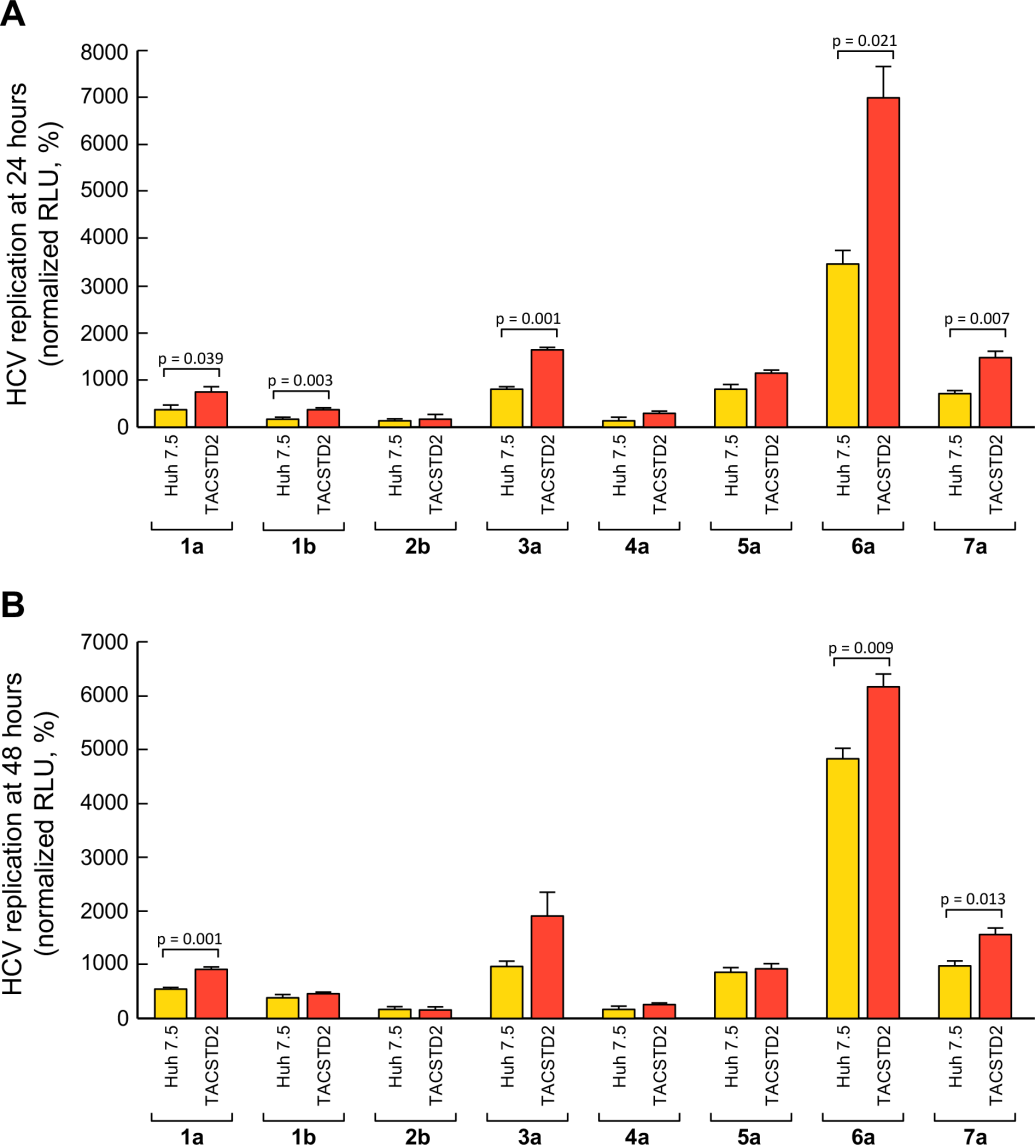
HCV infection in parental and TACSTD2 overexpressing Huh7.5 cells. Parental and TACSTD2 overexpressing Huh7.5 cells were infected with HCV 1a, 1b, 2b, 3a, 4a, 5a, 6a and 7a. At 24h (A) and 48h (B) post-infection, virus replication was assessed by measurement of luciferase activity. Values are represented as relative light units (RLU), and represent the mean ± SEM (n = 3). In both panels, statistically significant differences calculated by t-test between Huh 7.5 cells and TACSTD2 overexpressing cells are shown.

To study whether TACSTD2 affects infection by different HCV genotypes and subgenotypes, we tested the effect of *TACSTD2* gene silencing on infection by a series of luciferase-expressing chimeric viruses representing all HCV genotypes and two subgenotypes, namely, 1a, 1b, 2b, 3a, 4a, 5a, 6a, and 7a [38, 39]. Silencing of *TACSTD2* dramatically reduced infection by all the 8 chimeric HCV tested, regardless of their genotype, compared to the levels observed in siControl-treated cells (Fig 6). Next, we assessed whether HCV infection was more efficient in TACSTD2-overexpressing Huh7.5 cells by comparing infection by all HCV genotypes in TACSTD2-overexpressing vs. parental Huh7.5 cells. Our data show that HCV infection was consistently higher in TACSTD2-overexpressing cells, further corroborating the role of TACSTD2 in HCV infection. The difference was more marked at 24 hours of infection, and was statistically significant for genotypes 1a, 1b, 3a, 6a, and 7a, while it did not reach significance for genotypes 2b, 4a and 5a. In genotype 1a, 6a and 7a, HCV infection was significantly higher both at 24 and 48 hours post-infection (Fig 7). Finally, we evaluated whether HCV infection affects the levels of TACSTD2 expression during the course of HCV infection. We found that HCV infection did not significantly affect the expression of TACSTD2 (S14 Fig).

### TACSTD2 inhibits HCV infection at the level of viral entry

**Fig 8:**
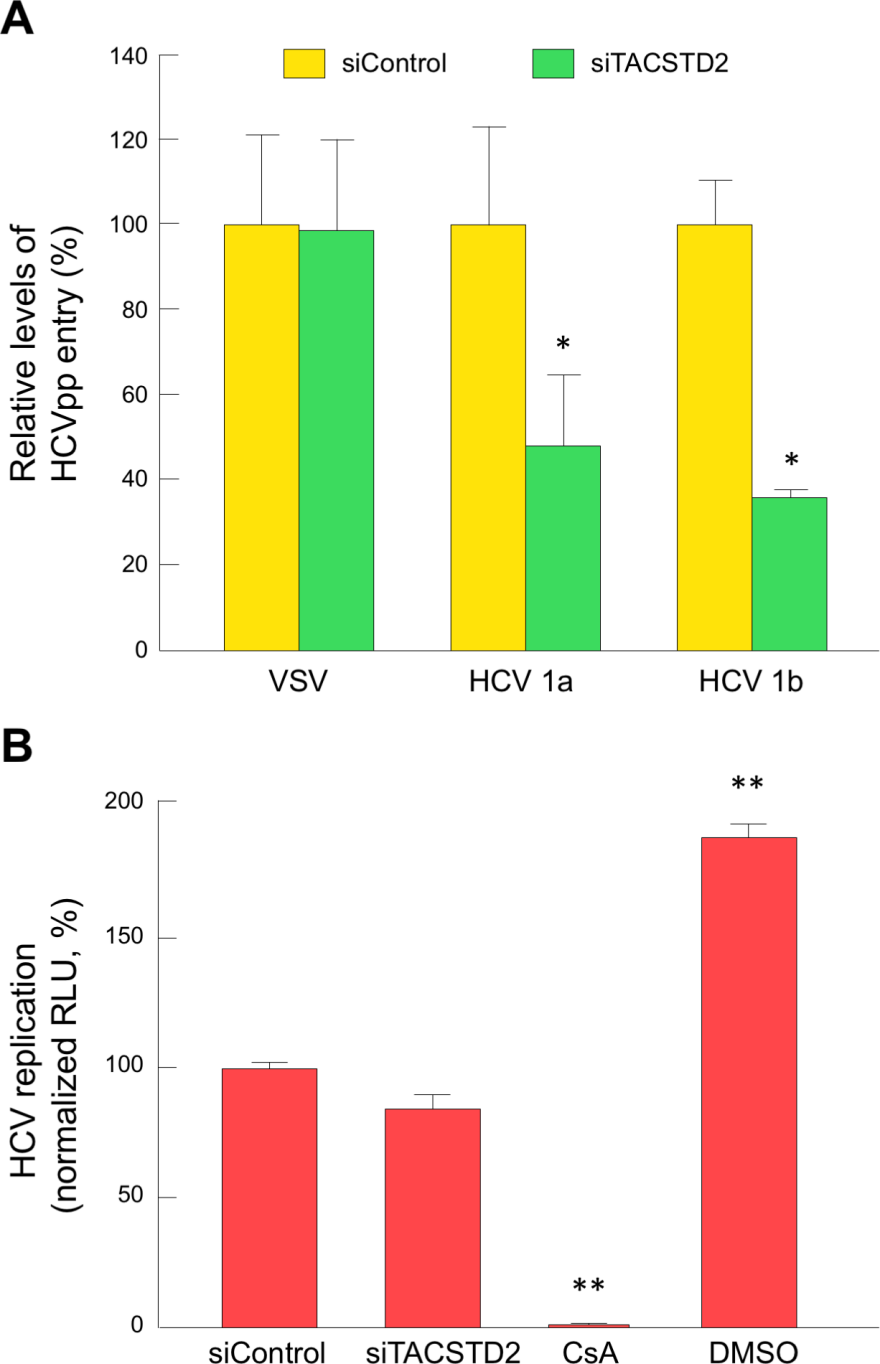
TACSTD2 inhibits HCV infection at the level of viral entry. (A) Parental Huh7.5 cells were transfected with either siControl or siTACSTD2 for 72h before infection. Cells were infected with VSVpp, genotype 1a HCVpp, or genotype 1b HCVpp, for 4h at 37°C. Following infection, HCVpp and VSVpp entry was assessed by measurement of luciferase reporter gene activity 72h post infection and expressed relative to entry in siControl-transfected cells. Data represent the mean ± SEM of triplicate experiments. A significant difference in the inhibition of HCVpp entry between siControl- and siTACSTD2-treated cells was detected by paired t-test. (B) Huh7.5 cells treated with siControl or siTACSTD2 for 72 h, were transiently transfected with wild-type HCV J6/JFH mRNA-RLuc (genotype 2a). CsA and DMSO were used as controls. At 48h post-transfection, virus replication was assessed by measurement of luciferase activity. Values are normalized to relative light unit (RLU) from siControl-infected cells, and represent the mean ± SEM (n = 3). In all panels, asterisks indicate statistically significant differences versus siControl, by t-test (*P<0.05; **P<0.01).

To elucidate if *TACSTD2* gene silencing inhibits HCV infection at the level of viral entry, we performed an HCV-entry assay using HCV pseudoparticles (HCVpp) derived from two HCV strains, subgenotypes 1a and 1b [34]. Silencing of *TACSTD2* significantly inhibited the entry of HCV pseudoparticles from both subgenotypes (Fig 8A), even though the inhibition was not greater than 60% most likely reflecting the fact that HCVpp cannot fully recapitulate the HCVcc entry process due to the inherent limitations of the assay. The specificity of these findings was confirmed by the lack of effect of *TACSTD2* silencing on the entry of vesicular stomatitis virus (VSV) pseudoparticles (Fig 8A). To evaluate if *TACSTD2* silencing may also affect HCV replication at the post-entry level, we transfected *in vitro-*transcribed full-length HCV genomes produced from HCV-Luc [J6/JFH(p7-Rluc2A)] [33] into siControl- and siTACSTD2-treated Huh7.5 cells. At 48h after transfection, no significant difference in HCV replication was detected in siControl- and siTACTD2-treated cells, while a significant suppression was seen upon treatment with Cyclosporin A (Fig 8B), indicating that TACSTD2 exerts no significant effects at the post-HCV entry level.

### Discussion

Our study identifies TACSTD2, a cell surface-expressed calcium-signaling receptor, as a novel host factor that is involved in the complex process of HCV entry through the regulation of CLDN1 and OCLN. This finding stems from a translational study that we previously conducted in patients with HCV-associated HCC, which documented a dramatic reduction in HCV RNA levels within malignant hepatocytes *in vivo* [18]. This diminished HCV replication was limited to malignant hepatocytes, as a decrease in HCV RNA was not detected in nontumorous areas of the liver, providing evidence that the surrounding nontumorous tissue can efficiently sustain HCV replication, regardless of the presence of HCC within the same liver. This was also confirmed by the detection of similar levels of HCV RNA in serum from patients with HCV-associated HCC and controls with non-HCC HCV cirrhosis [18]. The finding that HCV replication is restricted in malignant hepatocytes in primary tumor tissue is consistent with the inability or limited efficiency of primary HCV isolates to grow in hepatoma cell lines *in vitro* [2, 14]. To identify the factor(s) responsible for this restriction, we performed transcriptomic analysis taking advantage of a unique collection of liver specimens from well-characterized patients with HCV-associated HCC. Our comparative analysis showed a sharp change between the tumor periphery and the immediate perilesional area, and provided evidence that the hallmark of HCV HCC is a dramatic gene downregulation, as also reported for HBV HCC [40]. However, unlike HBV HCC, the most peculiar feature of HCV HCC is downregulation of genes related to both innate and adaptive immunity. Similar findings have been reported in previous studies [41–43]. The dramatic downregulation of genes associated with both innate and adaptive immune responses suggested that the diminished replication of HCV in the tumor tissue cannot be ascribed to immune-mediated control mechanisms. Thus, we postulated that malignant hepatocytes may express, or more likely may have lost factors that regulate HCV infection, and we focused on genes predicted to encode transmembrane proteins, which might be involved in the viral entry process. This analysis led us to the identification of TACSTD2 as one of the most likely candidates that might affect HCV entry because: (i) it was the second most downregulated gene within the tumor tissue; (ii) it showed a strong positive correlation with HCV RNA levels; and (iii) it had previously been linked with the regulation of CLDN1 localization and function in corneal epithelial cells [21]. Indeed, genetic defects in *TACSTD2* cause a loss of TJ function in the cornea, leading to gelatinous drop-like corneal dystrophy [44].

TACSTD2 functions as an intracellular calcium signal transducer [24] and is generally overexpressed in epithelial cancers such as pancreatic and gastric carcinoma [45]. In these tumors, TACSTD2 overexpression has been associated with poor prognosis and increased incidence of metastases [46, 47]. In contrast, there is very limited information on the role of TACSTD2 in HCC, but our results documented an inverse trend, with downregulation of this gene within HCC tissue. The only other epithelial cancer where TACSTD2 was found to be downregulated is lung adenocarcinoma [25]. To the best of our knowledge, there are no published studies on the role of TACSTD2 in HCC carcinogenesis, although recent reports have implicated TACSTD2 as an HCC-associated gene. In a recent study using a machine learning approach and various algorithms on a set of microarray data obtained from tumor and nontumorous liver specimens, TACSTD2 was found to be downregulated in the tumor versus nontumorous samples [48]. Another study, focused on genome-wide DNA methylation in various regions of the genome in HCC and adjacent nontumorous tissues, found the TACSTD2 promoter locus to be hypermethylated in HCC samples [49], which is consistent with downregulation of this gene and could explain the very low expression of TACSTD2 detected in our HCC samples. Thus, both of these studies independently corroborate our findings of TACSTD2 downregulation in HCC.

The results of our gene silencing experiments demonstrated that TACSTD2 is involved in the maintenance of the proper cellular localization of two TJ proteins that are essential for HCV entry, CLDN1 and OCLN, both in hepatoma cells and in primary human hepatocytes. We investigated the molecular mechanism underlying these effects and found that TACSTD2 interacts with both CLDN1 and OCLN, as shown by co-IP studies, and regulates PKC-mediated phosphorylation of these two proteins, which is critical for their proper cellular localization, as documented by different groups [27, 30, 31, 50–52]. In this respect, it is interesting that PKC inhibitors were previously demonstrated to inhibit the replication of HCV RNA [53]. Furthermore, TACSTD2 itself is phosphorylated by PKC at a serine residue (S_303_) located within a conserved phosphatidylinositol 4, 5-bisphosphate (PIP_2_)-binding motif, which is important for protein-protein interaction and is involved in numerous intracellular signaling pathways [21, 54, 55]. Different isoforms of PKC have been reported to affect TACSTD2 [56]. In the present study, we documented that TACSTD2 is phosphorylated also in hepatoma cells and showed that the phosphorylated form of TACSTD2 interacts with CLDN1 and OCLN. Thus, there is a complex regulatory network involving PKC, TACSTD2, CLDN1 and OCLN.

In line with the disruption of CLDN1 and OCLN localization induced by *TACSTD2* gene silencing, we provide evidence that TACSTD2 is involved in the complex process of HCV entry. Using chimeric viruses from all HCV genotypes and two subgenotypes, we found that TACSTD2 silencing exerted a pan-genotype inhibitory effect on HCV infection, which is consistent with a mechanism mediated by disruption of CLDN1 and OCLN, as all of these genotypic variants are dependent on these HCV-entry cofactors for entry into hepatocytes. As evidenced by HCVpp assays, blockade of HCV infection upon *TACSTD2* silencing occurs at the level of viral entry. Moreover, by comparing the efficiency of HCV infection by all genotypes in parental versus TACSTD2-overexpressing Huh7.5 cells, we found that it was consistently higher in TACSTD2-overexpressing cells than in parental Huh-7.5 cells, further corroborating the role of TACSTD2 in HCV infection. Altogether, these results highlight the critical role of TACSTD2 in the HCV-entry process through regulation of CLDN1 and OCLN.

A unique feature of our study is that the clinical and biological relevance of TACSTD2 as a regulator of HCV entry is corroborated by a series of *in vivo* observations that we made in primary tumor tissue obtained at the time of liver explant or resection from well-characterized patients with HCC. One of the most striking findings in the tumor tissue was the disruption of the typical linear CLDN1 and OCLN distribution along the cellular membrane, associated with a dramatic restriction of HCV replication, whereas the patterns of expression of other HCV-entry factors such as SR-B1 and CD81 were not altered. Gene expression profiling demonstrated that none of these HCV-entry factors, i.e., CLDN1, OCLN, SR-B1 and CD81, were differentially expressed within the tumor compared to the surrounding nontumorous tissue, indicating that the abnormal distribution of CLDN1 and OCLN occurs at the post-transcriptional level. Interestingly, among the known host factors involved in HCV entry, including those more recently described, such as EGFR, LDLR, TfR1, SRFBP1, and E-cadherin, none was significantly downregulated within the tumor with the exception of TACSTD2. The marked disruption of CLDN1 and OCLN localization that we observed in both hepatoma cells and primary human hepatocytes upon *TACSTD2* gene silencing recapitulates the *in vivo* observations and strongly suggests that downregulation of *TACSTD2* in the tumor of HCV-infected HCC patients may be responsible for the altered localization and function of these two HCV-entry cofactors within malignant hepatocytes.

In conclusion, our study identified TACSTD2 as a novel host factor that participates in the complex mechanism of HCV entry into hepatocytes. This protein is involved in the maintenance of the proper cellular localization of the two major HCV-entry cofactors, CLDN1 and OCLN, within hepatoma cells and primary human hepatocytes. A highlight of this study is that the altered distribution of CLDN1 and OCLN that we observed upon *TACSTD2* gene silencing *in vitro* is analogous to the pattern that we documented *in vivo* in primary liver tissues from patients with HCV-associated HCC, where HCV replication is severely restricted. Although a causal relationship between restricted HCV replication and disruption of CLDN1 and OCLN localization is difficult to establish in the absence of a precise knowledge of the mechanism whereby these two TJ proteins mediate HCV entry, our results demonstrate a strong association between TACSTD2 downregulation, abnormal CLDN1 and OCLN localization and restricted HCV replication both *in vitro* and *in vivo*. The mechanistic interaction of TACSTD2 with CLDN1 and OCLN that we documented in hepatocytes contributes to our understanding of the complex process of HCV entry and may assist in the development of more efficient cellular systems for HCV propagation *in vitro*. The identification of TACSTD2 as a novel host factor that regulates two major HCV entry cofactors may have relevance for the pathogenesis, treatment and prevention of HCV infection.

## Materials and methods

### Study subjects and design

We studied multiple liver specimens obtained from a cohort of 8 patients who underwent OLT or partial hepatectomy for HCV-associated HCC between 2006 and 2010 at the Liver Transplantation Center of the Brotzu Hospital in Cagliari, Italy. We analyzed up to 17 liver specimens for each of the 8 patients, taken in all 4 directions, termed north (N), south (S), east (E), and west (W) for simplicity, starting from the center of the tumor. Specifically, the design included 5 biopsies from the tumor, one at the center (A) and 4 in the periphery of the tumor (B: N, S, E and W); 4 biopsies from the perilesional area (C: N, S, E and W); 4 biopsies taken 2–3 cm from the tumor (D: N, S, E and W); and 4 biopsies from the edges of the liver (E: N, S, E and W). In some cases, however, collection of nontumorous liver specimens at all distances and directions from the center of the tumor was not possible because of the location of the tumor. On average, 14 liver specimens were collected from each of the 8 patients for a total of 110 liver samples (45 from the tumor and 65 from the nontumorous tissue). Each sample was divided into two pieces: one was snap-frozen for molecular studies and the other was formalin-fixed and paraffin-embedded (FFPE) for pathological examination. Importantly, when FFPE sections obtained from the tumor or the perilesional area showed a mixed population of tumor and non-tumor cells, the corresponding frozen liver specimens were excluded from microarray analysis. Of the 110 liver specimens analyzed, 6 were excluded for the presence of a mixed cell population. We have also studied a control group represented by normal liver, which comprises 6 liver donors and 6 subjects who underwent liver resection for hepatic hemangioma; all had normal liver histologically documented, and all were negative for serologic markers of active infection with hepatitis viruses. Their mean age was 38.3 years, 5 were males and 7 females.

### Ethics statement

All patients were seen at the Liver Transplantation Center of the Brotzu Hospital in Cagliari, Italy. Liver specimens and sera were obtained at the time of OLT or partial hepatectomy for HCV-associated HCC. All patients provided written informed consent, and the protocol was approved by the ethical Committee of the Brotzu Hospital, Cagliari, Italy. The study was also approved by the Office of Human Subjects Research of the National Institutes of Health, Bethesda, MD, on the condition that all samples were de-identified.

### Gene expression profiling

Gene expression profiling was performed on up to 17 liver specimens obtained at various distances from the tumor center for each of the 8 patients, including 5 liver specimens from the tumor and 12 from the surrounding nontumorous tissue. All liver specimens were analyzed by microarray using Affymetrix Human U133 Plus 2.0 arrays, which contain 54,675 transcripts representing approximately 38,500 unique human genes. Total RNA from liver tissue was extracted from frozen liver specimens as previously described [57] using TRIzol reagent (Invitrogen, Carlsbad, CA, USA) according to the manufacturer’s recommendations; Total RNA quality and integrity were assessed using the Agilent 2100 Bioanalyzer (Santa Clara, CA, USA). Total liver RNA (50 ng) obtained from liver tissue was subjected to two successive rounds of amplification [58], and the resultant RNA was then subjected to biotin labeling, hybridization, staining, washing, and scanning procedures according to standard Affymetrix protocols.

### Statistical analyses

Microarray data were analyzed using BRB-Array Tools Version 4.2 [59] as previously reported [57]. Briefly, microarray raw data (.CEL files) were summarized and normalized by the RMA method. Transcripts showing minimal variation (less than 1.5-fold deviations from the median in more than 80% of the arrays) were excluded from the analysis. Preliminary tests by Anova mixed model showed a prominent effect of the relative distance of the samples (A, B, C, D, and E) from the center of the tumor, whereas the direction of samples (N, S, E and W, relative to the center of the tumor) had no effect on gene expression. Thus, data from samples obtained in the 4 directions of the same liver area were averaged to increase the power of subsequent statistical analyses. To identify genes that were differentially expressed in the tumor, the A and E liver areas (corresponding to the tumor and the most distant nontumorous tissue, respectively) were compared by a t-test with a false discovery rate (FDR) <5% with 80% confidence level. The five liver areas were also globally compared by a multivariate permutation F-test with a FDR <1% with 80% confidence level. The heatmap of the gene expression profiles of the five liver areas was obtained after ordering genes by the average clustering method, using 1-correlation as distance. Fold changes were calculated as the ratio between the geometric means of these two areas. Fold changes < 1 were converted to the inverse ratio with negative sign. Pathway and functional analyses were performed using IPA v 9.0 (Ingenuity Pathway Analysis, http://www.ingenuity.com/). The association of genes to IPA pathways was evaluated by Fisher’s exact test. Correlations between gene expressions and HCV RNA levels were evaluated by the Pearson product-moment correlation coefficient.

### Real-time quantitative PCR

Reverse transcriptase-quantitative real-time polymerase chain reaction (RT QPCR) was used to validate genes found to be differentially expressed between tumor and nontumorous tissue. We used total RNA obtained from liver tissue that was previously employed for gene expression profiling. Reverse transcription was performed with an ABI high capacity cDNA Synthesis Kit (Applied Biosystems, Foster City, CA) and 40ng of cDNA was used for each target in the TaqMan QPCR method. We used commercially available primers (ABI) for the reference housekeeping gene (GAPDH) and all the targets except TACSTD2, for which alternate primers were employed (primer sequences available upon request). Reaction mixtures included TaqMan 2x Universal PCR Master Mix, target primer mix and samples, all used at the manufacturer’s recommended concentrations. PCR was carried out using an ABI PRISM 7900HT Sequence Detection System (Applied Biosystems, Foster City, CA). Cycling conditions included 2 minutes at 95°C followed by 40 cycles of 5 seconds denaturation at 95°C, and 30 seconds annealing/extension at 60°C. Samples were tested in triplicate and the results were normalized to the non-tumor tissue, expressed as 2^-ΔΔCT^, where ΔΔC_T_ is the average difference between the target tissue (tumor or non-tumor) ΔC_T_, and the reference tissue (non-tumor) ΔC_T_. ΔC_T_ is the average difference between the target gene C_T_ and the reference gene (GAPDH) C_T_ [60].

### Immunofluorescence, confocal microscopy and imaging analysis of human tissues

Intrahepatic expression of the HCV-entry cofactors CLDN1, OCLN, SR-B1, and CD81 was investigated by confocal microscopy in 8 patients with HCV-associated HCC. We analyzed 4 liver specimens for each patient, including two from the tumor (A and B) and two from the surrounding nontumorous tissue, comprising the perilesional area (C) and the most distant nontumorous area (E) along a single direction. We examined a total of 16 liver tumor and 16 of nontumorous specimens. As negative controls, we used FFPE liver sections obtained from 3 liver donors who showed normal liver histology and were negative for markers of infection with hepatitis viruses. To detect intrahepatic CLDN1, OCLN, CD81 and SR-B1, representative FFPE sections of 3 to 5μm were heated overnight at 37°C, subsequently deparaffinized by xylene, and rehydrated in successive graded alcohol to distilled water. Antigen retrieval was performed with a pressure cooker (Dako, Glostrup, Denmark) by submerging sections in 1mM EDTA (Phoenix); the sections were then heated at 125°C at 21 psi for 3 minutes. Non-specific binding sites were blocked with a solution of 10% goat serum and 0.1% saponin for 30 minutes. Subsequently, the slides were stained for 1 hour with 2.5μg/mL rabbit polyclonal anti-human CLDN1, 5μg/mL mouse monoclonal anti-human OCLN (Zymed, Invitrogen, Carlsbad, CA, USA). Slides were also stained by mouse monoclonal anti-human CD81 (5μg/mL) and by mouse monoclonal anti-human SR-B1 (2.5μg/mL). After rinsing with 1X phosphate buffered saline (PBS), sections were incubated with a 1:400 dilution of the corresponding secondary antibody, Alexa Fluor 488 goat anti-rabbit IgG, Alexa Fluor 568 F(ab’) 2 fragment of goat anti-mouse IgG, or Alexa Fluor 647 goat anti-rat IgG (all from Invitrogen, Carlsbad, CA, USA) for 1 hour. After 3 washes with 1X PBS, samples were stained with DAPI (Invitrogen) and mounted with ProLong Gold mounting medium (Invitrogen, Carlsbad, CA, USA). Samples were kept overnight in the dark before observation with a confocal microscope. Images were obtained using a Leica SP5 X-WLL (Leica Microsystems, Exton, PA, USA) equipped with the 63X oil immersion objective NA 1.4. DAPI was excited using UV 405 laser, whereas the other fluorophores were excited with a white light laser with a range of wavelengths (470-670nm). To avoid emission crosstalk, sequential frame averaged scans were set up for each fluorophore. Data were deconvolved with Huygens Essential software (version 14.10.2-p8, Scientific Volume Imaging BV, Hilversum, Netherlands). Sequential Z-sections of stained cells were acquired for 3D reconstruction and surface modeling with the Imaris software (version 8.2.0, Bitplane AG, Zurich, Switzerland). The number of negative and positive cells was determined using the combination of spot, surface and masking functions of Imaris; statistical data were calculated from multiple samples (at least 4 images per section) for each experiment.

### Cells

Huh7.5 cells, kindly provided by C. Rice, were maintained in Dulbecco’s modified Eagle’s medium (DMEM) with 10% FBS and 2mM L-glutamine. TACSTD2-overexpressing cells were generated by transfecting Huh7.5 cells with pcDNA3.1 vector carrying TACSTD2 gene insert using Fugene 6 Transfection Reagent (Promega) according to the manufacturer’s instructions. To obtain a stable cell line, cells were cultured under Geneticin antibiotic selection for 2–3 weeks. Primary human hepatocytes were purchased from Life Technologies and maintained in William’s E media. Cell proliferation was assessed using the division-tracking vital dye CFSE (Thermo Fisher). Parental or TACSTD2-overexpressing Huh7.5 cells were labeled with CFSE at the final concentration of 0.5 μM for 10 min at room temperature, then washed three times in DMEM containing 10% FBS and resuspended in the same medium. Labeled cells were cultured in 12-well plates in duplicate wells for each condition and time point. The proportion of proliferating cells was scored after 24, 48 and 72 hours by evaluating the reduction in CFSE fluorescence intensity by flow cytometry.

### Immunofluorescence, confocal microscopy and imaging analysis of cultured Huh7.5 cells and primary human hepatocytes

Cells were fixed in chilled methanol at -20°C for 10 minutes, followed by 1XPBS wash. Cells were treated with a blocking solution of 0.5% BSA, 0.5% milk and 0.1% Triton x-100 for 20 minutes at room temperature. Cells were incubated with primary antibodies: anti-TACSTD2 (1:100), anti-CLDN1 (1:100), anti-OCLN (1:100), anti-ZO-1 (1:100), anti-JAM-A (1:100), anti-E-cadherin (1:100) and HCV anti-core (1:100) for 2h at room temperature followed by washing three times with 1XPBS. FITC or Alexa-594 conjugated secondary antibodies (Invitrogen) (1:500) were applied for 1 h at room temperature, and then cells were washed again three times with 1XPBS. Slides were mounted in Prolong Gold (Invitrogen) containing 1μg/ml DAPI (4', 6'-diamidino-2-phenylindole). Images were collected with a Leica TCS-SP5 laser scanning confocal imaging system.

### Reagents

A pcDNA3.1 vector carrying the TACSTD2 gene insert and a pcDNA3.1 vector carrying the siRNA-resistant TACSTD2 gene devoid of the 3’- and 5’-untraslated regions (UTR) (pTACSTD2) for rescue experiments were purchased from GenScript Corporation. siRNA for TACSTD2 gene silencing (siTACSTD2) targeting the 3’UTR of the TACSTD2 gene was performed using SMARTpool of 4 siRNAs (Dharmacon) (siTD2-A, 5’-GAGAAGAGGAGUUUGUUAAUU-3’; siTD2-B, 5’-ACAAGUAUCUGUAUGACAAUU-3’, siTD2-C, 5’-GCAAGUAACUGAAUCCAUUUU-3’, siTD2-D, 5’-GCACACACCAGGUUUAAUAUU-3’), which were transfected into parental and TACSTD2-overexpressing Huh 7.5 cells at 100nM final concentration using RNAiMAX (Invitrogen). The antibodies used include mouse anti-TACSTD2 (Santa Cruz), goat anti-TACSTD2, biotinylated goat anti-TACSTD2 and goat anti-E-cadherin, mouse IgG1 isotype control, mouse IgG2A isotype control, and normal goat IgG control (R&D systems), mouse anti-CLDN1 (Abnova), rabbit anti-CLDN1 and mouse anti-OCLN (Life Technologies), rabbit anti-OCLN (Abcam), rabbit anti-CD81 and mouse anti-ZO-1 (Thermo Scientific), mouse anti-SR-B1 (BD transduction laboratories), mouse anti-CD81 (BD Pharmingen), mouse anti-JAM-A (Hycult Biotech), mouse anti-HCV core (Anogen), rabbit anti-PKC substrate (Cell Signaling Technologies), and rabbit anti-alpha tubulin (Millipore). The HCV neutralizing mAb AR4A was a gift from Dr. Mansun Law.

### Immunoblotting

Cellular proteins were extracted in Pierce IP lysis buffer (25 mM Tris•HCl pH 7.4, 150 mM NaCl, 1% NP-40, 1 mM EDTA, 5% glycerol) with cOmplete protease inhibitor (Roche) on ice for 30 minutes. Protein concentration was determined using the bicinchoninic acid (BCA) protein assay kit (Pierce) according to manufacturer’s instructions. For each sample, 20μg of protein was separated by SDS-polyacrylamide gel electrophoresis (PAGE) and electrotransferred onto Immobilon-P membranes (Millipore). Proteins were detected with different primary antibodies followed by horseradish peroxidase-conjugated secondary antibodies. Proteins were detected on the membrane with the chemiluminescent reagent SuperSignal West Dura (ThermoScientific).

### Immunoprecipitation

To investigate TACSTD2 interaction with CLDN1 and OCLN in hepatoma cells we performed co-immunoprecipitation followed by immunoblotting. Cell extracts were prepared in Pierce IP lysis buffer containing cOmplete protease inhibitor cocktail (Roche) and PhosSTOP (Roche). Protein concentration was determined using the BCA protein assay kit (Pierce). Equal amounts of total protein were immunoprecipitated using either anti-TACSTD2 or anti-CLDN1 or anti-OCLN antibodies at 4°C overnight. Then, protein-G sepharose beads (Sigma) were added for 2h at 4°C. Immune complexes were washed five times with IP lysis buffer and proteins were eluted in 50μl of lithium dodecylsulfate (LDS) sample buffer. Protein deglycosylation was performed on the IP eluates using a protein deglycosylation kit (New England Biolabs) according to the manufacturer’s instructions prior to separation on a 4–12% gradient polyacrylamide gel electrophoresis gel (Invitrogen). Immunoblotting was performed as described above.

### HCV infection

The HCV infection assay was performed with the reporter gene-expressing virus HCV-Luc that contains the coding sequence for Renilla luciferase (kindly provided by T.J. Liang). HCV Luc was produced by transfecting in vitro-transcribed mRNA with the whole HCV genome from the plasmid pJ6/JFH(p7-Rluc2A) (a kind gift from C. Rice), as previously reported [33]. Huh 7.5 cells were seeded into 24-well plates at a density of 50,000 cells per well. After overnight culture, the cells were transfected with 100 nM of siControl or siTACSTD2, or co-transfected with 100 nM siTACSTD2 plus 15 ng of the siRNA-resistant TACSTD2 rescue plasmid using the RNAiMAX transfection reagent (Invitrogen). At 72h post-transfection, the cells were infected with the reporter gene-expressing virus HCV-Luc with or without the HCV-neutralizing mAb AR4A (50 μg/ml) (kindly provided by M. Law) [35] or mock infected with cell culture medium alone. MAb AR4A was incubated with the HCV stock for 1h at 37°C before addition to the cells [35]. After incubation for 4h at 37°C, virus-containing medium was removed, the cells were washed three times with cold PBS and then incubated with complete medium with or without 10 μM of the HCV replication inhibitor cyclosporin A [34] in DMSO or with DMSO alone as a control. At 24h, 48h, and 72h post-infection, the cells were lysed using reporter lysis buffer and analyzed for luciferase activity using the Renilla luciferase assay system (Promega E2810) according to the manufacturer’s instructions.

To perform infections with HCV chimeric viruses, Huh 7.5 cells were seeded into 24-well plates at a density of 50,000 cells per well and transfected with 100 nM of siControl or siTACSTD2, as described above. At 72h post-transfection, the cells were infected with chimeric HCV viruses (Rluc) 1a, 1b, 2b, 3a, 4a, 5a, 6a, and 7a (kindly provided by J. Bukh) [38, 39]. After incubation for 4h at 37°C, the virus-containing medium was removed, the cells were washed three times with cold PBS and then incubated with complete medium. At 48h post infection, the cells were lysed using a reporter lysis buffer, and lysates were analyzed for Renilla luciferase activity according to the manufacturer’s instructions (Promega E2810).

### HCV infection for immunofluorescence imaging

Huh7.5 cells were seeded onto coverslips in 12 well plates at a density of 100,000 cells per well. Cells were transfected with either 100nM of control siRNA or TACSTD2 siRNA for 72h prior to HCV infection, using the RNAiMAX transfection reagent (Invitrogen). On the day of infection, cells were infected with HCV, strain JFH1-AM2 at a multiplicity of infection (MOI) of 1 for 4h at 37°C [37]. This JFH1-AM2 strain, which was derived from the original JFH1 virus upon serial passage in Huh-7.5 cells, is characterized by a 3–4-log greater yield of infectious virions compared to the wild type virus [37]. Following infection, fresh medium was added. The cells were fixed with ice-cold methanol for 10 min at -20°C either at 24h or 48h post infection, and stained with mouse anti-HCV core (Anogen) for immunofluorescence imaging and analysis. Images were collected with a Leica TCS-SP5 laser scanning confocal imaging system (Leica Microsystems, Exton, PA). Image analysis was performed on sequential sections of stained cells that were acquired using a confocal microscope (Leica SP5, Leica Microsystems). A 3D volume was constructed from sequential z-sections of the cells using Imaris software. For image analyses in Imaris (version 7.7.2, Bitplane AG, Zurich, Switzerland), 3D surface feature of Imaris was used to eliminate background and calculate the intensity of the cells for statistical analysis. The same threshold was applied to each data set. Intensities of the samples were normalized for cell numbers and sample volumes. The standard error was calculated for each data set.

### HCV RNA transfection

Huh 7.5 cells were treated with TACSTD2 siRNA or Control siRNA for 72h and then transfected with in vitro-transcribed full-length HCV RNA produced from pJ6/JFH(p7-Rluc2A) [33] using the DMRIE-C transfection reagent (Thermo Fisher) according to the manufacturer’s instructions. Briefly, cells were washed with serum free medium, then RNA/DMRIE-C mix was added to the cells and incubated for 4h at 37°C. After 4h, the RNA/DMRIE-C mixture was removed and the cells were replenished with complete medium or medium containing 10 μM cyclosporin A or DMSO. At 48h post HCV RNA transfection, the cells were lysed using lysis buffer and analyzed for luciferase activity using the Renilla luciferase assay system (Promega E2810) according to the manufacturer’s instructions.

### RNA extraction and quantification of HCV RNA

Total RNA was extracted from stored frozen liver specimens using TRIzol reagent (Invitrogen) according to the manufacturer’s recommendations. Intracellular RNA was extracted from whole-cell lysates by using the RNeasy Mini Kit (Qiagen). HCV RNA levels were quantified using TaqMan real-time PCR, as reported previously [61]. The primers and probe were derived from the highly conserved 5’untranslated region of the HCV genome. Fifty ng of total liver RNA were tested in each reaction and results were expressed as log_10_ International Units (IU) per nanogram, based on the WHO 96/790 reference standard [62]. A standard curve comprising a 6-log dynamic range was constructed with the OptiQuant HCV RNA (Acrometrix, Benicia, CA) nucleic acid reference panel.

### HCV pseudoparticle entry assay

The HCV entry assay was performed with HCV pseudoparticles (pp)-1a and 1b, or VSV-Gpp as a control (kindly provided by T.J. Liang). Pseudoparticles were generated by co-transfection of 293T cells with an envelope-deficient HIV vector carrying the luciferase reporter gene (pNL4-3.Luc.R^-^E^-^) and a plasmid encoding HCV genotype 1a or 1b envelope proteins, or VSV-G envelope protein, as previously reported [63, 64]. Huh 7.5 cells were seeded onto a 96 wells plate (10^4^ cells/well) and transfected with 100nM of either control siRNA or TACSTD2 siRNA using the RNAiMAX reagent (Invitrogen). At 72h after transfection, the cells were infected with HCVpp-1a and HCVpp-1b or VSV-Gpp for 4h at 37°C, and then fresh medium was added. HCVpp and VSV-Gpp entry was assessed by measurement of luciferase reporter gene activity 72h post-infection, and the levels of virus entry in siTACSTD2-treated cells were expressed relative to the levels of infection in siControl-treated cells.

### Accession numbers

Microarray data are available at the Gene Expression Omnibus (http://www.ncbi.nlm.nih.gov/geo/, accession number GSE 78737, which includes tumor and nontumorous liver specimens, and hepatoma cell lines.

## Supporting information

S1 Fig Functional analysis of genes differentially expressed in HCC-associated HCV. (A) Top-scored pathways, and (B) diseases and bio functions identified by IPA database (Ingenuity Pathway Analysis, http://www.ingenuity.com/) from the set of genes differentially expressed in HCV-associated HCC. Columns display the P-value calculated by Fisher’s exact test (left axis). Dots indicate the percentage of downregulated genes (right axis). All categories show a striking majority (on average 90%) of downregulated genes.
(PDF)

S2 Fig Heatmap of 87 immune genes differentially expressed in liver samples from tumor and nontumorous tissues of 8 patients with HCV-associated HCC. Gene expression levels were log2-transformed and row-wise standardized. Up-regulated genes are shown in shades of red; downregulated genes in shades of green. The dynamic range of colors is ± 1 SD.
(PDF)

S3 Fig Expression levels of known host factors involved in regulating HCV entry in liver samples from tumor and nontumorous tissues of 8 patients with HCV-associated HCC. Statistical significance (FDR 5%) was assessed by multivariate permutations. None of the host factors analyzed was differentially expressed, with the exception of TACSTD2, which was significantly downregulated within the tumor (P<0.00001).
(PDF)

S4 Fig Visualization of SRB1 and CD81 distribution in tumor and nontumorous tissue of a representative patient by immunofluorescence staining. To enhance the structural features, SR-B1 and CD81 are shown as red surface rendering, while nuclei are shown as blue volume rendering. No significant difference was observed in the distribution of SR-B1 and CD81 between the tumor and nontumorous tissue.
(PDF)

S5 Fig SEC14L2 expression, TACSTD2 expression, and HCV RNA concentration. (A) No correlation was found between SEC14L2 gene expression and HCV replication levels in tumorand nontumorous tissue. (B) A significant correlation was found between TACSTD2 gene expression and HCV replication levels in tumor and nontumorous tissue.
(PDF)

S6 Fig Localization of TACSTD2 (green) and CLDN1 (red) in untransfected parental and TACSTD2-overexpressing Huh7.5 cells by immunofluorescence staining. In TACSTD2- overexpressing cells both proteins are co-localized along the cellular membrane.
(PDF)

S7 Fig Lack of interaction of TACSTD2 with the retinoblastoma (Rb) protein. Cell lysates from parental and TACSTD2-overexpressing Huh7.5 cells were immunoprecipitated with anti- TACSTD2 antibody or control IgG, deglycosylated, and probed with anti-TACSTD2 or anti-Rb antibodies. No interaction was detected between TACSTD2 and Rb in the immunoprecipitated complex, although the proteins could be detected in the unbound fraction in the supernatant following immunoprecipitation. WCL denotes whole cellular lysate.
(PDF)

S8 Fig Effect of TACSTD2 gene silencing on CLDN1 and OCLN distribution in TACSTD2-overexpressing Huh7.5 cells. (A) Visualization of TACSTD2 (green) and CLDN1 (red) in TACSTD2-overexpressing Huh7.5 cells transfected with either siControl or siTACSTD2. CLDN1 (red) appears speckled and fragmented in siTACSTD2-treated cells, which show a complete loss of TACSTD2 staining, in contrast to the regular CLDN1 linear pattern observed in siControl-treated cells. (B) Visualization of TACSTD2 (green) and OCLN (red) in parental Huh7.5 cells transfected with siControl or siTACSTD2. OCLN (red) appears speckled and fragmented in siTACSTD2-treated cells, which show a complete loss of TACSTD2 staining, in contrast to the linear pattern observed in siControl-treated cells. (C) Parental Huh7.5 cells were transfected with siTACSTD2 or siControl and labeled with the division-tracking vital dye CFSE. The proportion of cells that underwent more than two replication cycles at 24, 48 and 72 hours was recorded by flow cytometry. Data represent the mean ± SEM of duplicate wells. No significant difference in proliferation was observed between cells treated with siTACSTD2 orsiControl. (D) Huh7.5 cells (Huh7.5) or TACSTD2-overexpressing cells (Huh7.5 TACSTD2) were labeled with the division-tracking vital dye CFSE. The proportion of cells that underwent more than two replication cycles at 24, 48 and 72 hours was recorded by flow cytometry. Data represent the mean ± SEM of duplicate wells. No significant difference in proliferation was observed between the two cell lines.
(PDF)

S9 Fig TACSTD2 gene silencing in parental Huh 7.5 cells and its effect on tight junction protein distribution. (A) Immunoprecipitation with an anti-TACSTD2 antibody following TACSTD2 gene silencing showed a visible TACSTD2 band in Huh7.5 cells transfected with siControl but not with siTACSTD2. (B) Visualization of CLDN1, OCLN, and ZO-1 in parental Huh7.5 cells transfected with either siControl or siTACSTD2. All three tight junction proteins appear disrupted in siTACSTD2-treated cells in contrast to their regular linear pattern observed in siControl-treated cells. (C) Quantitative RT-PCR data showing relative levels of TACSTD2 mRNA in siControl- and siTACSTD2-transfected parental Huh 7.5 cells. Data are expressed as 2-ΔΔCT, where ΔΔCT is the average difference between the siTACSTD2 ΔCT and siControl ΔCT.
(PDF)

S10 Fig Effect of TACSTD2 gene silencing on ZO-1 and JAM-A distribution in parental Huh7.5 cells. (A) Visualization of ZO-1 (red) in parental Huh7.5 cells transfected with either siControl or siTACSTD2. ZO-1 appears disrupted in siTACSTD2-treated cells in contrast to the regular ZO-1 linear pattern observed in siControl-treated cells. (B) Visualization of JAM-A (red) in parental Huh7.5 cells transfected with siControl or siTACSTD2. JAM-A (red) appears disrupted in siTACSTD2-treated cells in contrast to the linear pattern observed in siControltreated cells.
(PDF)

S11 Fig Effect of TACSTD2 gene silencing on expression and distribution of E-cadherin in parental Huh7.5 cells. (A) Detection of TACSTD2 in parental Huh7.5 cells by immunoprecipitation with an anti-TACSTD2 antibody followed by deglycosylation. (B) *TACSTD2* gene silencing did not affect the expression of E-cadherin in parental Huh7.5 cells. Cell lysates from parental Huh7.5 cells were analyzed by Western blot with anti-E-cadherin 72h after transfection with either siControl or siTACSTD2. (C) Visualization of E-cadherin (red) in parental Huh7.5 cells transfected with either siControl or siTACSTD2. The cellular localization of E-cadherin in siTACSTD2-transfected Huh7.5 cells was similar to that observed in siControltransfected cells.
(PDF)

S12 Fig Effect of TACSTD2 gene silencing on E-cadherin and vimentin mRNA expression. Quantitative RT-PCR data showing relative levels of TACSTD2, E-cadherin and vimentin mRNA in siControl- and siTACSTD2-treated parental Huh 7.5 cells. Data are expressed as 2- ΔΔCT, where ΔΔCT is the average difference between the siTACSTD2 ΔCT and siControl ΔCT. An increase in E-cadherin and decrease in vimentin levels were observed following siTACSTD2 gene silencing.
(PDF)

S13 Fig Effect of TACSTD2 gene silencing on CLDN1 and OCLN mRNA and protein expression. (A) Quantitative RT-PCR data showing relative levels of CLDN1, OCLN and TACSTD2 mRNA in siControl- and siTACSTD2-treated primary human hepatocytes. Data are expressed as 2-ΔΔCT, where ΔΔCT is the average difference between the siTACSTD2 ΔCT and siControl ΔCT. No significant difference was observed in CLDN1 and OCLN levels following siTACSTD2 treatment. (B) Western blot analysis showing no difference in CLDN1 and OCLN protein levels following gene silencing of TACSTD2 in parental and TACSTD2-overexpressing Huh7.5 cells. Tubulin was used as a loading control.
(PDF)

S14 Fig Effect of HCV infection on TACSTD2 expression. Quantitative RT-PCR data showing relative levels of TACSTD2 and HCV replication during the course of HCV infection at time 0, 24 and 48 hours after infection. Data are expressed as 2-ΔΔCT, where ΔΔCT is the average difference between each time point and the baseline (time 0).
(PDF)

S1 Table Genes differentially expressed in HCV-associated HCC tumor tissue as compared to the surrounding nontumorous tissue by t-test with a FDR <1% with 80% confidence level. Genes are sorted by the fold change (FC).
(DOCX)
