# Supplementary material for: Infection with hepatitis C virus depends on TACSTD2, a regulator of claudin-1 and occludin highly downregulated in hepatocellular carcinoma
Source: PLoS Pathog. 2018 Mar 14;14(3):e1006916. doi: 10.1371/journal.ppat.1006916 (PMC5882150; doi:10.1371/journal.ppat.1006916)
Supplement: S1 Fig — (A) Top-scored pathways, and (B) diseases and bio functions identified by IPA database (Ingenuity Pathway Analysis, http://www.ingenuity.com/) from the set of genes differentially expressed in HCV-associated HCC. Columns display the P-value calculated by Fisher’s exact test (left axis). Dots indicate the percentage of downregulated genes (right axis). All categories show a striking majority (on average 90%) of downregulated genes. (PDF) [file ppat.1006916.s001.pdf]

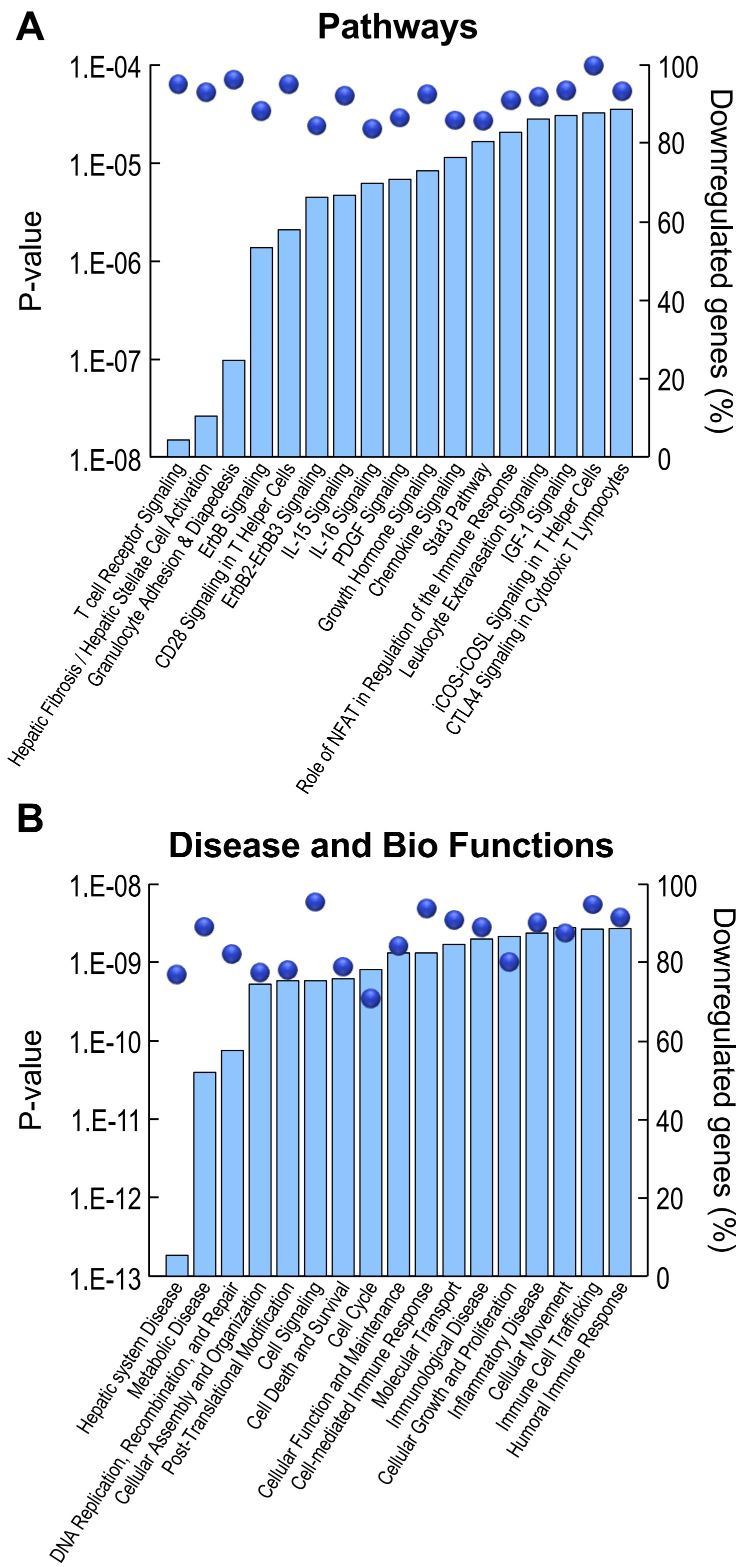

S1 Fig. Functional analysis of genes differentially expressed in HCC-associated HCV. Top-scored pathways (A), and diseases and bio functions (B) identified by IPA database (Ingenuity Pathway Analysis, http://www.ingenuity.com/) from the set of genes differentially expressed in HCV-associated HCC. Columns display the P-values calculated by Fisher's exact test (left axis). Dots indicate the percentage of downregulated genes (right axis). All categories show a striking majority (on average 90%) of downregulated genes.
