# Supplementary material for: Infection with hepatitis C virus depends on TACSTD2, a regulator of claudin-1 and occludin highly downregulated in hepatocellular carcinoma
Source: PLoS Pathog. 2018 Mar 14;14(3):e1006916. doi: 10.1371/journal.ppat.1006916 (PMC5882150; doi:10.1371/journal.ppat.1006916)
Supplement: S2 Fig — Gene expression levels were log2-transformed and row-wise standardized. Up-regulated genes are shown in shades of red; downregulated genes in shades of green. The dynamic range of colors is ± 1 SD. (PDF) [file ppat.1006916.s002.pdf]

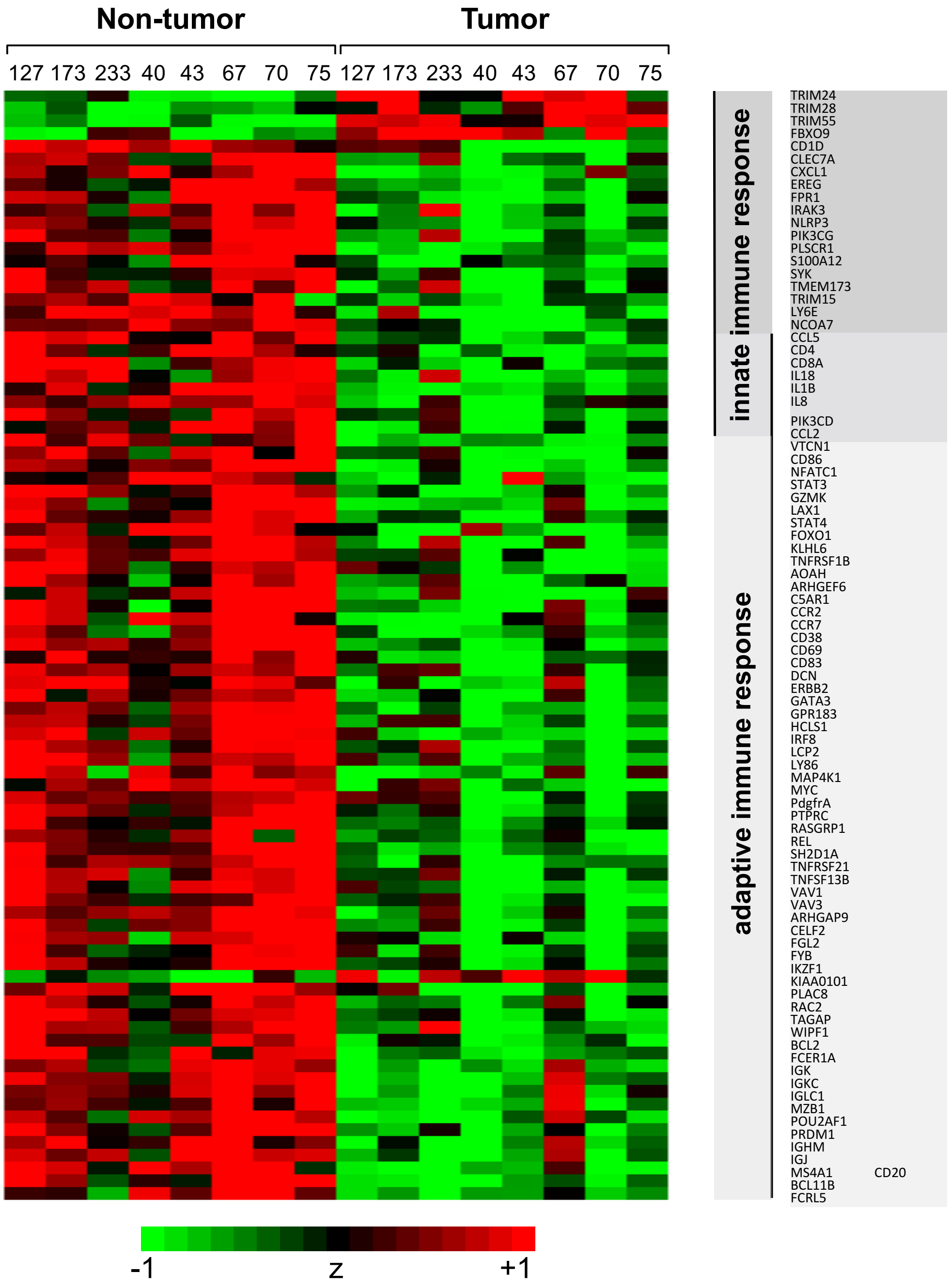

S2 Fig. Heatmap of 87 immune genes differentially expressed in liver samples from tumor and nontumorous tissues of 8 patients with HCV-associated HCC. Gene expression levels were log2-transformed and row-wise standardized. Up-regulated genes are shown in shades of red; downregulated genes in shades of green. The dynamic range of colors is ± 1 SD.
