# Supplementary material for: Infection with hepatitis C virus depends on TACSTD2, a regulator of claudin-1 and occludin highly downregulated in hepatocellular carcinoma
Source: PLoS Pathog. 2018 Mar 14;14(3):e1006916. doi: 10.1371/journal.ppat.1006916 (PMC5882150; doi:10.1371/journal.ppat.1006916)
Supplement: S3 Fig — Statistical significance (FDR 5%) was assessed by multivariate permutations. None of the host factors analyzed was differentially expressed, with the exception of TACSTD2, which was significantly downregulated within the tumor (P<0.00001). (PDF) [file ppat.1006916.s003.pdf]

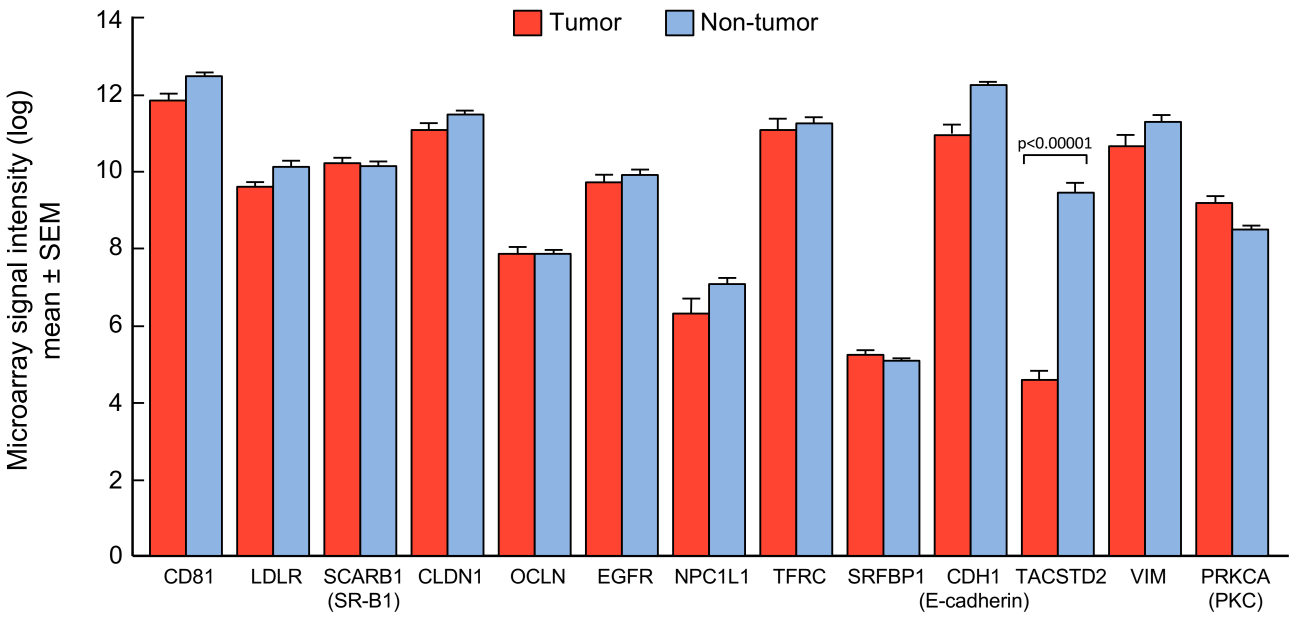

S3 Fig. Expression levels of known host factors involved in regulating HCV entry in liver samples from tumor (red bars) and nontumorous tissues (blue bars) of 8 patients with HCV-associated HCC. Statistical significance (FDR 5%) was assessed by multivariate permutations. None of the host factors analyzed was differentially expressed, with the exception of TACSTD2, which was significantly downregulated within the tumor ($P<0.00001$).
