# Supplementary material for: Infection with hepatitis C virus depends on TACSTD2, a regulator of claudin-1 and occludin highly downregulated in hepatocellular carcinoma
Source: PLoS Pathog. 2018 Mar 14;14(3):e1006916. doi: 10.1371/journal.ppat.1006916 (PMC5882150; doi:10.1371/journal.ppat.1006916)
Supplement: S4 Fig — To enhance the structural features, SR-B1 and CD81 are shown as red surface rendering, while nuclei are shown as blue volume rendering. No significant difference was observed in the distribution of SR-B1 and CD81 between the tumor and nontumorous tissue. (PDF) [file ppat.1006916.s004.pdf]

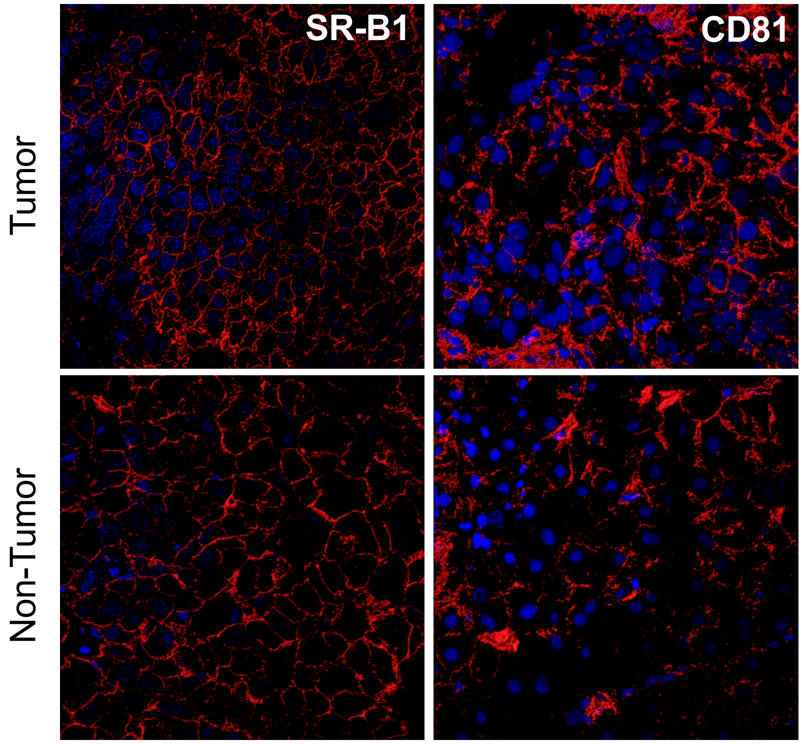

S4 Fig. Visualization of SRB1 and CD81 distribution in tumor and nontumorous tissue of a representative patient by immunofluorescence staining. To enhance the structural features, SR-B1 and CD81 are shown as red surface rendering, while nuclei are shown as blue volume rendering. No significant difference was observed in the distribution of SR-B1 and CD81 between the tumor and nontumorous tissue.
