# Supplementary material for: Infection with hepatitis C virus depends on TACSTD2, a regulator of claudin-1 and occludin highly downregulated in hepatocellular carcinoma
Source: PLoS Pathog. 2018 Mar 14;14(3):e1006916. doi: 10.1371/journal.ppat.1006916 (PMC5882150; doi:10.1371/journal.ppat.1006916)
Supplement: S5 Fig — (A) No correlation was found between SEC14L2 gene expression and HCV replication levels in tumorand nontumorous tissue. (B) A significant correlation was found between TACSTD2 gene expression and HCV replication levels in tumor and nontumorous tissue. (PDF) [file ppat.1006916.s005.pdf]

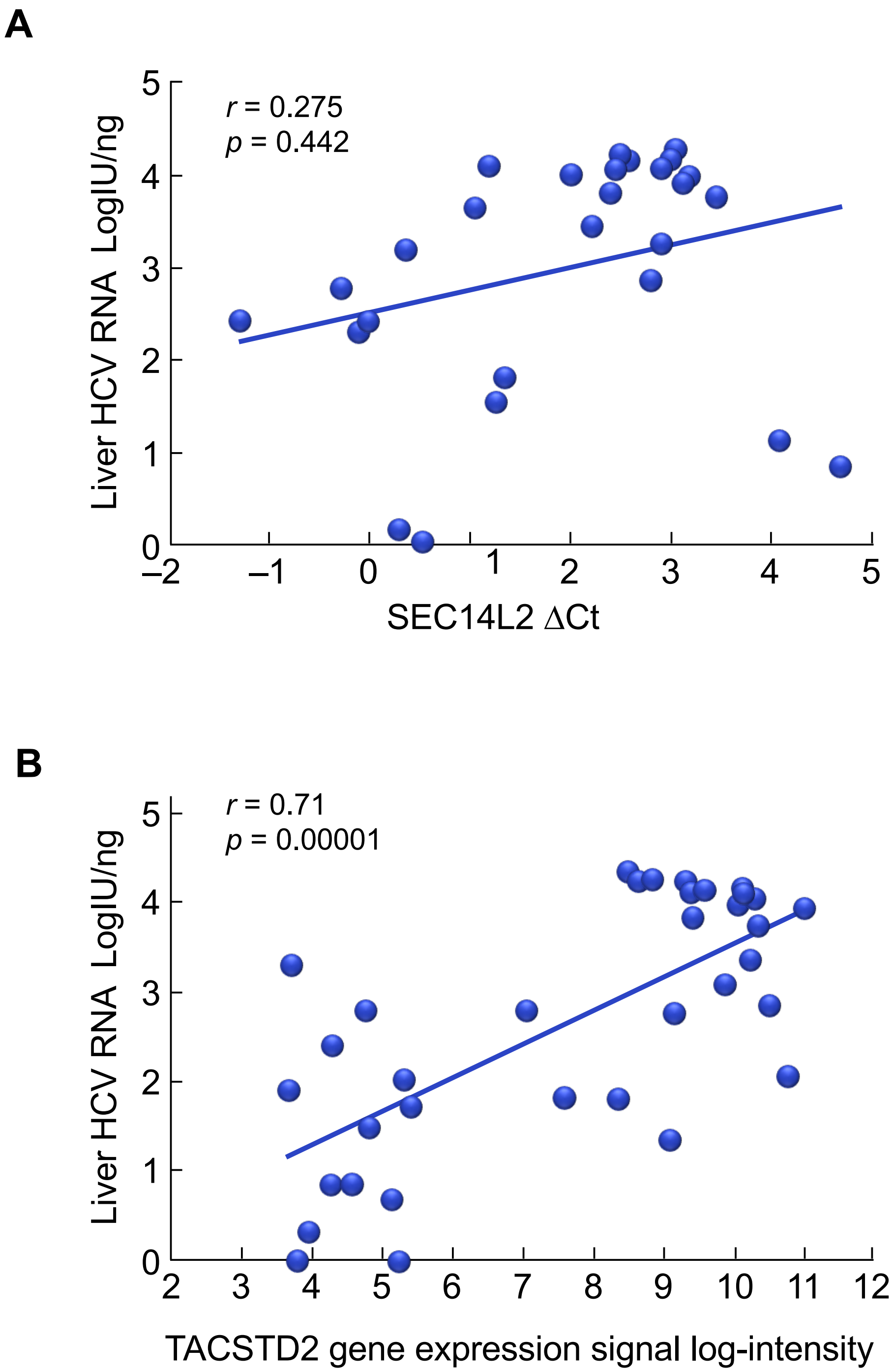

S5 Fig. SEC14L2 expression, TACSTD2 expression, and HCV RNA concentration. (A) No correlation was found between SEC14L2 gene expression and HCV replication levels in tumor and nontumorous tissue. (B) A significant correlation was found between TACSTD2 gene expression and HCV replication levels in tumor and nontumorous tissue.
