# Supplementary material for: Infection with hepatitis C virus depends on TACSTD2, a regulator of claudin-1 and occludin highly downregulated in hepatocellular carcinoma
Source: PLoS Pathog. 2018 Mar 14;14(3):e1006916. doi: 10.1371/journal.ppat.1006916 (PMC5882150; doi:10.1371/journal.ppat.1006916)
Supplement: S6 Fig — In TACSTD2- overexpressing cells both proteins are co-localized along the cellular membrane. (PDF) [file ppat.1006916.s006.pdf]

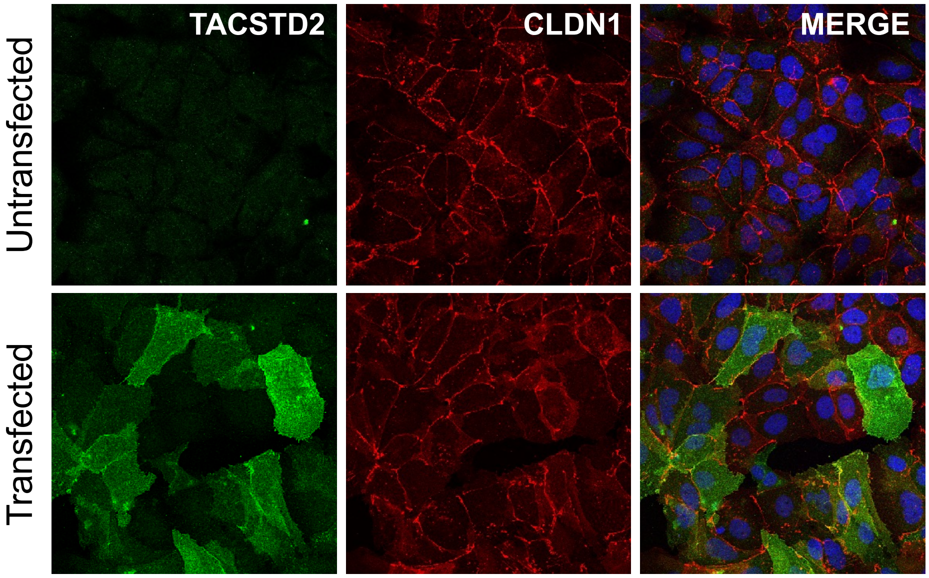

S6 Fig. Localization of TACSTD2 (green) and CLDN1 (red) in untransfected parental and TACSTD2-overexpressing Huh7.5 cells by immunofluorescence staining. In TACSTD2-overexpressing cells both proteins are co-localized along the cellular membrane.
