# Supplementary material for: Infection with hepatitis C virus depends on TACSTD2, a regulator of claudin-1 and occludin highly downregulated in hepatocellular carcinoma
Source: PLoS Pathog. 2018 Mar 14;14(3):e1006916. doi: 10.1371/journal.ppat.1006916 (PMC5882150; doi:10.1371/journal.ppat.1006916)
Supplement: S7 Fig — Cell lysates from parental and TACSTD2-overexpressing Huh7.5 cells were immunoprecipitated with anti- TACSTD2 antibody or control IgG, deglycosylated, and probed with anti-TACSTD2 or anti-Rb antibodies. No interaction was detected between TACSTD2 and Rb in the immunoprecipitated complex, although the proteins could be detected in the unbound fraction in the supernatant following immunoprecipitation. WCL denotes whole cellular lysate. (PDF) [file ppat.1006916.s007.pdf]

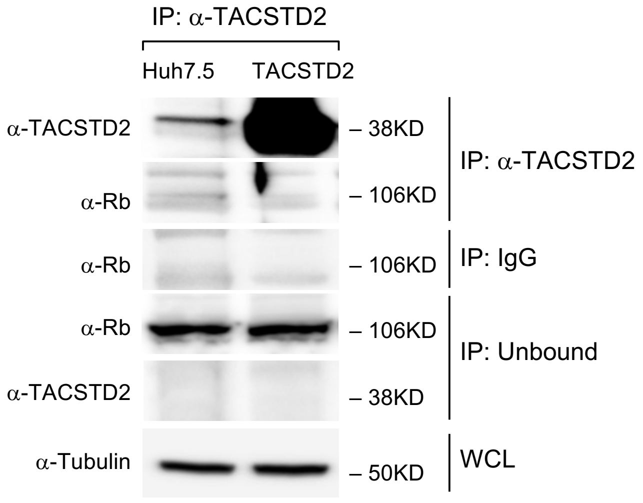

S7 Fig. Lack of interaction of TACSTD2 with the retinoblastoma (Rb) protein. Cell lysates from parental and TACSTD2-overexpressing Huh7.5 cells were immunoprecipitated with anti-TACSTD2 antibody or control IgG, deglycosylated, and probed with anti-TACSTD2 or anti-Rb antibodies. No interaction was detected between TACSTD2 and Rb in the immunoprecipitated complex, although the proteins could be detected in the unbound fraction in the supernatant following immunoprecipitation. WCL denotes whole cellular lysate.
