# Supplementary material for: Infection with hepatitis C virus depends on TACSTD2, a regulator of claudin-1 and occludin highly downregulated in hepatocellular carcinoma
Source: PLoS Pathog. 2018 Mar 14;14(3):e1006916. doi: 10.1371/journal.ppat.1006916 (PMC5882150; doi:10.1371/journal.ppat.1006916)
Supplement: S8 Fig — (A) Visualization of TACSTD2 (green) and CLDN1 (red) in TACSTD2-overexpressing Huh7.5 cells transfected with either siControl or siTACSTD2. CLDN1 (red) appears speckled and fragmented in siTACSTD2-treated cells, which show a complete loss of TACSTD2 staining, in contrast to the regular CLDN1 linear pattern observed in siControl-treated cells. (B) Visualization of TACSTD2 (green) and OCLN (red) in parental Huh7.5 cells transfected with siControl or siTACSTD2. OCLN (red) appears speckled and fragmented in siTACSTD2-treated cells, which show a complete loss of TACSTD2 staining, in contrast to the linear pattern observed in siControl-treated cells. (C) Parental Huh7.5 cells were transfected with siTACSTD2 or siControl and labeled with the division-tracking vital dye CFSE. The proportion of cells that underwent more than two replication cycles at 24, 48 and 72 hours was recorded by flow cytometry. Data represent the mean ± SEM of duplicate wells. No significant difference in proliferation was observed between cells treated with siTACSTD2 orsiControl. (D) Huh7.5 cells (Huh7.5) or TACSTD2-overexpressing cells (Huh7.5 TACSTD2) were labeled with the division-tracking vital dye CFSE. The proportion of cells that underwent more than two replication cycles at 24, 48 and 72 hours was recorded by flow cytometry. Data represent the mean ± SEM of duplicate wells. No significant difference in proliferation was observed between the two cell lines. (PDF) [file ppat.1006916.s008.pdf]

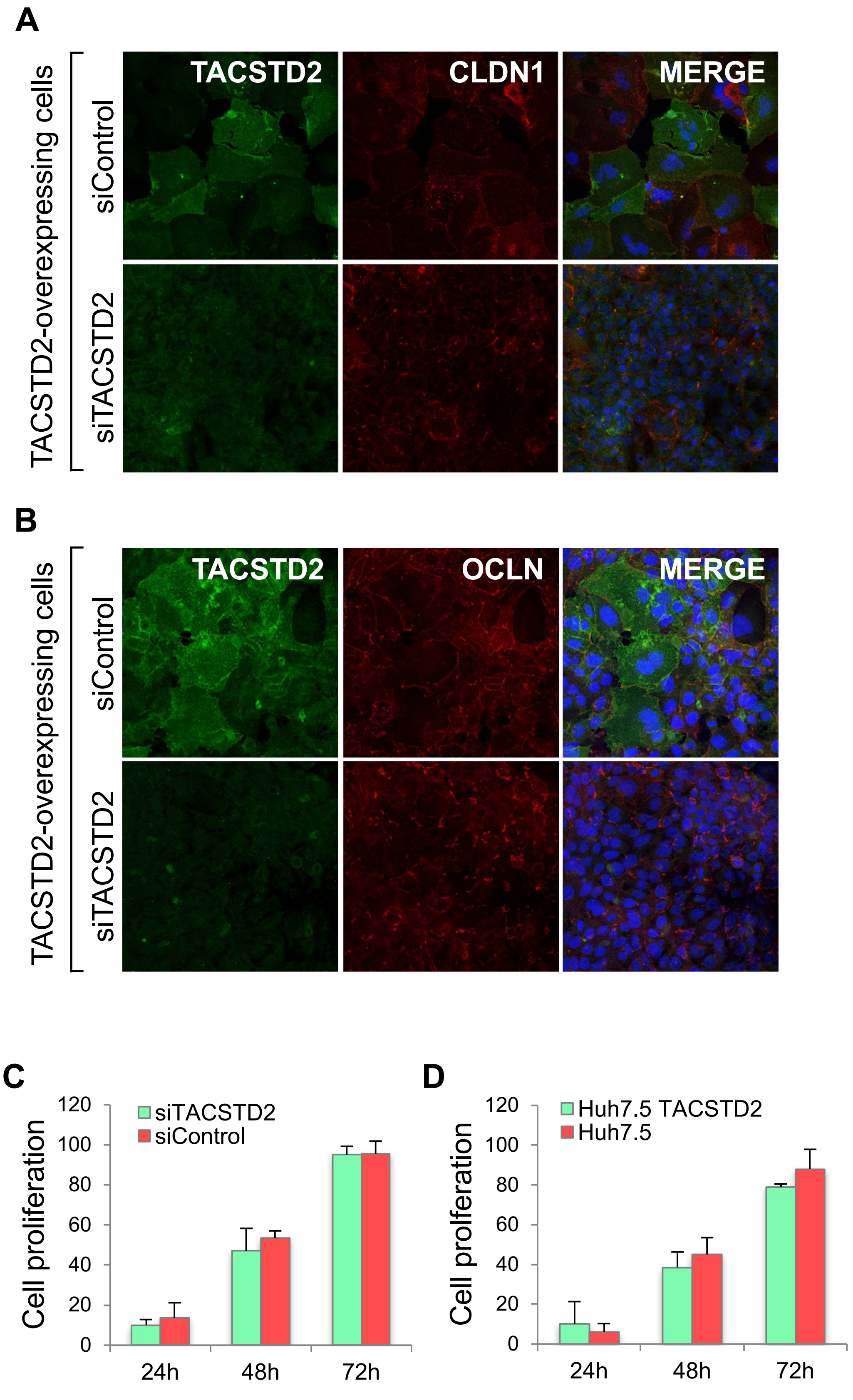
A
TACSTD2-overexpressing cells
siControl
siTACSTD2
TACSTD2
CLDN1
MERGE
B
TACSTD2-overexpressing cells
siControl
siTACSTD2
TACSTD2
OCLN
MERGE
C
Cell proliferation
0
20
40
60
80
100
120
siTACSTD2
siControl
24h
48h
72h
D
Cell proliferation
0
20
40
60
80
100
120
Huh7.5 TACSTD2
Huh7.5
24h
48h
72h

S8 Fig. Effect of TACSTD2 gene silencing on CLDN1 and OCLN distribution in TACSTD2-overexpressing Huh7.5 cells. (A)Visualization of TACSTD2 (green) and CLDN1 (red) in TACSTD2-overexpressing Huh7.5 cells transfected with either siControl or siTACSTD2. CLDN1 (red) appears speckled and fragmented in siTACSTD2-treated cells, which show a complete loss of TACSTD2 staining, in contrast to the regular CLDN1 linear pattern observed in siControl-treated cells. (B) Visualization of TACSTD2 (green) and OCLN (red) in parental Huh7.5 cells transfected with siControl or siTACSTD2. OCLN (red) appears speckled and fragmented in siTACSTD2-treated cells, which show a complete loss of TACSTD2 staining, in contrast to the linear pattern observed in siControl-treated cells. (C) Parental Huh7.5 cells were transfected with siTACSTD2 or siControl and labeled with the division-tracking vital dye CFSE. The proportion of cells that underwent more than two replication cycles at 24, 48 and 72 hours was recorded by flow cytometry. Data represent the mean ± SE of duplicate wells. No significant difference in proliferation was observed between cells treated with siTACSTD2 or siControl. (D) Huh7.5 cells (Huh7.5) or TACSTD2-overexpressing cells (Huh7.5 TACSTD2) were labeled with the division-tracking vital dye CFSE. The proportion of cells that underwent more than two replication cycles at 24, 48 and 72 hours was recorded by flow cytometry. Data represent the mean ± SE of duplicate wells. No significant difference in proliferation was observed between the two cell lines.
