# Supplementary material for: Infection with hepatitis C virus depends on TACSTD2, a regulator of claudin-1 and occludin highly downregulated in hepatocellular carcinoma
Source: PLoS Pathog. 2018 Mar 14;14(3):e1006916. doi: 10.1371/journal.ppat.1006916 (PMC5882150; doi:10.1371/journal.ppat.1006916)
Supplement: S9 Fig — (A) Immunoprecipitation with an anti-TACSTD2 antibody following TACSTD2 gene silencing showed a visible TACSTD2 band in Huh7.5 cells transfected with siControl but not with siTACSTD2. (B) Visualization of CLDN1, OCLN, and ZO-1 in parental Huh7.5 cells transfected with either siControl or siTACSTD2. All three tight junction proteins appear disrupted in siTACSTD2-treated cells in contrast to their regular linear pattern observed in siControl-treated cells. (C) Quantitative RT-PCR data showing relative levels of TACSTD2 mRNA in siControl- and siTACSTD2-transfected parental Huh 7.5 cells. Data are expressed as 2-ΔΔCT, where ΔΔCT is the average difference between the siTACSTD2 ΔCT and siControl ΔCT. (PDF) [file ppat.1006916.s009.pdf]

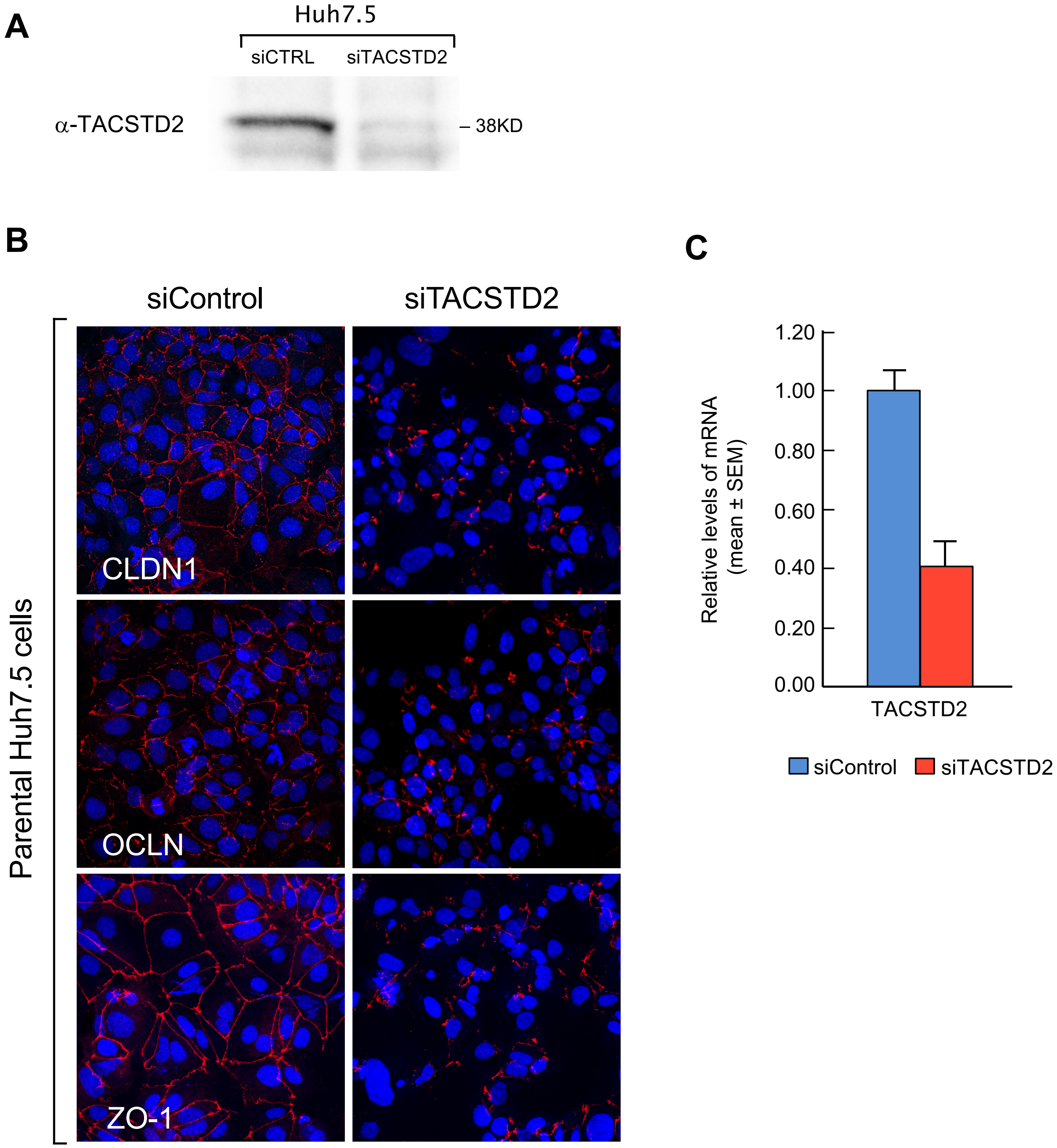

S9 Fig. TACSTD2 gene silencing in parental Huh 7.5 cells and its effect on tight junction protein distribution. (A) Immunoprecipitation with an anti-TACSTD2 antibody following TACSTD2 gene silencing showed a visible TACSTD2 band in Huh7.5 cells transfected with siControl but not with siTACSTD2. (B) Visualization of CLDN1, OCLN, and ZO-1 in parental Huh7.5 cells transfected with either siControl or siTACSTD2. All three tight junction proteins appear disrupted in siTACSTD2-treated cells in contrast to their regular linear pattern observed in siControl-treated cells. (C) Quantitative RT-PCR data showing relative levels of TACSTD2 mRNA in siControl- and siTACSTD2-transfected parental Huh 7.5 cells. Data are expressed as $2^{-\Delta\Delta CT}$, where $\Delta\Delta C_T$ is the average difference between the siTACSTD2 ΔC_T and siControl ΔC_T.
