# Supplementary material for: Infection with hepatitis C virus depends on TACSTD2, a regulator of claudin-1 and occludin highly downregulated in hepatocellular carcinoma
Source: PLoS Pathog. 2018 Mar 14;14(3):e1006916. doi: 10.1371/journal.ppat.1006916 (PMC5882150; doi:10.1371/journal.ppat.1006916)
Supplement: S10 Fig — (A) Visualization of ZO-1 (red) in parental Huh7.5 cells transfected with either siControl or siTACSTD2. ZO-1 appears disrupted in siTACSTD2-treated cells in contrast to the regular ZO-1 linear pattern observed in siControl-treated cells. (B) Visualization of JAM-A (red) in parental Huh7.5 cells transfected with siControl or siTACSTD2. JAM-A (red) appears disrupted in siTACSTD2-treated cells in contrast to the linear pattern observed in siControltreated cells. (PDF) [file ppat.1006916.s010.pdf]

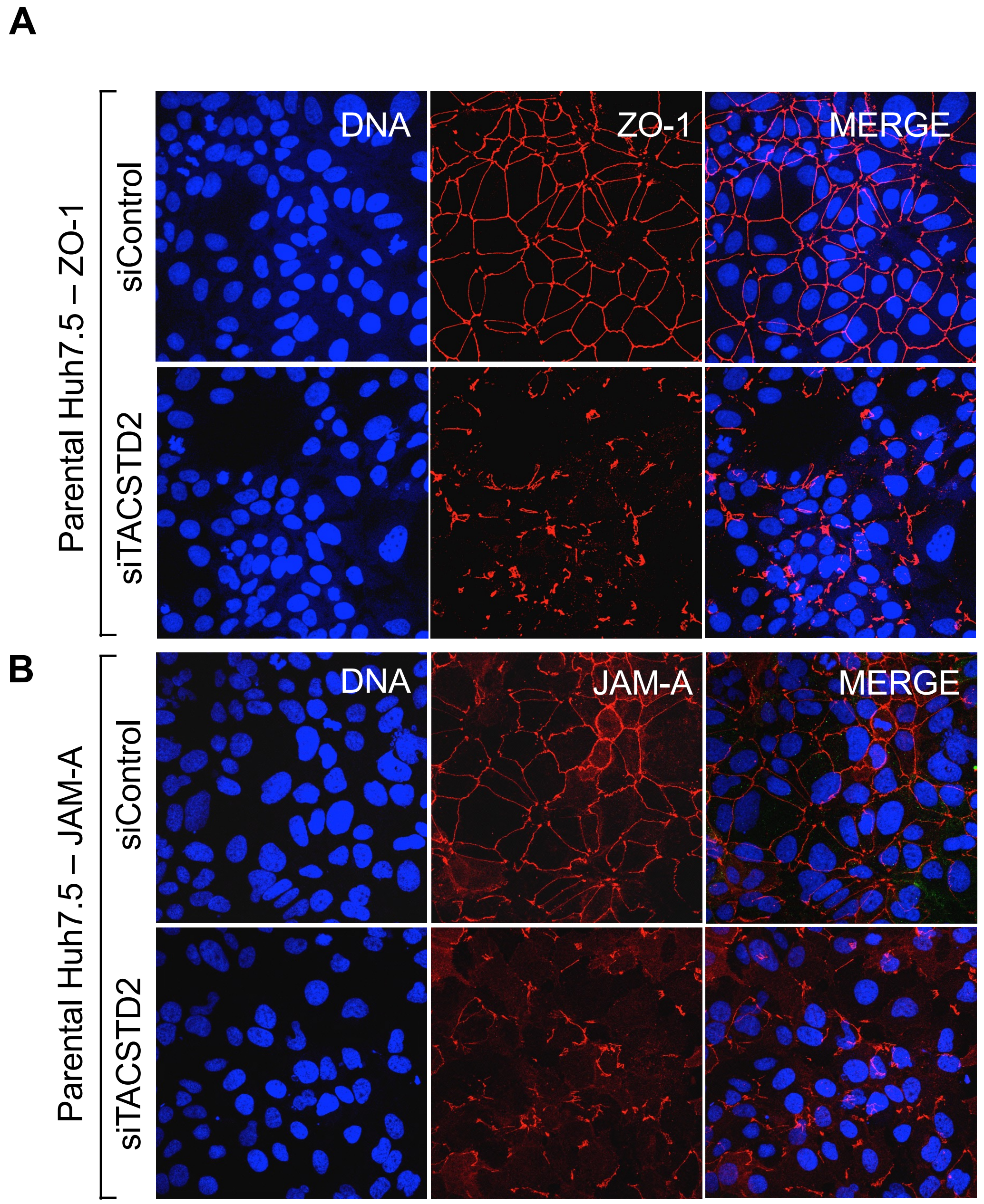

S10 Fig. Effect of TACSTD2 gene silencing on ZO-1 and JAM-A distribution in parental Huh7.5 cells. (A) Visualization of ZO-1 (red) in parental Huh7.5 cells transfected with either siControl or siTACSTD2. ZO-1 appears disrupted in siTACSTD2-treated cells in contrast to the regular ZO-1 linear pattern observed in siControl-treated cells. (B) Visualization of JAM-A (red) in parental Huh7.5 cells transfected with siControl or siTACSTD2. JAM-A (red) appears disrupted in siTACSTD2-treated cells in contrast to the linear pattern observed in siControl-treated cells.
