# Supplementary material for: Infection with hepatitis C virus depends on TACSTD2, a regulator of claudin-1 and occludin highly downregulated in hepatocellular carcinoma
Source: PLoS Pathog. 2018 Mar 14;14(3):e1006916. doi: 10.1371/journal.ppat.1006916 (PMC5882150; doi:10.1371/journal.ppat.1006916)
Supplement: S11 Fig — (A) Detection of TACSTD2 in parental Huh7.5 cells by immunoprecipitation with an anti-TACSTD2 antibody followed by deglycosylation. (B) TACSTD2 gene silencing did not affect the expression of E-cadherin in parental Huh7.5 cells. Cell lysates from parental Huh7.5 cells were analyzed by Western blot with anti-E-cadherin 72h after transfection with either siControl or siTACSTD2. (C) Visualization of E-cadherin (red) in parental Huh7.5 cells transfected with either siControl or siTACSTD2. The cellular localization of E-cadherin in siTACSTD2-transfected Huh7.5 cells was similar to that observed in siControltransfected cells. (PDF) [file ppat.1006916.s011.pdf]

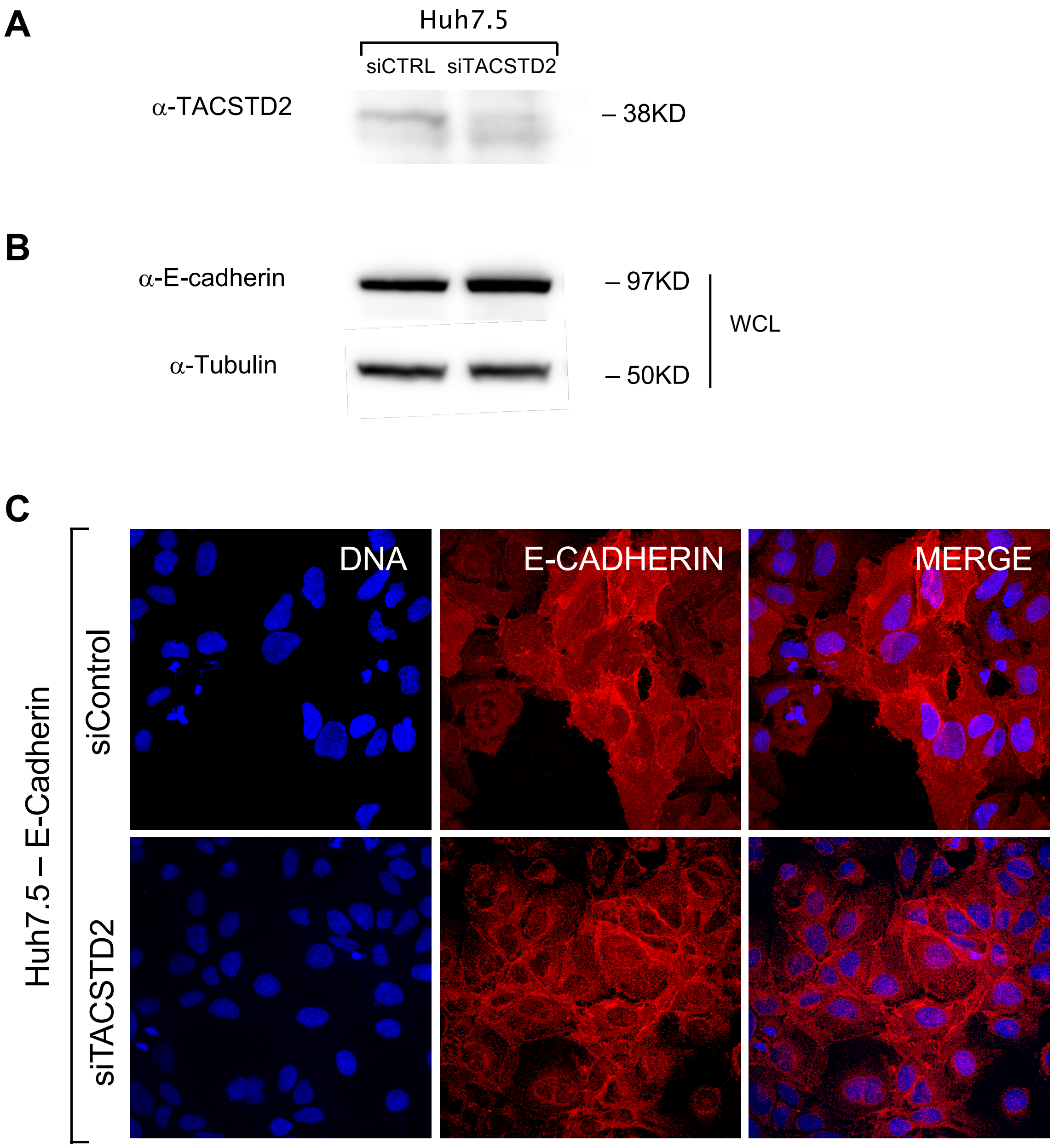

S11 Fig. Effect of TACSTD2 gene silencing on the expression and distribution of E-cadherin in parental Huh7.5 cells. (A) Detection of TACSTD2 in parental Huh7.5 cells by immunoprecipitation with an anti-TACSTD2 antibody followed by deglycosylation. (B) *TACSTD2* gene silencing did not affect the expression of E-cadherin in parental Huh7.5 cells. Cell lysates from parental Huh7.5 cells were analyzed by Western blot with anti-E-cadherin 72h after transfection with either siControl or siTACSTD2. (C) Visualization of E-cadherin (red) in parental Huh7.5 cells transfected with either siControl or siTACSTD2. The cellular localization of E-cadherin in siTACSTD2-transfected Huh7.5 cells was similar to that observed in siControl-transfected cells.
