# Supplementary material for: Infection with hepatitis C virus depends on TACSTD2, a regulator of claudin-1 and occludin highly downregulated in hepatocellular carcinoma
Source: PLoS Pathog. 2018 Mar 14;14(3):e1006916. doi: 10.1371/journal.ppat.1006916 (PMC5882150; doi:10.1371/journal.ppat.1006916)
Supplement: S12 Fig — Quantitative RT-PCR data showing relative levels of TACSTD2, E-cadherin and vimentin mRNA in siControl- and siTACSTD2-treated parental Huh 7.5 cells. Data are expressed as 2- ΔΔCT, where ΔΔCT is the average difference between the siTACSTD2 ΔCT and siControl ΔCT. An increase in E-cadherin and decrease in vimentin levels were observed following siTACSTD2 gene silencing. (PDF) [file ppat.1006916.s012.pdf]

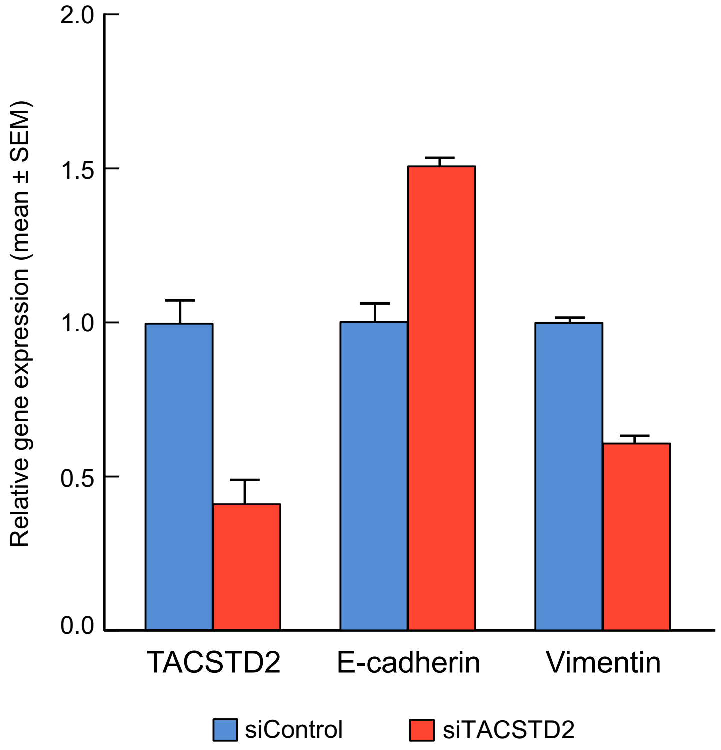

S12 Fig. Effect of TACSTD2 gene silencing on E-cadherin and vimentin mRNA expression. Quantitative RT-PCR data showing relative levels of TACSTD2, E-cadherin and vimentin mRNA in siControl- and siTACSTD2-treated parental Huh 7.5 cells. Data are expressed as $2^{-\Delta\Delta CT}$, where $\Delta\Delta C_T$ is the average difference between the siTACSTD2 ΔC_T and siControl ΔC_T. An increase in E-cadherin and decrease in vimentin levels were observed following siTACSTD2 gene silencing.
