# Supplementary material for: Infection with hepatitis C virus depends on TACSTD2, a regulator of claudin-1 and occludin highly downregulated in hepatocellular carcinoma
Source: PLoS Pathog. 2018 Mar 14;14(3):e1006916. doi: 10.1371/journal.ppat.1006916 (PMC5882150; doi:10.1371/journal.ppat.1006916)
Supplement: S13 Fig — (A) Quantitative RT-PCR data showing relative levels of CLDN1, OCLN and TACSTD2 mRNA in siControl- and siTACSTD2-treated primary human hepatocytes. Data are expressed as 2-ΔΔCT, where ΔΔCT is the average difference between the siTACSTD2 ΔCT and siControl ΔCT. No significant difference was observed in CLDN1 and OCLN levels following siTACSTD2 treatment. (B) Western blot analysis showing no difference in CLDN1 and OCLN protein levels following gene silencing of TACSTD2 in parental and TACSTD2-overexpressing Huh7.5 cells. Tubulin was used as a loading control. (PDF) [file ppat.1006916.s013.pdf]

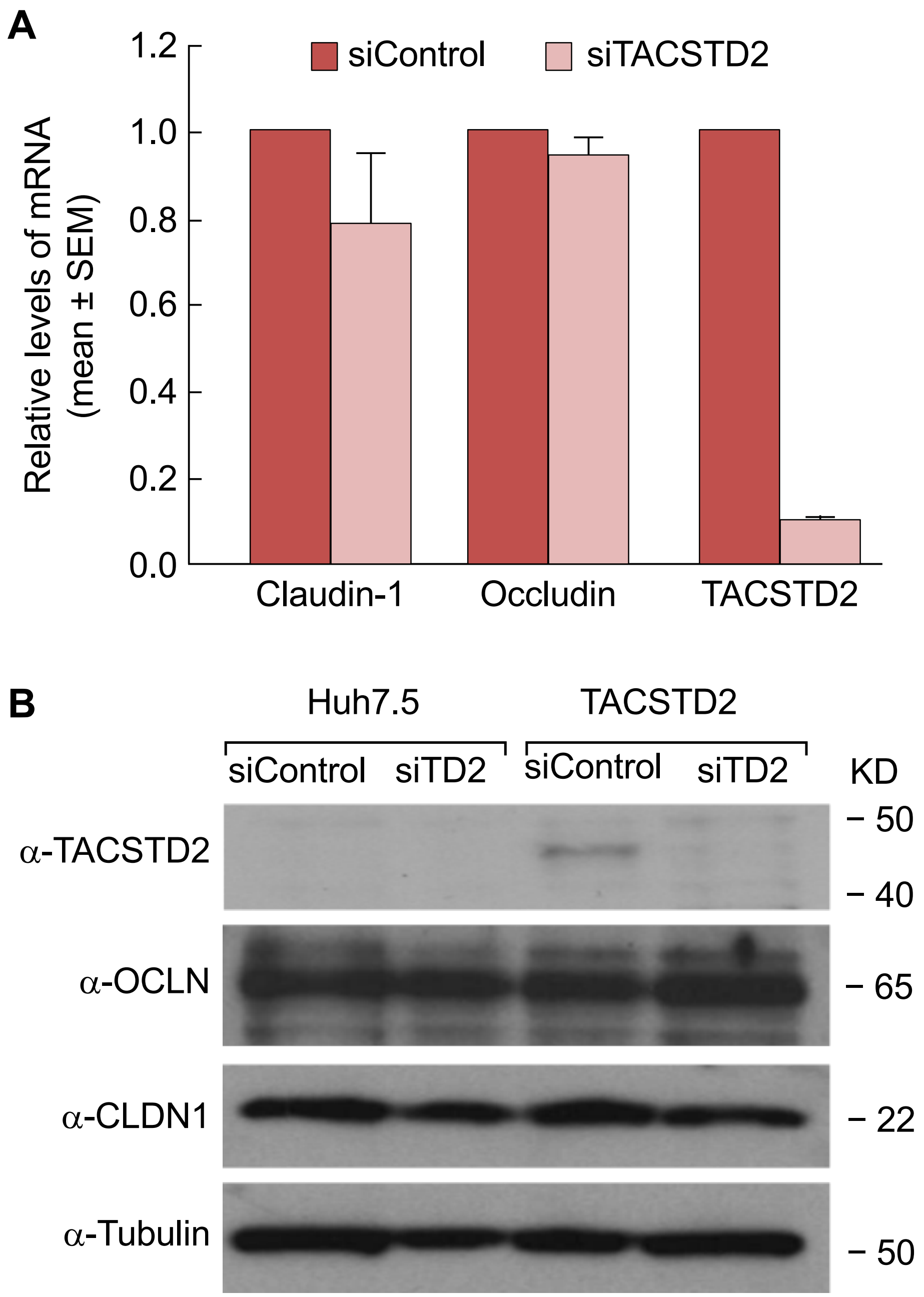

S13 Fig. Effect of TACSTD2 gene silencing on CLDN1 and OCLN mRNA and protein expression. (A) Quantitative RT-PCR data showing relative levels of CLDN1, OCLN and TACSTD2 mRNA in siControl- and siTACSTD2-treated primary human hepatocytes. Data are expressed as $2^{-\Delta\Delta CT}$, where $\Delta\Delta C_T$ is the average difference between the siTACSTD2 ΔC_T and siControl ΔC_T. No significant difference was observed in CLDN1 and OCLN levels following siTACSTD2 treatment. (B) Western blot analysis showing no difference in CLDN1 and OCLN protein levels following gene silencing of TACSTD2 in parental and TACSTD2-overexpressing Huh7.5 cells. Tubulin was used as a loading control.
