# Supplementary material for: Infection with hepatitis C virus depends on TACSTD2, a regulator of claudin-1 and occludin highly downregulated in hepatocellular carcinoma
Source: PLoS Pathog. 2018 Mar 14;14(3):e1006916. doi: 10.1371/journal.ppat.1006916 (PMC5882150; doi:10.1371/journal.ppat.1006916)
Supplement: S14 Fig — Quantitative RT-PCR data showing relative levels of TACSTD2 and HCV replication during the course of HCV infection at time 0, 24 and 48 hours after infection. Data are expressed as 2-ΔΔCT, where ΔΔCT is the average difference between each time point and the baseline (time 0). (PDF) [file ppat.1006916.s014.pdf]

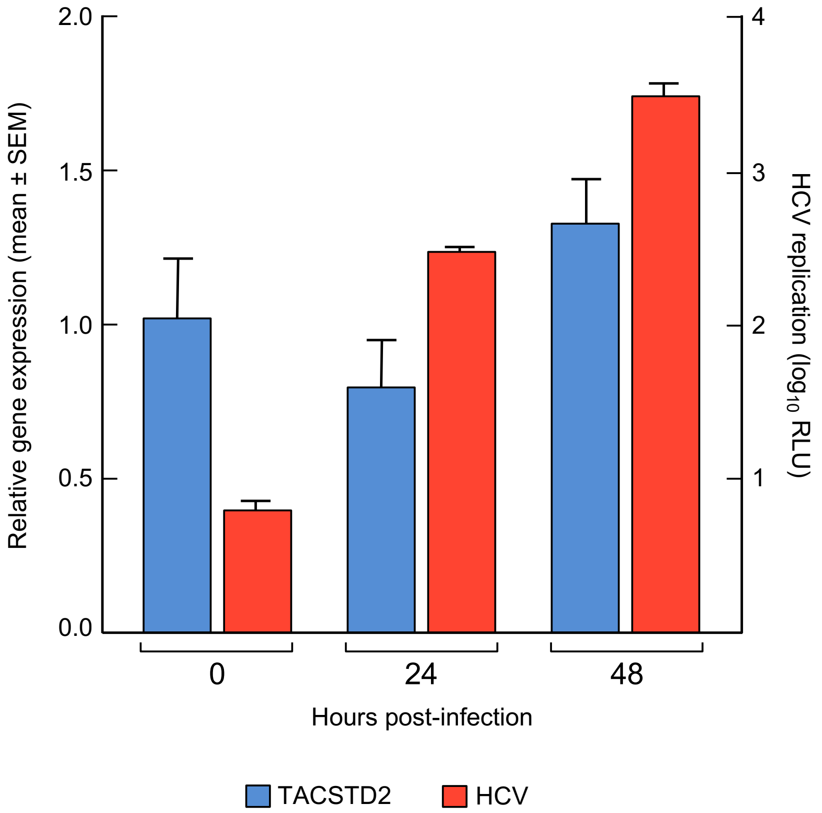

S14 Fig. Effect of HCV infection on TACSTD2 expression. Quantitative RT-PCR data showing relative levels of TACSTD2 and HCV replication during the course of HCV infection at time 0, 24 and 48 hours after infection. Data are expressed as $2^{-\Delta\Delta CT}$, where $\Delta\Delta C_T$ is the average difference between each time point and the baseline (time 0).
