# Supplementary material for: Infection with hepatitis C virus depends on TACSTD2, a regulator of claudin-1 and occludin highly downregulated in hepatocellular carcinoma
Source: PLoS Pathog. 2018 Mar 14;14(3):e1006916. doi: 10.1371/journal.ppat.1006916 (PMC5882150; doi:10.1371/journal.ppat.1006916)
Supplement: S1 Table — Genes are sorted by the fold change (FC). (DOCX) [file ppat.1006916.s015.docx]

**S1 Table. Genes differentially expressed in HCV-associated HCC tumor tissue as compared to the surrounding non-tumorous tissue by t-test with a FDR <1% with 80% confidence level.**

Genes are sorted by the fold change (FC).

**EntrezID Gene symbol FC Gene Title**

| 4072 | EPCAM | -68.5 | epithelial cell adhesion molecule |
| --- | --- | --- | --- |
| 4070 | TACSTD2 | -34.7 | tumor-associated calcium signal transducer 2 |
| 2354 | FOSB | -17.5 | FBJ murine osteosarcoma viral oncogene homolog B |
| 6372 | CXCL6 | -15.3 | chemokine (C-X-C motif) ligand 6 |
| 3773 | KCNJ16 | -14.9 | potassium inwardly-rectifying channel, subfamily J, member 16 |
| 1080 | CFTR | -13.3 | cystic fibrosis transmembrane conductance regulator (ATP-binding cassette sub-family C, member 7) |
| 10164 | CHST4 | -12.2 | carbohydrate (N-acetylglucosamine 6-O) sulfotransferase 4 |
| 8406 | SRPX | -11.6 | sushi-repeat containing protein, X-linked |
| 306 | ANXA3 | -11.6 | annexin A3 |
| 54102 | CLIC6 | -11.3 | chloride intracellular channel 6 |
| 3082 | HGF | -11.1 | hepatocyte growth factor (hepapoietin A; scatter factor) |
| 9547 | CXCL14 | -10.9 | chemokine (C-X-C motif) ligand 14 |
| 3696 | ITGB8 | -10.8 | integrin, beta 8 |
| 8842 | PROM1 | -10.5 | prominin 1 |
| 730 | C7 | -10.5 | complement component 7 |
| 5743 | PTGS2 | -10.2 | prostaglandin-endoperoxide synthase 2 (prostaglandin G/H synthase and cyclooxygenase) |
| 285016 | FAM150B | -10.2 | family with sequence similarity 150, member B |
| 341640 | FREM2 | -9.9 | FRAS1 related extracellular matrix protein 2 |
| 2823 | GPM6A | -9.6 | glycoprotein M6A |
| 9071 | CLDN10 | -9.6 | claudin 10 |
| 3400 | ID4 | -9.3 | inhibitor of DNA binding 4, dominant negative helix-loop-helix protein |
| 2568 | GABRP | -9.3 | gamma-aminobutyric acid (GABA) A receptor, pi |
| 7057 | THBS1 | -9.2 | thrombospondin 1 |
| 3512 | IGJ | -9.1 | immunoglobulin J polypeptide, linker protein for immunoglobulin alpha and mu polypeptides |
| 5156 | PDGFRA | -9.1 | platelet-derived growth factor receptor, alpha polypeptide |
| 9021 | SOCS3 | -8.9 | suppressor of cytokine signaling 3 |
| 2263 | FGFR2 | -8.8 | fibroblast growth factor receptor 2 |
| 57817 | HAMP | -8.8 | hepcidin antimicrobial peptide |
| 5999 | RGS4 | -8.7 | regulator of G-protein signaling 4 |
| 7980 | TFPI2 | -8.4 | tissue factor pathway inhibitor 2 |
| 79689 | STEAP4 | -8.3 | STEAP family member 4 |
| 27165 | GLS2 | -8.3 | glutaminase 2 (liver, mitochondrial) |
| 4499 | MT1M | -8.1 | metallothionein 1M |
| 387763 | C11orf96 | -8.0 | chromosome 11 open reading frame 96 |
| 3576 | IL8 | -8.0 | interleukin 8 |
| 6387 | CXCL12 | -7.9 | chemokine (C-X-C motif) ligand 12 |
| 2619 | GAS1 | -7.9 | growth arrest-specific 1 |
| 57393 | TMEM27 | -7.7 | transmembrane protein 27 |
| 3084 | NRG1 | -7.5 | neuregulin 1 |
| 83716 | CRISPLD2 | -7.5 | cysteine-rich secretory protein LCCL domain containing 2 |
| 2353 | FOS | -7.5 | FBJ murine osteosarcoma viral oncogene homolog |
| 1634 | DCN | -7.5 | decorin |
| 139221 | MUM1L1 | -7.4 | melanoma associated antigen (mutated) 1-like 1 |
| 3537 | IGLC1 | -7.3 | immunoglobulin lambda constant 1 (Mcg marker) |
| 80144 | FRAS1 | -7.2 | Fraser syndrome 1 |
| 288 | ANK3 | -7.2 | ankyrin 3, node of Ranvier (ankyrin G) |
| 1544 | CYP1A2 | -7.0 | cytochrome P450, family 1, subfamily A, polypeptide 2 |
| 170392 | OIT3 | -6.9 | oncoprotein induced transcript 3 |
| 2220 | FCN2 | -6.7 | ficolin (collagen/fibrinogen domain containing lectin) 2 (hucolin) |
| 3977 | LIFR | -6.7 | leukemia inhibitory factor receptor alpha |
| 147372 | CCBE1 | -6.6 | collagen and calcium binding EGF domains 1 |
| 2042 | EPHA3 | -6.6 | EPH receptor A3 |
| 9510 | ADAMTS1 | -6.6 | ADAM metallopeptidase with thrombospondin type 1 motif, 1 |
| 6694 | SPP2 | -6.6 | secreted phosphoprotein 2, 24kDa |
| 3855 | KRT7 | -6.6 | keratin 7 |
| 6519 | SLC3A1 | -6.5 | solute carrier family 3 (amino acid transporter heavy chain), member 1 |
| 1556 | CYP2B7P1 | -6.5 | cytochrome P450, family 2, subfamily B, polypeptide 7 pseudogene 1 |
| 5997 | RGS2 | -6.5 | regulator of G-protein signaling 2, 24kDa |
| 28513 | CDH19 | -6.4 | cadherin 19, type 2 |
| 2562 | GABRB3 | -6.4 | gamma-aminobutyric acid (GABA) A receptor, beta 3 |
| 3880 | KRT19 | -6.3 | keratin 19 |
| 5178 | PEG3 | -6.3 | paternally expressed 3 |
| 64399 | HHIP | -6.2 | hedgehog interacting protein |
| 26751 | SH3YL1 | -6.1 | SH3 domain containing, Ysc84-like 1 (S. cerevisiae) |
| 259232 | NALCN | -6.0 | sodium leak channel, non-selective |
| 5450 | POU2AF1 | -5.9 | POU class 2 associating factor 1 |
| 54346 | UNC93A | -5.8 | unc-93 homolog A (C. elegans) |
| 78989 | COLEC11 | -5.8 | collectin sub-family member 11 |
| 10653 | SPINT2 | -5.7 | serine peptidase inhibitor, Kunitz type, 2 |
| 969 | CD69 | -5.7 | CD69 molecule |
| 84870 | RSPO3 | -5.7 | R-spondin 3 |
| 486 | FXYD2 | -5.6 | FXYD domain containing ion transport regulator 2 |
| 6363 | CCL19 | -5.6 | chemokine (C-C motif) ligand 19 |
| 25890 | ABI3BP | -5.6 | ABI family, member 3 (NESH) binding protein |
| 3575 | IL7R | -5.5 | interleukin 7 receptor |
| 27303 | RBMS3 | -5.5 | RNA binding motif, single stranded interacting protein 3 |
| 3507 | IGHM | -5.5 | immunoglobulin heavy constant mu |
| 4609 | MYC | -5.5 | v-myc avian myelocytomatosis viral oncogene homolog |
| 84879 | MFSD2A | -5.5 | major facilitator superfamily domain containing 2A |
| 256764 | WDR72 | -5.4 | WD repeat domain 72 |
| 201799 | TMEM154 | -5.4 | transmembrane protein 154 |
| 55553 | SOX6 | -5.4 | SRY (sex determining region Y)-box 6 |
| 51302 | CYP39A1 | -5.3 | cytochrome P450, family 39, subfamily A, polypeptide 1 |
| 8013 | NR4A3 | -5.3 | nuclear receptor subfamily 4, group A, member 3 |
| 11185 | INMT | -5.2 | indolethylamine N-methyltransferase |
| 4886 | NPY1R | -5.2 | neuropeptide Y receptor Y1 |
| 358 | AQP1 | -5.2 | aquaporin 1 |
| 7039 | TGFA | -5.1 | transforming growth factor, alpha |
| 8547 | FCN3 | -5.1 | ficolin (collagen/fibrinogen domain containing) 3 (Hakata antigen) |
| 79966 | SCD5 | -5.1 | stearoyl-CoA desaturase 5 |
| 339390 | CLEC4G | -5.0 | C-type lectin domain family 4, member G |
| 3514 | IGKC | -5.0 | immunoglobulin kappa constant |
| 25984 | KRT23 | -5.0 | keratin 23 (histone deacetylase inducible) |
| 9314 | KLF4 | -5.0 | Kruppel-like factor 4 (gut) |
| 56944 | OLFML3 | -4.9 | olfactomedin-like 3 |
| 5314 | PKHD1 | -4.9 | polycystic kidney and hepatic disease 1 (autosomal recessive) |
| 6332 | SCN7A | -4.9 | sodium channel, voltage-gated, type VII, alpha subunit |
| 54845 | ESRP1 | -4.9 | epithelial splicing regulatory protein 1 |
| 2152 | F3 | -4.9 | coagulation factor III (thromboplastin, tissue factor) |
| 23414 | ZFPM2 | -4.9 | zinc finger protein, FOG family member 2 |
| 4316 | MMP7 | -4.8 | matrix metallopeptidase 7 (matrilysin, uterine) |
| 3486 | IGFBP3 | -4.8 | insulin-like growth factor binding protein 3 |
| 5947 | RBP1 | -4.8 | retinol binding protein 1, cellular |
| 56937 | PMEPA1 | -4.7 | prostate transmembrane protein, androgen induced 1 |
| 2104 | ESRRG | -4.7 | estrogen-related receptor gamma |
| 84873 | GPR128 | -4.7 | G protein-coupled receptor 128 |
| 760 | CA2 | -4.7 | carbonic anhydrase II |
| 7852 | CXCR4 | -4.6 | chemokine (C-X-C motif) receptor 4 |
| 5740 | PTGIS | -4.6 | prostaglandin I2 (prostacyclin) synthase |
| 1393 | CRHBP | -4.6 | corticotropin releasing hormone binding protein |
| 931 | MS4A1 | -4.6 | membrane-spanning 4-domains, subfamily A, member 1 |
| 6515 | SLC2A3 | -4.6 | solute carrier family 2 (facilitated glucose transporter), member 3 |
| 3680 | ITGA9 | -4.6 | integrin, alpha 9 |
| 151531 | UPP2 | -4.6 | uridine phosphorylase 2 |
| 1602 | DACH1 | -4.5 | dachshund homolog 1 (Drosophila) |
| 4651 | MYO10 | -4.5 | myosin X |
| 7056 | THBD | -4.5 | thrombomodulin |
| 6347 | CCL2 | -4.4 | chemokine (C-C motif) ligand 2 |
| 9068 | ANGPTL1 | -4.4 | angiopoietin-like 1 |
| 2591 | GALNT3 | -4.4 | UDP-N-acetyl-alpha-D-galactosamine:polypeptide N-acetylgalactosaminyltransferase 3 (GalNAc-T3) |
| 64097 | EPB41L4A | -4.3 | erythrocyte membrane protein band 4.1 like 4A |
| 92126 | DSEL | -4.3 | dermatan sulfate epimerase-like |
| 1910 | EDNRB | -4.3 | endothelin receptor type B |
| 287 | ANK2 | -4.3 | ankyrin 2, neuronal |
| 79679 | VTCN1 | -4.2 | V-set domain containing T cell activation inhibitor 1 |
| 55422 | ZNF331 | -4.2 | zinc finger protein 331 |
| 27242 | TNFRSF21 | -4.2 | tumor necrosis factor receptor superfamily, member 21 |
| 91353 | IGLL3P | -4.2 | immunoglobulin lambda-like polypeptide 3, pseudogene |
| 51316 | PLAC8 | -4.1 | placenta-specific 8 |
| 1285 | COL4A3 | -4.1 | collagen, type IV, alpha 3 (Goodpasture antigen) |
| 1805 | DPT | -4.1 | dermatopontin |
| 79901 | CYBRD1 | -4.1 | cytochrome b reductase 1 |
| 54463 | FAM134B | -4.1 | family with sequence similarity 134, member B |
| 140876 | FAM65C | -4.1 | family with sequence similarity 65, member C |
| 8870 | IER3 | -4.0 | immediate early response 3 |
| 80310 | PDGFD | -4.0 | platelet derived growth factor D |
| 1960 | EGR3 | -4.0 | early growth response 3 |
| 3397 | ID1 | -4.0 | inhibitor of DNA binding 1, dominant negative helix-loop-helix protein |
| 54704 | PDP1 | -4.0 | pyruvate dehyrogenase phosphatase catalytic subunit 1 |
| 57111 | RAB25 | -4.0 | RAB25, member RAS oncogene family |
| 7162 | TPBG | -4.0 | trophoblast glycoprotein |
| 57586 | SYT13 | -3.9 | synaptotagmin XIII |
| 1969 | EPHA2 | -3.9 | EPH receptor A2 |
| 152330 | CNTN4 | -3.9 | contactin 4 |
| 284422 | C19orf77 | -3.9 | chromosome 19 open reading frame 77 |
| 5209 | PFKFB3 | -3.9 | 6-phosphofructo-2-kinase/fructose-2,6-biphosphatase 3 |
| 8531 | YBX3 | -3.9 | Y box binding protein 3 |
| 29909 | GPR171 | -3.9 | G protein-coupled receptor 171 |
| 54825 | CDHR2 | -3.9 | cadherin-related family member 2 |
| 27319 | BHLHE22 | -3.9 | basic helix-loop-helix family, member e22 |
| 11259 | FILIP1L | -3.8 | filamin A interacting protein 1-like |
| 26353 | HSPB8 | -3.8 | heat shock 22kDa protein 8 |
| 79987 | SVEP1 | -3.8 | sushi, von Willebrand factor type A, EGF and pentraxin domain containing 1 |
| 1958 | EGR1 | -3.8 | early growth response 1 |
| 56892 | C8orf4 | -3.8 | chromosome 8 open reading frame 4 |
| 5996 | RGS1 | -3.8 | regulator of G-protein signaling 1 |
| 5069 | PAPPA | -3.8 | pregnancy-associated plasma protein A, pappalysin 1 |
| 4061 | LY6E | -3.8 | lymphocyte antigen 6 complex, locus E |
| 147798 | TMC4 | -3.7 | transmembrane channel-like 4 |
| 390 | RND3 | -3.7 | Rho family GTPase 3 |
| 10231 | RCAN2 | -3.7 | regulator of calcineurin 2 |
| 6913 | TBX15 | -3.7 | T-box 15 |
| 25891 | PAMR1 | -3.7 | peptidase domain containing associated with muscle regeneration 1 |
| 4360 | MRC1 | -3.7 | mannose receptor, C type 1 |
| 2012 | EMP1 | -3.7 | epithelial membrane protein 1 |
| 4494 | MT1F | -3.7 | metallothionein 1F |
| 781 | CACNA2D1 | -3.7 | calcium channel, voltage-dependent, alpha 2/delta subunit 1 |
| 23231 | SEL1L3 | -3.7 | sel-1 suppressor of lin-12-like 3 (C. elegans) |
| 5101 | PCDH9 | -3.7 | protocadherin 9 |
| 23066 | CAND2 | -3.6 | cullin-associated and neddylation-dissociated 2 (putative) |
| 26999 | CYFIP2 | -3.6 | cytoplasmic FMR1 interacting protein 2 |
| 4093 | SMAD9 | -3.6 | SMAD family member 9 |
| 54855 | FAM46C | -3.6 | family with sequence similarity 46, member C |
| 8608 | RDH16 | -3.6 | retinol dehydrogenase 16 (all-trans) |
| 2327 | FMO2 | -3.6 | flavin containing monooxygenase 2 (non-functional) |
| 79884 | MAP9 | -3.6 | microtubule-associated protein 9 |
| 23254 | KAZN | -3.5 | kazrin, periplakin interacting protein |
| 3491 | CYR61 | -3.5 | cysteine-rich, angiogenic inducer, 61 |
| 2743 | GLRB | -3.5 | glycine receptor, beta |
| 64092 | SAMSN1 | -3.5 | SAM domain, SH3 domain and nuclear localization signals 1 |
| 10123 | ARL4C | -3.5 | ADP-ribosylation factor-like 4C |
| 10584 | COLEC10 | -3.5 | collectin sub-family member 10 (C-type lectin) |
| 57161 | PELI2 | -3.5 | pellino E3 ubiquitin protein ligase family member 2 |
| 3003 | GZMK | -3.5 | granzyme K (granzyme 3; tryptase II) |
| 5272 | SERPINB9 | -3.5 | serpin peptidase inhibitor, clade B (ovalbumin), member 9 |
| 117289 | TAGAP | -3.4 | T-cell activation RhoGTPase activating protein |
| 137835 | TMEM71 | -3.4 | transmembrane protein 71 |
| 10797 | MTHFD2 | -3.4 | methylenetetrahydrofolate dehydrogenase (NADP+ dependent) 2, methenyltetrahydrofolate cyclohydrolase |
| 633 | BGN | -3.4 | biglycan |
| 285097 | FLJ38379 | -3.4 | uncharacterized FLJ38379 |
| 1846 | DUSP4 | -3.4 | dual specificity phosphatase 4 |
| 1286 | COL4A4 | -3.4 | collagen, type IV, alpha 4 |
| 912 | CD1D | -3.4 | CD1d molecule |
| 9595 | CYTIP | -3.3 | cytohesin 1 interacting protein |
| 2027 | ENO3 | -3.3 | enolase 3 (beta, muscle) |
| 51339 | DACT1 | -3.3 | dishevelled-binding antagonist of beta-catenin 1 |
| 558 | AXL | -3.3 | AXL receptor tyrosine kinase |
| 10875 | FGL2 | -3.3 | fibrinogen-like 2 |
| 3553 | IL1B | -3.3 | interleukin 1, beta |
| 577 | BAI3 | -3.3 | brain-specific angiogenesis inhibitor 3 |
| 23114 | NFASC | -3.3 | neurofascin |
| 23255 | SOGA2 | -3.3 | SOGA family member 2 |
| 79674 | VEPH1 | -3.3 | ventricular zone expressed PH domain-containing 1 |
| 3428 | IFI16 | -3.2 | interferon, gamma-inducible protein 16 |
| 6279 | S100A8 | -3.2 | S100 calcium binding protein A8 |
| 5577 | PRKAR2B | -3.2 | protein kinase, cAMP-dependent, regulatory, type II, beta |
| 1844 | DUSP2 | -3.2 | dual specificity phosphatase 2 |
| 827 | CAPN6 | -3.2 | calpain 6 |
| 81704 | DOCK8 | -3.2 | dedicator of cytokinesis 8 |
| 1028 | CDKN1C | -3.2 | cyclin-dependent kinase inhibitor 1C (p57, Kip2) |
| 4239 | MFAP4 | -3.2 | microfibrillar-associated protein 4 |
| 1847 | DUSP5 | -3.2 | dual specificity phosphatase 5 |
| 80031 | SEMA6D | -3.2 | sema domain, transmembrane domain (TM), and cytoplasmic domain, (semaphorin) 6D |
| 84620 | ST6GAL2 | -3.2 | ST6 beta-galactosamide alpha-2,6-sialyltranferase 2 |
| 3772 | KCNJ15 | -3.2 | potassium inwardly-rectifying channel, subfamily J, member 15 |
| 23052 | ENDOD1 | -3.2 | endonuclease domain containing 1 |
| 55859 | BEX1 | -3.2 | brain expressed, X-linked 1 |
| 10516 | FBLN5 | -3.2 | fibulin 5 |
| 100628315 | DNM3OS | -3.1 | DNM3 opposite strand/antisense RNA |
| 79804 | HAND2-AS1 | -3.1 | HAND2 antisense RNA 1 (head to head) |
| 83854 | ANGPTL6 | -3.1 | angiopoietin-like 6 |
| 29940 | DSE | -3.1 | dermatan sulfate epimerase |
| 7538 | ZFP36 | -3.1 | ZFP36 ring finger protein |
| 5329 | PLAUR | -3.1 | plasminogen activator, urokinase receptor |
| 4692 | NDN | -3.1 | necdin, melanoma antigen (MAGE) family member |
| 2919 | CXCL1 | -3.1 | chemokine (C-X-C motif) ligand 1 (melanoma growth stimulating activity, alpha) |
| 4493 | MT1E | -3.1 | metallothionein 1E |
| 55195 | C14orf105 | -3.1 | chromosome 14 open reading frame 105 |
| 658 | BMPR1B | -3.1 | bone morphogenetic protein receptor, type IB |
| 9332 | CD163 | -3.1 | CD163 molecule |
| 5592 | PRKG1 | -3.0 | protein kinase, cGMP-dependent, type I |
| 8835 | SOCS2 | -3.0 | suppressor of cytokine signaling 2 |
| 317649 | EIF4E3 | -3.0 | eukaryotic translation initiation factor 4E family member 3 |
| 9945 | GFPT2 | -3.0 | glutamine-fructose-6-phosphate transaminase 2 |
| 3908 | LAMA2 | -3.0 | laminin, alpha 2 |
| 3037 | HAS2 | -3.0 | hyaluronan synthase 2 |
| 54510 | PCDH18 | -3.0 | protocadherin 18 |
| 64078 | SLC28A3 | -3.0 | solute carrier family 28 (concentrative nucleoside transporter), member 3 |
| 3294 | HSD17B2 | -3.0 | hydroxysteroid (17-beta) dehydrogenase 2 |
| 1880 | GPR183 | -3.0 | G protein-coupled receptor 183 |
| 23111 | SPG20 | -3.0 | spastic paraplegia 20 (Troyer syndrome) |
| 114884 | OSBPL10 | -3.0 | oxysterol binding protein-like 10 |
| 84803 | AGPAT9 | -3.0 | 1-acylglycerol-3-phosphate O-acyltransferase 9 |
| 6505 | SLC1A1 | -3.0 | solute carrier family 1 (neuronal/epithelial high affinity glutamate transporter, system Xag), member 1 |
| 6999 | TDO2 | -3.0 | tryptophan 2,3-dioxygenase |
| 2294 | FOXF1 | -3.0 | forkhead box F1 |
| 283897 | C16orf54 | -3.0 | chromosome 16 open reading frame 54 |
| 9719 | ADAMTSL2 | -3.0 | ADAMTS-like 2 |
| 10439 | OLFM1 | -3.0 | olfactomedin 1 |
| 645784 | ANKRD36BP2 | -2.9 | ankyrin repeat domain 36B pseudogene 2 |
| 240 | ALOX5 | -2.9 | arachidonate 5-lipoxygenase |
| 165 | AEBP1 | -2.9 | AE binding protein 1 |
| 2 | A2M | -2.9 | alpha-2-macroglobulin |
| 94122 | SYTL5 | -2.9 | synaptotagmin-like 5 |
| 639 | PRDM1 | -2.9 | PR domain containing 1, with ZNF domain |
| 4837 | NNMT | -2.9 | nicotinamide N-methyltransferase |
| 23136 | EPB41L3 | -2.9 | erythrocyte membrane protein band 4.1-like 3 |
| 2121 | EVC | -2.9 | Ellis van Creveld syndrome |
| 483 | ATP1B3 | -2.9 | ATPase, Na+/K+ transporting, beta 3 polypeptide |
| 192668 | CYS1 | -2.9 | cystin 1 |
| 26960 | NBEA | -2.9 | neurobeachin |
| 2203 | FBP1 | -2.9 | fructose-1,6-bisphosphatase 1 |
| 9805 | SCRN1 | -2.9 | secernin 1 |
| 55186 | SLC25A36 | -2.9 | solute carrier family 25 (pyrimidine nucleotide carrier ), member 36 |
| 2687 | GGT5 | -2.9 | gamma-glutamyltransferase 5 |
| 11093 | ADAMTS13 | -2.9 | ADAM metallopeptidase with thrombospondin type 1 motif, 13 |
| 284207 | METRNL | -2.9 | meteorin, glial cell differentiation regulator-like |
| 10863 | ADAM28 | -2.9 | ADAM metallopeptidase domain 28 |
| 2934 | GSN | -2.9 | gelsolin |
| 2357 | FPR1 | -2.9 | formyl peptide receptor 1 |
| 54331 | GNG2 | -2.9 | guanine nucleotide binding protein (G protein), gamma 2 |
| 131368 | ZPLD1 | -2.9 | zona pellucida-like domain containing 1 |
| 1893 | ECM1 | -2.8 | extracellular matrix protein 1 |
| 445 | ASS1 | -2.8 | argininosuccinate synthase 1 |
| 22915 | MMRN1 | -2.8 | multimerin 1 |
| 5648 | MASP1 | -2.8 | mannan-binding lectin serine peptidase 1 (C4/C2 activating component of Ra-reactive factor) |
| 4675 | NAP1L3 | -2.8 | nucleosome assembly protein 1-like 3 |
| 80760 | ITIH5 | -2.8 | inter-alpha-trypsin inhibitor heavy chain family, member 5 |
| 6402 | SELL | -2.8 | selectin L |
| 2124 | EVI2B | -2.8 | ecotropic viral integration site 2B |
| 2643 | GCH1 | -2.8 | GTP cyclohydrolase 1 |
| 345557 | PLCXD3 | -2.8 | phosphatidylinositol-specific phospholipase C, X domain containing 3 |
| 6542 | SLC7A2 | -2.8 | solute carrier family 7 (cationic amino acid transporter, y+ system), member 2 |
| 2047 | EPHB1 | -2.8 | EPH receptor B1 |
| 2627 | GATA6 | -2.8 | GATA binding protein 6 |
| 64651 | CSRNP1 | -2.8 | cysteine-serine-rich nuclear protein 1 |
| 89797 | NAV2 | -2.8 | neuron navigator 2 |
| 89857 | KLHL6 | -2.8 | kelch-like family member 6 |
| 3702 | ITK | -2.8 | IL2-inducible T-cell kinase |
| 185 | AGTR1 | -2.8 | angiotensin II receptor, type 1 |
| 55843 | ARHGAP15 | -2.8 | Rho GTPase activating protein 15 |
| 100507098 | ADAMTS9-AS2 | -2.8 | ADAMTS9 antisense RNA 2 |
| 4772 | NFATC1 | -2.8 | nuclear factor of activated T-cells, cytoplasmic, calcineurin-dependent 1 |
| 5552 | SRGN | -2.8 | serglycin |
| 57604 | KIAA1456 | -2.8 | KIAA1456 |
| 100128252 | ZNF667-AS1 | -2.7 | ZNF667 antisense RNA 1 (head to head) |
| 5788 | PTPRC | -2.7 | protein tyrosine phosphatase, receptor type, C |
| 4753 | NELL2 | -2.7 | NEL-like 2 (chicken) |
| 8321 | FZD1 | -2.7 | frizzled family receptor 1 |
| 220 | ALDH1A3 | -2.7 | aldehyde dehydrogenase 1 family, member A3 |
| 3480 | IGF1R | -2.7 | insulin-like growth factor 1 receptor |
| 22925 | PLA2R1 | -2.7 | phospholipase A2 receptor 1, 180kDa |
| 85480 | TSLP | -2.7 | thymic stromal lymphopoietin |
| 57088 | PLSCR4 | -2.7 | phospholipid scramblase 4 |
| 256691 | MAMDC2 | -2.7 | MAM domain containing 2 |
| 51175 | TUBE1 | -2.7 | tubulin, epsilon 1 |
| 79895 | ATP8B4 | -2.7 | ATPase, class I, type 8B, member 4 |
| 8612 | PPAP2C | -2.7 | phosphatidic acid phosphatase type 2C |
| 4071 | TM4SF1 | -2.7 | transmembrane 4 L six family member 1 |
| 23764 | MAFF | -2.7 | v-maf avian musculoaponeurotic fibrosarcoma oncogene homolog F |
| 9771 | RAPGEF5 | -2.7 | Rap guanine nucleotide exchange factor (GEF) 5 |
| 5652 | PRSS8 | -2.7 | protease, serine, 8 |
| 55784 | MCTP2 | -2.7 | multiple C2 domains, transmembrane 2 |
| 57452 | GALNT16 | -2.7 | UDP-N-acetyl-alpha-D-galactosamine:polypeptide N-acetylgalactosaminyltransferase 16 |
| 115352 | FCRL3 | -2.7 | Fc receptor-like 3 |
| 5322 | PLA2G5 | -2.7 | phospholipase A2, group V |
| 8382 | NME5 | -2.7 | NME/NM23 family member 5 |
| 8676 | STX11 | -2.6 | syntaxin 11 |
| 1973 | EIF4A1 | -2.6 | eukaryotic translation initiation factor 4A1 |
| 85453 | TSPYL5 | -2.6 | TSPY-like 5 |
| 135112 | NCOA7 | -2.6 | nuclear receptor coactivator 7 |
| 401474 | SAMD12 | -2.6 | sterile alpha motif domain containing 12 |
| 4332 | MNDA | -2.6 | myeloid cell nuclear differentiation antigen |
| 23554 | TSPAN12 | -2.6 | tetraspanin 12 |
| 81617 | CAB39L | -2.6 | calcium binding protein 39-like |
| 133418 | EMB | -2.6 | embigin |
| 144165 | PRICKLE1 | -2.6 | prickle homolog 1 (Drosophila) |
| 79695 | GALNT12 | -2.6 | UDP-N-acetyl-alpha-D-galactosamine:polypeptide N-acetylgalactosaminyltransferase 12 (GalNAc-T12) |
| 441168 | FAM26F | -2.6 | family with sequence similarity 26, member F |
| 80323 | CCDC68 | -2.6 | coiled-coil domain containing 68 |
| 83394 | PITPNM3 | -2.6 | PITPNM family member 3 |
| 7552 | ZNF711 | -2.6 | zinc finger protein 711 |
| 59271 | EVA1C | -2.6 | eva-1 homolog C (C. elegans) |
| 5321 | PLA2G4A | -2.6 | phospholipase A2, group IVA (cytosolic, calcium-dependent) |
| 79098 | C1orf116 | -2.6 | chromosome 1 open reading frame 116 |
| 3976 | LIF | -2.6 | leukemia inhibitory factor |
| 5549 | PRELP | -2.6 | proline/arginine-rich end leucine-rich repeat protein |
| 3399 | ID3 | -2.6 | inhibitor of DNA binding 3, dominant negative helix-loop-helix protein |
| 11117 | EMILIN1 | -2.6 | elastin microfibril interfacer 1 |
| 27115 | PDE7B | -2.6 | phosphodiesterase 7B |
| 6337 | SCNN1A | -2.6 | sodium channel, non-voltage-gated 1 alpha subunit |
| 200634 | KRTCAP3 | -2.5 | keratinocyte associated protein 3 |
| 10659 | CELF2 | -2.5 | CUGBP, Elav-like family member 2 |
| 6462 | SHBG | -2.5 | sex hormone-binding globulin |
| 29957 | SLC25A24 | -2.5 | solute carrier family 25 (mitochondrial carrier; phosphate carrier), member 24 |
| 3489 | IGFBP6 | -2.5 | insulin-like growth factor binding protein 6 |
| 8821 | INPP4B | -2.5 | inositol polyphosphate-4-phosphatase, type II, 105kDa |
| 1160 | CKMT2 | -2.5 | creatine kinase, mitochondrial 2 (sarcomeric) |
| 91663 | MYADM | -2.5 | myeloid-associated differentiation marker |
| 10320 | IKZF1 | -2.5 | IKAROS family zinc finger 1 (Ikaros) |
| 8807 | IL18RAP | -2.5 | interleukin 18 receptor accessory protein |
| 4922 | NTS | -2.5 | neurotensin |
| 57502 | NLGN4X | -2.5 | neuroligin 4, X-linked |
| 944 | TNFSF8 | -2.5 | tumor necrosis factor (ligand) superfamily, member 8 |
| 6422 | SFRP1 | -2.5 | secreted frizzled-related protein 1 |
| 6403 | SELP | -2.5 | selectin P (granule membrane protein 140kDa, antigen CD62) |
| 64231 | MS4A6A | -2.5 | membrane-spanning 4-domains, subfamily A, member 6A |
| 256236 | NAPSB | -2.5 | napsin B aspartic peptidase, pseudogene |
| 6943 | TCF21 | -2.5 | transcription factor 21 |
| 84247 | LDOC1L | -2.5 | leucine zipper, down-regulated in cancer 1-like |
| 29951 | PDZRN4 | -2.5 | PDZ domain containing ring finger 4 |
| 168455 | CCDC71L | -2.5 | coiled-coil domain containing 71-like |
| 9638 | FEZ1 | -2.5 | fasciculation and elongation protein zeta 1 (zygin I) |
| 6546 | SLC8A1 | -2.5 | solute carrier family 8 (sodium/calcium exchanger), member 1 |
| 1839 | HBEGF | -2.5 | heparin-binding EGF-like growth factor |
| 140 | ADORA3 | -2.5 | adenosine A3 receptor |
| 196051 | PPAPDC1A | -2.5 | phosphatidic acid phosphatase type 2 domain containing 1A |
| 10319 | LAMC3 | -2.5 | laminin, gamma 3 |
| 100128537 | C1orf132 | -2.5 | chromosome 1 open reading frame 132 |
| 1592 | CYP26A1 | -2.5 | cytochrome P450, family 26, subfamily A, polypeptide 1 |
| 100506098 | LOC100506098 | -2.5 | uncharacterized LOC100506098 |
| 57134 | MAN1C1 | -2.5 | mannosidase, alpha, class 1C, member 1 |
| 51660 | MPC1 | -2.5 | mitochondrial pyruvate carrier 1 |
| 22822 | PHLDA1 | -2.5 | pleckstrin homology-like domain, family A, member 1 |
| 84617 | TUBB6 | -2.5 | tubulin, beta 6 class V |
| 3606 | IL18 | -2.4 | interleukin 18 (interferon-gamma-inducing factor) |
| 10409 | BASP1 | -2.4 | brain abundant, membrane attached signal protein 1 |
| 91607 | SLFN11 | -2.4 | schlafen family member 11 |
| 5294 | PIK3CG | -2.4 | phosphatidylinositol-4,5-bisphosphate 3-kinase, catalytic subunit gamma |
| 56271 | BEX4 | -2.4 | brain expressed, X-linked 4 |
| 4065 | LY75 | -2.4 | lymphocyte antigen 75 |
| 9173 | IL1RL1 | -2.4 | interleukin 1 receptor-like 1 |
| 114548 | NLRP3 | -2.4 | NLR family, pyrin domain containing 3 |
| 729230 | CCR2 | -2.4 | chemokine (C-C motif) receptor 2 |
| 54558 | SPATA6 | -2.4 | spermatogenesis associated 6 |
| 23641 | LDOC1 | -2.4 | leucine zipper, down-regulated in cancer 1 |
| 219972 | MPEG1 | -2.4 | macrophage expressed 1 |
| 80704 | SLC19A3 | -2.4 | solute carrier family 19 (thiamine transporter), member 3 |
| 79627 | OGFRL1 | -2.4 | opioid growth factor receptor-like 1 |
| 2850 | GPR27 | -2.4 | G protein-coupled receptor 27 |
| 4684 | NCAM1 | -2.4 | neural cell adhesion molecule 1 |
| 55821 | ALLC | -2.4 | allantoicase |
| 79722 | ANKRD55 | -2.4 | ankyrin repeat domain 55 |
| 29931 | LINC00312 | -2.4 | long intergenic non-protein coding RNA 312 |
| 55228 | PNMAL1 | -2.4 | paraneoplastic Ma antigen family-like 1 |
| 57713 | SFMBT2 | -2.4 | Scm-like with four mbt domains 2 |
| 5613 | PRKX | -2.4 | protein kinase, X-linked |
| 81606 | LBH | -2.4 | limb bud and heart development |
| 63982 | ANO3 | -2.4 | anoctamin 3 |
| 6352 | CCL5 | -2.4 | chemokine (C-C motif) ligand 5 |
| 1390 | CREM | -2.4 | cAMP responsive element modulator |
| 5446 | PON3 | -2.4 | paraoxonase 3 |
| 56898 | BDH2 | -2.4 | 3-hydroxybutyrate dehydrogenase, type 2 |
| 9262 | STK17B | -2.4 | serine/threonine kinase 17b |
| 84766 | EFCAB4B | -2.4 | EF-hand calcium binding domain 4B |
| 5799 | PTPRN2 | -2.4 | protein tyrosine phosphatase, receptor type, N polypeptide 2 |
| 3937 | LCP2 | -2.4 | lymphocyte cytosolic protein 2 (SH2 domain containing leukocyte protein of 76kDa) |
| 8793 | TNFRSF10D | -2.4 | tumor necrosis factor receptor superfamily, member 10d, decoy with truncated death domain |
| 6503 | SLA | -2.4 | Src-like-adaptor |
| 284454 | LOC284454 | -2.3 | uncharacterized LOC284454 |
| 10586 | MAB21L2 | -2.3 | mab-21-like 2 (C. elegans) |
| 10125 | RASGRP1 | -2.3 | RAS guanyl releasing protein 1 (calcium and DAG-regulated) |
| 7456 | WIPF1 | -2.3 | WAS/WASL interacting protein family, member 1 |
| 2526 | FUT4 | -2.3 | fucosyltransferase 4 (alpha (1,3) fucosyltransferase, myeloid-specific) |
| 5915 | RARB | -2.3 | retinoic acid receptor, beta |
| 23151 | GRAMD4 | -2.3 | GRAM domain containing 4 |
| 4213 | MEIS3P1 | -2.3 | Meis homeobox 3 pseudogene 1 |
| 3059 | HCLS1 | -2.3 | hematopoietic cell-specific Lyn substrate 1 |
| 9172 | MYOM2 | -2.3 | myomesin 2 |
| 84913 | ATOH8 | -2.3 | atonal homolog 8 (Drosophila) |
| 7357 | UGCG | -2.3 | UDP-glucose ceramide glucosyltransferase |
| 8809 | IL18R1 | -2.3 | interleukin 18 receptor 1 |
| 864 | RUNX3 | -2.3 | runt-related transcription factor 3 |
| 55576 | STAB2 | -2.3 | stabilin 2 |
| 25819 | CCRN4L | -2.3 | CCR4 carbon catabolite repression 4-like (S. cerevisiae) |
| 284013 | VMO1 | -2.3 | vitelline membrane outer layer 1 homolog (chicken) |
| 7700 | ZNF141 | -2.3 | zinc finger protein 141 |
| 26227 | PHGDH | -2.3 | phosphoglycerate dehydrogenase |
| 4063 | LY9 | -2.3 | lymphocyte antigen 9 |
| 284219 | LOC284219 | -2.3 | uncharacterized LOC284219 |
| 5621 | PRNP | -2.3 | prion protein |
| 26034 | IPCEF1 | -2.3 | interaction protein for cytohesin exchange factors 1 |
| 4327 | MMP19 | -2.3 | matrix metallopeptidase 19 |
| 1236 | CCR7 | -2.3 | chemokine (C-C motif) receptor 7 |
| 143689 | PIWIL4 | -2.3 | piwi-like RNA-mediated gene silencing 4 |
| 54847 | SIDT1 | -2.3 | SID1 transmembrane family, member 1 |
| 5214 | PFKP | -2.3 | phosphofructokinase, platelet |
| 144193 | AMDHD1 | -2.3 | amidohydrolase domain containing 1 |
| 8425 | LTBP4 | -2.3 | latent transforming growth factor beta binding protein 4 |
| 307 | ANXA4 | -2.3 | annexin A4 |
| 10135 | NAMPT | -2.3 | nicotinamide phosphoribosyltransferase |
| 2114 | ETS2 | -2.3 | v-ets avian erythroblastosis virus E26 oncogene homolog 2 |
| 23345 | SYNE1 | -2.3 | spectrin repeat containing, nuclear envelope 1 |
| 9823 | ARMCX2 | -2.3 | armadillo repeat containing, X-linked 2 |
| 64919 | BCL11B | -2.3 | B-cell CLL/lymphoma 11B (zinc finger protein) |
| 10673 | TNFSF13B | -2.3 | tumor necrosis factor (ligand) superfamily, member 13b |
| 79730 | NSUN7 | -2.3 | NOP2/Sun domain family, member 7 |
| 4921 | DDR2 | -2.3 | discoidin domain receptor tyrosine kinase 2 |
| 2308 | FOXO1 | -2.3 | forkhead box O1 |
| 54762 | GRAMD1C | -2.3 | GRAM domain containing 1C |
| 5271 | SERPINB8 | -2.2 | serpin peptidase inhibitor, clade B (ovalbumin), member 8 |
| 55106 | SLFN12 | -2.2 | schlafen family member 12 |
| 9737 | GPRASP1 | -2.2 | G protein-coupled receptor associated sorting protein 1 |
| 440515 | ZNF506 | -2.2 | zinc finger protein 506 |
| 640 | BLK | -2.2 | B lymphoid tyrosine kinase |
| 118429 | ANTXR2 | -2.2 | anthrax toxin receptor 2 |
| 3918 | LAMC2 | -2.2 | laminin, gamma 2 |
| 7071 | KLF10 | -2.2 | Kruppel-like factor 10 |
| 6366 | CCL21 | -2.2 | chemokine (C-C motif) ligand 21 |
| 9590 | AKAP12 | -2.2 | A kinase (PRKA) anchor protein 12 |
| 9834 | KIAA0125 | -2.2 | KIAA0125 |
| 22856 | CHSY1 | -2.2 | chondroitin sulfate synthase 1 |
| 2690 | GHR | -2.2 | growth hormone receptor |
| 57556 | SEMA6A | -2.2 | sema domain, transmembrane domain (TM), and cytoplasmic domain, (semaphorin) 6A |
| 7430 | EZR | -2.2 | ezrin |
| 963 | CD53 | -2.2 | CD53 molecule |
| 203522 | DDX26B | -2.2 | DEAD/H (Asp-Glu-Ala-Asp/His) box polypeptide 26B |
| 57554 | LRRC7 | -2.2 | leucine rich repeat containing 7 |
| 3726 | JUNB | -2.2 | jun B proto-oncogene |
| 10253 | SPRY2 | -2.2 | sprouty homolog 2 (Drosophila) |
| 128346 | C1orf162 | -2.2 | chromosome 1 open reading frame 162 |
| 116984 | ARAP2 | -2.2 | ArfGAP with RhoGAP domain, ankyrin repeat and PH domain 2 |
| 84159 | ARID5B | -2.2 | AT rich interactive domain 5B (MRF1-like) |
| 10507 | SEMA4D | -2.2 | sema domain, immunoglobulin domain (Ig), transmembrane domain (TM) and short cytoplasmic domain, (semaphorin) 4D |
| 1621 | DBH | -2.2 | dopamine beta-hydroxylase (dopamine beta-monooxygenase) |
| 64581 | CLEC7A | -2.2 | C-type lectin domain family 7, member A |
| 55647 | RAB20 | -2.2 | RAB20, member RAS oncogene family |
| 128218 | TMEM125 | -2.2 | transmembrane protein 125 |
| 26136 | TES | -2.2 | testis derived transcript (3 LIM domains) |
| 2260 | FGFR1 | -2.2 | fibroblast growth factor receptor 1 |
| 57484 | RNF150 | -2.2 | ring finger protein 150 |
| 146857 | SLFN13 | -2.2 | schlafen family member 13 |
| 728 | C5AR1 | -2.2 | complement component 5a receptor 1 |
| 925 | CD8A | -2.2 | CD8a molecule |
| 64332 | NFKBIZ | -2.2 | nuclear factor of kappa light polypeptide gene enhancer in B-cells inhibitor, zeta |
| 440 | ASNS | -2.1 | asparagine synthetase (glutamine-hydrolyzing) |
| 113828 | FAM83F | -2.1 | family with sequence similarity 83, member F |
| 9586 | CREB5 | -2.1 | cAMP responsive element binding protein 5 |
| 22998 | LIMCH1 | -2.1 | LIM and calponin homology domains 1 |
| 9077 | DIRAS3 | -2.1 | DIRAS family, GTP-binding RAS-like 3 |
| 7145 | TNS1 | -2.1 | tensin 1 |
| 4291 | MLF1 | -2.1 | myeloid leukemia factor 1 |
| 219699 | UNC5B | -2.1 | unc-5 homolog B (C. elegans) |
| 56849 | TCEAL7 | -2.1 | transcription elongation factor A (SII)-like 7 |
| 55619 | DOCK10 | -2.1 | dedicator of cytokinesis 10 |
| 3398 | ID2 | -2.1 | inhibitor of DNA binding 2, dominant negative helix-loop-helix protein |
| 9639 | ARHGEF10 | -2.1 | Rho guanine nucleotide exchange factor (GEF) 10 |
| 51166 | AADAT | -2.1 | aminoadipate aminotransferase |
| 6775 | STAT4 | -2.1 | signal transducer and activator of transcription 4 |
| 2244 | FGB | -2.1 | fibrinogen beta chain |
| 85460 | ZNF518B | -2.1 | zinc finger protein 518B |
| 344148 | NCKAP5 | -2.1 | NCK-associated protein 5 |
| 100169890 | PEG3-AS1 | -2.1 | PEG3 antisense RNA 1 |
| 151963 | MB21D2 | -2.1 | Mab-21 domain containing 2 |
| 79974 | CPED1 | -2.1 | cadherin-like and PC-esterase domain containing 1 |
| 6351 | CCL4 | -2.1 | chemokine (C-C motif) ligand 4 |
| 6915 | TBXA2R | -2.1 | thromboxane A2 receptor |
| 64805 | P2RY12 | -2.1 | purinergic receptor P2Y, G-protein coupled, 12 |
| 9214 | FAIM3 | -2.1 | Fas apoptotic inhibitory molecule 3 |
| 1296 | COL8A2 | -2.1 | collagen, type VIII, alpha 2 |
| 4068 | SH2D1A | -2.1 | SH2 domain containing 1A |
| 8477 | GPR65 | -2.1 | G protein-coupled receptor 65 |
| 2331 | FMOD | -2.1 | fibromodulin |
| 5745 | PTH1R | -2.1 | parathyroid hormone 1 receptor |
| 5019 | OXCT1 | -2.1 | 3-oxoacid CoA transferase 1 |
| 3959 | LGALS3BP | -2.1 | lectin, galactoside-binding, soluble, 3 binding protein |
| 64407 | RGS18 | -2.1 | regulator of G-protein signaling 18 |
| 25801 | GCA | -2.1 | grancalcin, EF-hand calcium binding protein |
| 8140 | SLC7A5 | -2.1 | solute carrier family 7 (amino acid transporter light chain, L system), member 5 |
| 9734 | HDAC9 | -2.1 | histone deacetylase 9 |
| 3036 | HAS1 | -2.1 | hyaluronan synthase 1 |
| 28514 | DLL1 | -2.1 | delta-like 1 (Drosophila) |
| 9770 | RASSF2 | -2.1 | Ras association (RalGDS/AF-6) domain family member 2 |
| 5366 | PMAIP1 | -2.1 | phorbol-12-myristate-13-acetate-induced protein 1 |
| 1488 | CTBP2 | -2.1 | C-terminal binding protein 2 |
| 79720 | VPS37B | -2.1 | vacuolar protein sorting 37 homolog B (S. cerevisiae) |
| 50852 | TRAT1 | -2.1 | T cell receptor associated transmembrane adaptor 1 |
| 5579 | PRKCB | -2.1 | protein kinase C, beta |
| 79161 | TMEM243 | -2.1 | transmembrane protein 243, mitochondrial |
| 286676 | ILDR1 | -2.1 | immunoglobulin-like domain containing receptor 1 |
| 54674 | LRRN3 | -2.1 | leucine rich repeat neuronal 3 |
| 64218 | SEMA4A | -2.1 | sema domain, immunoglobulin domain (Ig), transmembrane domain (TM) and short cytoplasmic domain, (semaphorin) 4A |
| 5336 | PLCG2 | -2.1 | phospholipase C, gamma 2 (phosphatidylinositol-specific) |
| 256380 | SCML4 | -2.1 | sex comb on midleg-like 4 (Drosophila) |
| 6692 | SPINT1 | -2.1 | serine peptidase inhibitor, Kunitz type 1 |
| 5359 | PLSCR1 | -2.1 | phospholipid scramblase 1 |
| 53832 | IL20RA | -2.0 | interleukin 20 receptor, alpha |
| 28639 | TRBC1 | -2.0 | T cell receptor beta constant 1 |
| 652995 | UCA1 | -2.0 | urothelial cancer associated 1 (non-protein coding) |
| 9308 | CD83 | -2.0 | CD83 molecule |
| 57688 | ZSWIM6 | -2.0 | zinc finger, SWIM-type containing 6 |
| 7113 | TMPRSS2 | -2.0 | transmembrane protease, serine 2 |
| 9706 | ULK2 | -2.0 | unc-51 like autophagy activating kinase 2 |
| 1848 | DUSP6 | -2.0 | dual specificity phosphatase 6 |
| 5764 | PTN | -2.0 | pleiotrophin |
| 624 | BDKRB2 | -2.0 | bradykinin receptor B2 |
| 91316 | GUSBP11 | -2.0 | glucuronidase, beta pseudogene 11 |
| 5783 | PTPN13 | -2.0 | protein tyrosine phosphatase, non-receptor type 13 (APO-1/CD95 (Fas)-associated phosphatase) |
| 3394 | IRF8 | -2.0 | interferon regulatory factor 8 |
| 58499 | ZNF462 | -2.0 | zinc finger protein 462 |
| 1902 | LPAR1 | -2.0 | lysophosphatidic acid receptor 1 |
| 7275 | TUB | -2.0 | tubby bipartite transcription factor |
| 57476 | GRAMD1B | -2.0 | GRAM domain containing 1B |
| 8609 | KLF7 | -2.0 | Kruppel-like factor 7 (ubiquitous) |
| 57447 | NDRG2 | -2.0 | NDRG family member 2 |
| 100289090 | LOC100289090 | -2.0 | uncharacterized LOC100289090 |
| 2064 | ERBB2 | -2.0 | v-erb-b2 avian erythroblastic leukemia viral oncogene homolog 2 |
| 6875 | TAF4B | -2.0 | TAF4b RNA polymerase II, TATA box binding protein (TBP)-associated factor, 105kDa |
| 2205 | FCER1A | -2.0 | Fc fragment of IgE, high affinity I, receptor for; alpha polypeptide |
| 23294 | ANKS1A | -2.0 | ankyrin repeat and sterile alpha motif domain containing 1A |
| 51237 | MZB1 | -2.0 | marginal zone B and B1 cell-specific protein |
| 113802 | HENMT1 | -2.0 | HEN1 methyltransferase homolog 1 (Arabidopsis) |
| 596 | BCL2 | -2.0 | B-cell CLL/lymphoma 2 |
| 3725 | JUN | -2.0 | jun proto-oncogene |
| 64333 | ARHGAP9 | -2.0 | Rho GTPase activating protein 9 |
| 3111 | HLA-DOA | -2.0 | major histocompatibility complex, class II, DO alpha |
| 222223 | KIAA1324L | -2.0 | KIAA1324-like |
| 89870 | TRIM15 | -2.0 | tripartite motif containing 15 |
| 100505573 | LINC00984 | -2.0 | long intergenic non-protein coding RNA 984 |
| 9056 | SLC7A7 | -2.0 | solute carrier family 7 (amino acid transporter light chain, y+L system), member 7 |
| 58189 | WFDC1 | -2.0 | WAP four-disulfide core domain 1 |
| 23514 | SPIDR | -2.0 | scaffolding protein involved in DNA repair |
| 8631 | SKAP1 | -2.0 | src kinase associated phosphoprotein 1 |
| 6850 | SYK | -2.0 | spleen tyrosine kinase |
| 6344 | SCTR | -2.0 | secretin receptor |
| 80183 | KIAA0226L | -2.0 | KIAA0226-like |
| 26115 | TANC2 | -1.9 | tetratricopeptide repeat, ankyrin repeat and coiled-coil containing 2 |
| 814 | CAMK4 | -1.9 | calcium/calmodulin-dependent protein kinase IV |
| 29950 | SERTAD1 | -1.9 | SERTA domain containing 1 |
| 83593 | RASSF5 | -1.9 | Ras association (RalGDS/AF-6) domain family member 5 |
| 2949 | GSTM5 | -1.9 | glutathione S-transferase mu 5 |
| 51477 | ISYNA1 | -1.9 | inositol-3-phosphate synthase 1 |
| 962 | CD48 | -1.9 | CD48 molecule |
| 2730 | GCLM | -1.9 | glutamate-cysteine ligase, modifier subunit |
| 976 | CD97 | -1.9 | CD97 molecule |
| 5144 | PDE4D | -1.9 | phosphodiesterase 4D, cAMP-specific |
| 7975 | MAFK | -1.9 | v-maf avian musculoaponeurotic fibrosarcoma oncogene homolog K |
| 340024 | SLC6A19 | -1.9 | solute carrier family 6 (neutral amino acid transporter), member 19 |
| 7433 | VIPR1 | -1.9 | vasoactive intestinal peptide receptor 1 |
| 57210 | SLC45A4 | -1.9 | solute carrier family 45, member 4 |
| 3002 | GZMB | -1.9 | granzyme B (granzyme 2, cytotoxic T-lymphocyte-associated serine esterase 1) |
| 3601 | IL15RA | -1.9 | interleukin 15 receptor, alpha |
| 85450 | ITPRIP | -1.9 | inositol 1,4,5-trisphosphate receptor interacting protein |
| 196383 | RILPL2 | -1.9 | Rab interacting lysosomal protein-like 2 |
| 150696 | PROM2 | -1.9 | prominin 2 |
| 9450 | LY86 | -1.9 | lymphocyte antigen 86 |
| 285440 | CYP4V2 | -1.9 | cytochrome P450, family 4, subfamily V, polypeptide 2 |
| 79778 | MICALL2 | -1.9 | MICAL-like 2 |
| 11145 | PLA2G16 | -1.9 | phospholipase A2, group XVI |
| 10451 | VAV3 | -1.9 | vav 3 guanine nucleotide exchange factor |
| 26051 | PPP1R16B | -1.9 | protein phosphatase 1, regulatory subunit 16B |
| 255231 | MCOLN2 | -1.9 | mucolipin 2 |
| 270 | AMPD1 | -1.9 | adenosine monophosphate deaminase 1 |
| 51090 | PLLP | -1.9 | plasmolipin |
| 6886 | TAL1 | -1.9 | T-cell acute lymphocytic leukemia 1 |
| 100505746 | ITGB2-AS1 | -1.9 | ITGB2 antisense RNA 1 |
| 22807 | IKZF2 | -1.9 | IKAROS family zinc finger 2 (Helios) |
| 8553 | BHLHE40 | -1.9 | basic helix-loop-helix family, member e40 |
| 10974 | ADIRF | -1.9 | adipogenesis regulatory factor |
| 168667 | BMPER | -1.9 | BMP binding endothelial regulator |
| 54407 | SLC38A2 | -1.9 | solute carrier family 38, member 2 |
| 375033 | PEAR1 | -1.9 | platelet endothelial aggregation receptor 1 |
| 9473 | THEMIS2 | -1.9 | thymocyte selection associated family member 2 |
| 9938 | ARHGAP25 | -1.9 | Rho GTPase activating protein 25 |
| 2625 | GATA3 | -1.9 | GATA binding protein 3 |
| 7133 | TNFRSF1B | -1.9 | tumor necrosis factor receptor superfamily, member 1B |
| 50615 | IL21R | -1.9 | interleukin 21 receptor |
| 5606 | MAP2K3 | -1.9 | mitogen-activated protein kinase kinase 3 |
| 2533 | FYB | -1.9 | FYN binding protein |
| 2841 | GPR18 | -1.9 | G protein-coupled receptor 18 |
| 8613 | PPAP2B | -1.8 | phosphatidic acid phosphatase type 2B |
| 25976 | TIPARP | -1.8 | TCDD-inducible poly(ADP-ribose) polymerase |
| 30811 | HUNK | -1.8 | hormonally up-regulated Neu-associated kinase |
| 3718 | JAK3 | -1.8 | Janus kinase 3 |
| 8110 | DPF3 | -1.8 | D4, zinc and double PHD fingers, family 3 |
| 3579 | CXCR2 | -1.8 | chemokine (C-X-C motif) receptor 2 |
| 1410 | CRYAB | -1.8 | crystallin, alpha B |
| 5026 | P2RX5 | -1.8 | purinergic receptor P2X, ligand-gated ion channel, 5 |
| 201477 | DLGAP1-AS3 | -1.8 | DLGAP1 antisense RNA 3 |
| 408 | ARRB1 | -1.8 | arrestin, beta 1 |
| 149628 | PYHIN1 | -1.8 | pyrin and HIN domain family, member 1 |
| 3932 | LCK | -1.8 | lymphocyte-specific protein tyrosine kinase |
| 5293 | PIK3CD | -1.8 | phosphatidylinositol-4,5-bisphosphate 3-kinase, catalytic subunit delta |
| 796 | CALCA | -1.8 | calcitonin-related polypeptide alpha |
| 285195 | SLC9A9 | -1.8 | solute carrier family 9, subfamily A (NHE9, cation proton antiporter 9), member 9 |
| 84959 | UBASH3B | -1.8 | ubiquitin associated and SH3 domain containing B |
| 1612 | DAPK1 | -1.8 | death-associated protein kinase 1 |
| 84671 | ZNF347 | -1.8 | zinc finger protein 347 |
| 8516 | ITGA8 | -1.8 | integrin, alpha 8 |
| 952 | CD38 | -1.8 | CD38 molecule |
| 56253 | CRTAM | -1.8 | cytotoxic and regulatory T cell molecule |
| 8483 | CILP | -1.8 | cartilage intermediate layer protein, nucleotide pyrophosphohydrolase |
| 7414 | VCL | -1.8 | vinculin |
| 11314 | CD300A | -1.8 | CD300a molecule |
| 9689 | BZW1 | -1.8 | basic leucine zipper and W2 domains 1 |
| 147138 | TMC8 | -1.8 | transmembrane channel-like 8 |
| 54149 | C21orf91 | -1.8 | chromosome 21 open reading frame 91 |
| 80342 | TRAF3IP3 | -1.8 | TRAF3 interacting protein 3 |
| 8645 | KCNK5 | -1.8 | potassium channel, subfamily K, member 5 |
| 92086 | GGTLC1 | -1.8 | gamma-glutamyltransferase light chain 1 |
| 171425 | CLYBL | -1.8 | citrate lyase beta like |
| 23533 | PIK3R5 | -1.8 | phosphoinositide-3-kinase, regulatory subunit 5 |
| 6523 | SLC5A1 | -1.8 | solute carrier family 5 (sodium/glucose cotransporter), member 1 |
| 340061 | TMEM173 | -1.8 | transmembrane protein 173 |
| 59339 | PLEKHA2 | -1.8 | pleckstrin homology domain containing, family A (phosphoinositide binding specific) member 2 |
| 94120 | SYTL3 | -1.8 | synaptotagmin-like 3 |
| 57689 | LRRC4C | -1.8 | leucine rich repeat containing 4C |
| 24147 | FJX1 | -1.8 | four jointed box 1 (Drosophila) |
| 80714 | PBX4 | -1.8 | pre-B-cell leukemia homeobox 4 |
| 153020 | RASGEF1B | -1.8 | RasGEF domain family, member 1B |
| 89790 | SIGLEC10 | -1.8 | sialic acid binding Ig-like lectin 10 |
| 284406 | ZFP82 | -1.8 | ZFP82 zinc finger protein |
| 89846 | FGD3 | -1.8 | FYVE, RhoGEF and PH domain containing 3 |
| 4908 | NTF3 | -1.8 | neurotrophin 3 |
| 63934 | ZNF667 | -1.8 | zinc finger protein 667 |
| 55016 | MARCH1 | -1.8 | membrane-associated ring finger (C3HC4) 1, E3 ubiquitin protein ligase |
| 10288 | LILRB2 | -1.8 | leukocyte immunoglobulin-like receptor, subfamily B (with TM and ITIM domains), member 2 |
| 9902 | MRC2 | -1.8 | mannose receptor, C type 2 |
| 9535 | GMFG | -1.7 | glia maturation factor, gamma |
| 285513 | GPRIN3 | -1.7 | GPRIN family member 3 |
| 7378 | UPP1 | -1.7 | uridine phosphorylase 1 |
| 80117 | ARL14 | -1.7 | ADP-ribosylation factor-like 14 |
| 124220 | ZG16B | -1.7 | zymogen granule protein 16B |
| 23639 | LRRC6 | -1.7 | leucine rich repeat containing 6 |
| 54900 | LAX1 | -1.7 | lymphocyte transmembrane adaptor 1 |
| 9121 | SLC16A5 | -1.7 | solute carrier family 16 (monocarboxylate transporter), member 5 |
| 8530 | CST7 | -1.7 | cystatin F (leukocystatin) |
| 9459 | ARHGEF6 | -1.7 | Rac/Cdc42 guanine nucleotide exchange factor (GEF) 6 |
| 863 | CBFA2T3 | -1.7 | core-binding factor, runt domain, alpha subunit 2; translocated to, 3 |
| 143425 | SYT9 | -1.7 | synaptotagmin IX |
| 27163 | NAAA | -1.7 | N-acylethanolamine acid amidase |
| 654 | BMP6 | -1.7 | bone morphogenetic protein 6 |
| 140883 | ZNF280B | -1.7 | zinc finger protein 280B |
| 55024 | BANK1 | -1.7 | B-cell scaffold protein with ankyrin repeats 1 |
| 911 | CD1C | -1.7 | CD1c molecule |
| 23682 | RAB38 | -1.7 | RAB38, member RAS oncogene family |
| 9447 | AIM2 | -1.7 | absent in melanoma 2 |
| 55466 | DNAJA4 | -1.7 | DnaJ (Hsp40) homolog, subfamily A, member 4 |
| 124460 | SNX20 | -1.7 | sorting nexin 20 |
| 80149 | ZC3H12A | -1.7 | zinc finger CCCH-type containing 12A |
| 54511 | HMGCLL1 | -1.7 | 3-hydroxymethyl-3-methylglutaryl-CoA lyase-like 1 |
| 9469 | CHST3 | -1.7 | carbohydrate (chondroitin 6) sulfotransferase 3 |
| 80228 | ORAI2 | -1.7 | ORAI calcium release-activated calcium modulator 2 |
| 2950 | GSTP1 | -1.7 | glutathione S-transferase pi 1 |
| 9806 | SPOCK2 | -1.7 | sparc/osteonectin, cwcv and kazal-like domains proteoglycan (testican) 2 |
| 54206 | ERRFI1 | -1.7 | ERBB receptor feedback inhibitor 1 |
| 51389 | RWDD1 | -1.7 | RWD domain containing 1 |
| 79875 | THSD4 | -1.7 | thrombospondin, type I, domain containing 4 |
| 3431 | SP110 | -1.7 | SP110 nuclear body protein |
| 54869 | EPS8L1 | -1.7 | EPS8-like 1 |
| 79977 | GRHL2 | -1.7 | grainyhead-like 2 (Drosophila) |
| 144501 | KRT80 | -1.7 | keratin 80 |
| 692205 | SNORD89 | -1.7 | small nucleolar RNA, C/D box 89 |
| 10150 | MBNL2 | -1.7 | muscleblind-like splicing regulator 2 |
| 3560 | IL2RB | -1.7 | interleukin 2 receptor, beta |
| 26191 | PTPN22 | -1.7 | protein tyrosine phosphatase, non-receptor type 22 (lymphoid) |
| 131873 | COL6A6 | -1.7 | collagen, type VI, alpha 6 |
| 1946 | EFNA5 | -1.7 | ephrin-A5 |
| 119587 | CPXM2 | -1.7 | carboxypeptidase X (M14 family), member 2 |
| 5880 | RAC2 | -1.7 | ras-related C3 botulinum toxin substrate 2 (rho family, small GTP binding protein Rac2) |
| 6928 | HNF1B | -1.7 | HNF1 homeobox B |
| 9170 | LPAR2 | -1.7 | lysophosphatidic acid receptor 2 |
| 11234 | HPS5 | -1.7 | Hermansky-Pudlak syndrome 5 |
| 4033 | LRMP | -1.7 | lymphoid-restricted membrane protein |
| 55713 | ZNF334 | -1.7 | zinc finger protein 334 |
| 10888 | GPR83 | -1.7 | G protein-coupled receptor 83 |
| 286133 | SCARA5 | -1.7 | scavenger receptor class A, member 5 (putative) |
| 4857 | NOVA1 | -1.7 | neuro-oncological ventral antigen 1 |
| 138046 | RALYL | -1.7 | RALY RNA binding protein-like |
| 1263 | PLK3 | -1.7 | polo-like kinase 3 |
| 26118 | WSB1 | -1.7 | WD repeat and SOCS box containing 1 |
| 313 | AOAH | -1.7 | acyloxyacyl hydrolase (neutrophil) |
| 402778 | IFITM10 | -1.7 | interferon induced transmembrane protein 10 |
| 9200 | PTPLA | -1.7 | protein tyrosine phosphatase-like (proline instead of catalytic arginine), member A |
| 6404 | SELPLG | -1.7 | selectin P ligand |
| 2069 | EREG | -1.7 | epiregulin |
| 4118 | MAL | -1.7 | mal, T-cell differentiation protein |
| 55041 | PLEKHB2 | -1.7 | pleckstrin homology domain containing, family B (evectins) member 2 |
| 55064 | SPATA6L | -1.7 | spermatogenesis associated 6-like |
| 896 | CCND3 | -1.6 | cyclin D3 |
| 152926 | PPM1K | -1.6 | protein phosphatase, Mg2+/Mn2+ dependent, 1K |
| 241 | ALOX5AP | -1.6 | arachidonate 5-lipoxygenase-activating protein |
| 25817 | FAM19A5 | -1.6 | family with sequence similarity 19 (chemokine (C-C motif)-like), member A5 |
| 340152 | ZC3H12D | -1.6 | zinc finger CCCH-type containing 12D |
| 10957 | PNRC1 | -1.6 | proline-rich nuclear receptor coactivator 1 |
| 10053 | AP1M2 | -1.6 | adaptor-related protein complex 1, mu 2 subunit |
| 971 | CD72 | -1.6 | CD72 molecule |
| 26228 | STAP1 | -1.6 | signal transducing adaptor family member 1 |
| 100506779 | BZRAP1-AS1 | -1.6 | BZRAP1 antisense RNA 1 |
| 113612 | CYP2U1 | -1.6 | cytochrome P450, family 2, subfamily U, polypeptide 1 |
| 112616 | CMTM7 | -1.6 | CKLF-like MARVEL transmembrane domain containing 7 |
| 127435 | PODN | -1.6 | podocan |
| 93035 | PKHD1L1 | -1.6 | polycystic kidney and hepatic disease 1 (autosomal recessive)-like 1 |
| 9435 | CHST2 | -1.6 | carbohydrate (N-acetylglucosamine-6-O) sulfotransferase 2 |
| 134466 | ZNF300P1 | -1.6 | zinc finger protein 300 pseudogene 1 |
| 79152 | FA2H | -1.6 | fatty acid 2-hydroxylase |
| 144406 | WDR66 | -1.6 | WD repeat domain 66 |
| 11322 | TMC6 | -1.6 | transmembrane channel-like 6 |
| 8320 | EOMES | -1.6 | eomesodermin |
| 5858 | PZP | -1.6 | pregnancy-zone protein |
| 9218 | VAPA | -1.6 | VAMP (vesicle-associated membrane protein)-associated protein A, 33kDa |
| 64084 | CLSTN2 | -1.6 | calsyntenin 2 |
| 3707 | ITPKB | -1.6 | inositol-trisphosphate 3-kinase B |
| 9182 | RASSF9 | -1.6 | Ras association (RalGDS/AF-6) domain family (N-terminal) member 9 |
| 27334 | P2RY10 | -1.6 | purinergic receptor P2Y, G-protein coupled, 10 |
| 79037 | PVRIG | -1.6 | poliovirus receptor related immunoglobulin domain containing |
| 54733 | SLC35F2 | -1.6 | solute carrier family 35, member F2 |
| 9052 | GPRC5A | -1.6 | G protein-coupled receptor, family C, group 5, member A |
| 9744 | ACAP1 | -1.6 | ArfGAP with coiled-coil, ankyrin repeat and PH domains 1 |
| 93010 | B3GNT7 | -1.6 | UDP-GlcNAc:betaGal beta-1,3-N-acetylglucosaminyltransferase 7 |
| 30817 | EMR2 | -1.6 | egf-like module containing, mucin-like, hormone receptor-like 2 |
| 440823 | MIAT | -1.6 | myocardial infarction associated transcript (non-protein coding) |
| 80709 | AKNA | -1.6 | AT-hook transcription factor |
| 7043 | TGFB3 | -1.6 | transforming growth factor, beta 3 |
| 11072 | DUSP14 | -1.6 | dual specificity phosphatase 14 |
| 6425 | SFRP5 | -1.6 | secreted frizzled-related protein 5 |
| 100506870 | LOC100506870 | -1.6 | uncharacterized LOC100506870 |
| 10620 | ARID3B | -1.6 | AT rich interactive domain 3B (BRIGHT-like) |
| 387923 | SERP2 | -1.6 | stress-associated endoplasmic reticulum protein family member 2 |
| 8467 | SMARCA5 | -1.6 | SWI/SNF related, matrix associated, actin dependent regulator of chromatin, subfamily a, member 5 |
| 202309 | GAPT | -1.6 | GRB2-binding adaptor protein, transmembrane |
| 1318 | SLC31A2 | -1.6 | solute carrier family 31 (copper transporter), member 2 |
| 113402 | SFT2D1 | -1.6 | SFT2 domain containing 1 |
| 83416 | FCRL5 | -1.6 | Fc receptor-like 5 |
| 926 | CD8B | -1.6 | CD8b molecule |
| 10492 | SYNCRIP | -1.6 | synaptotagmin binding, cytoplasmic RNA interacting protein |
| 23645 | PPP1R15A | -1.6 | protein phosphatase 1, regulatory subunit 15A |
| 51011 | FAHD2A | -1.6 | fumarylacetoacetate hydrolase domain containing 2A |
| 4541 | ND6 | -1.6 | NADH dehydrogenase, subunit 6 (complex I) |
| 9188 | DDX21 | -1.6 | DEAD (Asp-Glu-Ala-Asp) box helicase 21 |
| 126917 | IFFO2 | -1.6 | intermediate filament family orphan 2 |
| 57402 | S100A14 | -1.6 | S100 calcium binding protein A14 |
| 11213 | IRAK3 | -1.6 | interleukin-1 receptor-associated kinase 3 |
| 51696 | HECA | -1.5 | headcase homolog (Drosophila) |
| 2624 | GATA2 | -1.5 | GATA binding protein 2 |
| 11262 | SP140 | -1.5 | SP140 nuclear body protein |
| 4689 | NCF4 | -1.5 | neutrophil cytosolic factor 4, 40kDa |
| 678 | ZFP36L2 | -1.5 | ZFP36 ring finger protein-like 2 |
| 128611 | ZNF831 | -1.5 | zinc finger protein 831 |
| 11027 | LILRA2 | -1.5 | leukocyte immunoglobulin-like receptor, subfamily A (with TM domain), member 2 |
| 439949 | PRKCQ-AS1 | -1.5 | PRKCQ antisense RNA 1 |
| 401074 | LINC00960 | -1.5 | long intergenic non-protein coding RNA 960 |
| 9053 | MAP7 | -1.5 | microtubule-associated protein 7 |
| 399 | RHOH | -1.5 | ras homolog family member H |
| 132884 | EVC2 | -1.5 | Ellis van Creveld syndrome 2 |
| 22898 | DENND3 | -1.5 | DENN/MADD domain containing 3 |
| 199 | AIF1 | -1.5 | allograft inflammatory factor 1 |
| 942 | CD86 | -1.5 | CD86 molecule |
| 56000 | NXF3 | -1.5 | nuclear RNA export factor 3 |
| 50802 | IGK | -1.5 | immunoglobulin kappa locus |
| 5966 | REL | -1.5 | v-rel avian reticuloendotheliosis viral oncogene homolog |
| 5604 | MAP2K1 | -1.5 | mitogen-activated protein kinase kinase 1 |
| 50861 | STMN3 | -1.5 | stathmin-like 3 |
| 10870 | HCST | -1.5 | hematopoietic cell signal transducer |
| 9043 | SPAG9 | -1.5 | sperm associated antigen 9 |
| 5742 | PTGS1 | -1.5 | prostaglandin-endoperoxide synthase 1 (prostaglandin G/H synthase and cyclooxygenase) |
| 2921 | CXCL3 | -1.5 | chemokine (C-X-C motif) ligand 3 |
| 54436 | SH3TC1 | -1.5 | SH3 domain and tetratricopeptide repeats 1 |
| 11040 | PIM2 | -1.5 | pim-2 oncogene |
| 26511 | CHIC2 | -1.5 | cysteine-rich hydrophobic domain 2 |
| 158434 | LOC158434 | -1.5 | uncharacterized LOC158434 |
| 7409 | VAV1 | -1.5 | vav 1 guanine nucleotide exchange factor |
| 619208 | FAM229B | -1.5 | family with sequence similarity 229, member B |
| 23261 | CAMTA1 | -1.5 | calmodulin binding transcription activator 1 |
| 130576 | LYPD6B | -1.5 | LY6/PLAUR domain containing 6B |
| 1741 | DLG3 | -1.5 | discs, large homolog 3 (Drosophila) |
| 917 | CD3G | -1.5 | CD3g molecule, gamma (CD3-TCR complex) |
| 1606 | DGKA | -1.5 | diacylglycerol kinase, alpha 80kDa |
| 100132074 | FOXO6 | -1.5 | forkhead box O6 |
| 64115 | C10orf54 | -1.5 | chromosome 10 open reading frame 54 |
| 374403 | TBC1D10C | -1.5 | TBC1 domain family, member 10C |
| 920 | CD4 | -1.5 | CD4 molecule |
| 51411 | BIN2 | -1.5 | bridging integrator 2 |
| 919 | CD247 | -1.5 | CD247 molecule |
| 220164 | DOK6 | -1.4 | docking protein 6 |
| 7148 | TNXB | -1.4 | tenascin XB |
| 1997 | ELF1 | -1.4 | E74-like factor 1 (ets domain transcription factor) |
| 84958 | SYTL1 | -1.4 | synaptotagmin-like 1 |
| 29101 | SSU72 | -1.4 | SSU72 RNA polymerase II CTD phosphatase homolog (S. cerevisiae) |
| 346007 | EYS | -1.4 | eyes shut homolog (Drosophila) |
| 653 | BMP5 | -1.4 | bone morphogenetic protein 5 |
| 2081 | ERN1 | -1.4 | endoplasmic reticulum to nucleus signaling 1 |
| 57110 | HRASLS | -1.4 | HRAS-like suppressor |
| 22806 | IKZF3 | -1.4 | IKAROS family zinc finger 3 (Aiolos) |
| 6622 | SNCA | -1.4 | synuclein, alpha (non A4 component of amyloid precursor) |
| 23012 | STK38L | -1.4 | serine/threonine kinase 38 like |
| 8115 | TCL1A | -1.4 | T-cell leukemia/lymphoma 1A |
| 7033 | TFF3 | -1.4 | trefoil factor 3 (intestinal) |
| 11184 | MAP4K1 | -1.4 | mitogen-activated protein kinase kinase kinase kinase 1 |
| 6774 | STAT3 | -1.4 | signal transducer and activator of transcription 3 (acute-phase response factor) |
| 5368 | PNOC | -1.4 | prepronociceptin |
| 6283 | S100A12 | -1.4 | S100 calcium binding protein A12 |
| 913 | CD1E | -1.4 | CD1e molecule |
| 8715 | NOL4 | -1.4 | nucleolar protein 4 |
| 6103 | RPGR | -1.3 | retinitis pigmentosa GTPase regulator |
| 387751 | GVINP1 | -1.3 | GTPase, very large interferon inducible pseudogene 1 |
| 65997 | RASL11B | -1.3 | RAS-like, family 11, member B |
| 93664 | CADPS2 | 1.3 | Ca++-dependent secretion activator 2 |
| 8087 | FXR1 | 1.4 | fragile X mental retardation, autosomal homolog 1 |
| 79095 | C9orf16 | 1.4 | chromosome 9 open reading frame 16 |
| 375513 | GUSBP4 | 1.4 | glucuronidase, beta pseudogene 4 |
| 113235 | SLC46A1 | 1.4 | solute carrier family 46 (folate transporter), member 1 |
| 10299 | MARCH6 | 1.4 | membrane-associated ring finger (C3HC4) 6, E3 ubiquitin protein ligase |
| 10155 | TRIM28 | 1.4 | tripartite motif containing 28 |
| 55755 | CDK5RAP2 | 1.4 | CDK5 regulatory subunit associated protein 2 |
| 158158 | RASEF | 1.5 | RAS and EF-hand domain containing |
| 649159 | LINC00273 | 1.5 | long intergenic non-protein coding RNA 273 |
| 28996 | HIPK2 | 1.5 | homeodomain interacting protein kinase 2 |
| 92610 | TIFA | 1.5 | TRAF-interacting protein with forkhead-associated domain |
| 5439 | POLR2J | 1.5 | polymerase (RNA) II (DNA directed) polypeptide J, 13.3kDa |
| 440026 | TMEM41B | 1.5 | transmembrane protein 41B |
| 2175 | FANCA | 1.5 | Fanconi anemia, complementation group A |
| 10283 | CWC27 | 1.5 | CWC27 spliceosome-associated protein homolog (S. cerevisiae) |
| 5605 | MAP2K2 | 1.5 | mitogen-activated protein kinase kinase 2 |
| 2222 | FDFT1 | 1.5 | farnesyl-diphosphate farnesyltransferase 1 |
| 56894 | AGPAT3 | 1.5 | 1-acylglycerol-3-phosphate O-acyltransferase 3 |
| 3187 | HNRNPH1 | 1.5 | heterogeneous nuclear ribonucleoprotein H1 (H) |
| 79075 | DSCC1 | 1.5 | DNA replication and sister chromatid cohesion 1 |
| 9530 | BAG4 | 1.5 | BCL2-associated athanogene 4 |
| 55957 | LIN37 | 1.5 | lin-37 homolog (C. elegans) |
| 10280 | SIGMAR1 | 1.5 | sigma non-opioid intracellular receptor 1 |
| 254048 | UBN2 | 1.5 | ubinuclein 2 |
| 55749 | CCAR1 | 1.5 | cell division cycle and apoptosis regulator 1 |
| 27 | ABL2 | 1.5 | c-abl oncogene 2, non-receptor tyrosine kinase |
| 9673 | SLC25A44 | 1.5 | solute carrier family 25, member 44 |
| 51495 | PTPLAD1 | 1.5 | protein tyrosine phosphatase-like A domain containing 1 |
| 25917 | THUMPD3 | 1.5 | THUMP domain containing 3 |
| 79685 | SAP30L | 1.5 | SAP30-like |
| 6881 | TAF10 | 1.6 | TAF10 RNA polymerase II, TATA box binding protein (TBP)-associated factor, 30kDa |
| 10587 | TXNRD2 | 1.6 | thioredoxin reductase 2 |
| 55662 | HIF1AN | 1.6 | hypoxia inducible factor 1, alpha subunit inhibitor |
| 6599 | SMARCC1 | 1.6 | SWI/SNF related, matrix associated, actin dependent regulator of chromatin, subfamily c, member 1 |
| 100289635 | ZNF605 | 1.6 | zinc finger protein 605 |
| 2264 | FGFR4 | 1.6 | fibroblast growth factor receptor 4 |
| 8907 | AP1M1 | 1.6 | adaptor-related protein complex 1, mu 1 subunit |
| 90522 | YIF1B | 1.6 | Yip1 interacting factor homolog B (S. cerevisiae) |
| 25778 | DSTYK | 1.6 | dual serine/threonine and tyrosine protein kinase |
| 139231 | FAM199X | 1.6 | family with sequence similarity 199, X-linked |
| 283871 | PGP | 1.6 | phosphoglycolate phosphatase |
| 55605 | KIF21A | 1.6 | kinesin family member 21A |
| 8943 | AP3D1 | 1.6 | adaptor-related protein complex 3, delta 1 subunit |
| 51478 | HSD17B7 | 1.6 | hydroxysteroid (17-beta) dehydrogenase 7 |
| 84105 | PCBD2 | 1.6 | pterin-4 alpha-carbinolamine dehydratase/dimerization cofactor of hepatocyte nuclear factor 1 alpha (TCF1) 2 |
| 8815 | BANF1 | 1.6 | barrier to autointegration factor 1 |
| 2494 | NR5A2 | 1.6 | nuclear receptor subfamily 5, group A, member 2 |
| 3268 | AGFG2 | 1.6 | ArfGAP with FG repeats 2 |
| 336 | APOA2 | 1.6 | apolipoprotein A-II |
| 84079 | ANKRD27 | 1.6 | ankyrin repeat domain 27 (VPS9 domain) |
| 54856 | GON4L | 1.6 | gon-4-like (C. elegans) |
| 4047 | LSS | 1.6 | lanosterol synthase (2,3-oxidosqualene-lanosterol cyclase) |
| 90806 | ANGEL2 | 1.6 | angel homolog 2 (Drosophila) |
| 10238 | DCAF7 | 1.6 | DDB1 and CUL4 associated factor 7 |
| 126626 | GABPB2 | 1.6 | GA binding protein transcription factor, beta subunit 2 |
| 22836 | RHOBTB3 | 1.6 | Rho-related BTB domain containing 3 |
| 5591 | PRKDC | 1.7 | protein kinase, DNA-activated, catalytic polypeptide |
| 80270 | HSD3B7 | 1.7 | hydroxy-delta-5-steroid dehydrogenase, 3 beta- and steroid delta-isomerase 7 |
| 4090 | SMAD5 | 1.7 | SMAD family member 5 |
| 9580 | SOX13 | 1.7 | SRY (sex determining region Y)-box 13 |
| 8805 | TRIM24 | 1.7 | tripartite motif containing 24 |
| 23126 | POGZ | 1.7 | pogo transposable element with ZNF domain |
| 401261 | FLJ38717 | 1.7 | FLJ38717 protein |
| 9448 | MAP4K4 | 1.7 | mitogen-activated protein kinase kinase kinase kinase 4 |
| 25836 | NIPBL | 1.7 | Nipped-B homolog (Drosophila) |
| 23367 | LARP1 | 1.7 | La ribonucleoprotein domain family, member 1 |
| 4548 | MTR | 1.7 | 5-methyltetrahydrofolate-homocysteine methyltransferase |
| 11264 | PXMP4 | 1.7 | peroxisomal membrane protein 4, 24kDa |
| 2770 | GNAI1 | 1.7 | guanine nucleotide binding protein (G protein), alpha inhibiting activity polypeptide 1 |
| 84820 | POLR2J4 | 1.7 | polymerase (RNA) II (DNA directed) polypeptide J4, pseudogene |
| 4338 | MOCS2 | 1.7 | molybdenum cofactor synthesis 2 |
| 51133 | KCTD3 | 1.7 | potassium channel tetramerization domain containing 3 |
| 54935 | DUSP23 | 1.7 | dual specificity phosphatase 23 |
| 92689 | FAM114A1 | 1.7 | family with sequence similarity 114, member A1 |
| 51663 | ZFR | 1.7 | zinc finger RNA binding protein |
| 81627 | TRMT1L | 1.7 | tRNA methyltransferase 1 homolog (S. cerevisiae)-like |
| 284018 | C17orf58 | 1.7 | chromosome 17 open reading frame 58 |
| 4192 | MDK | 1.7 | midkine (neurite growth-promoting factor 2) |
| 54939 | COMMD4 | 1.7 | COMM domain containing 4 |
| 51710 | ZNF44 | 1.7 | zinc finger protein 44 |
| 5380 | PMS2L2 | 1.7 | postmeiotic segregation increased 2-like 2 pseudogene |
| 163183 | SYNE4 | 1.7 | spectrin repeat containing, nuclear envelope family member 4 |
| 10533 | ATG7 | 1.7 | autophagy related 7 |
| 196294 | IMMP1L | 1.7 | IMP1 inner mitochondrial membrane peptidase-like (S. cerevisiae) |
| 286077 | FAM83H | 1.7 | family with sequence similarity 83, member H |
| 150275 | CCDC117 | 1.7 | coiled-coil domain containing 117 |
| 153562 | MARVELD2 | 1.7 | MARVEL domain containing 2 |
| 55031 | USP47 | 1.7 | ubiquitin specific peptidase 47 |
| 2058 | EPRS | 1.7 | glutamyl-prolyl-tRNA synthetase |
| 55900 | ZNF302 | 1.7 | zinc finger protein 302 |
| 56886 | UGGT1 | 1.7 | UDP-glucose glycoprotein glucosyltransferase 1 |
| 26268 | FBXO9 | 1.7 | F-box protein 9 |
| 254013 | METTL20 | 1.7 | methyltransferase like 20 |
| 64746 | ACBD3 | 1.7 | acyl-CoA binding domain containing 3 |
| 11260 | XPOT | 1.7 | exportin, tRNA |
| 5442 | POLRMT | 1.7 | polymerase (RNA) mitochondrial (DNA directed) |
| 29956 | CERS2 | 1.7 | ceramide synthase 2 |
| 83660 | TLN2 | 1.8 | talin 2 |
| 55585 | UBE2Q1 | 1.8 | ubiquitin-conjugating enzyme E2Q family member 1 |
| 57473 | ZNF512B | 1.8 | zinc finger protein 512B |
| 57645 | POGK | 1.8 | pogo transposable element with KRAB domain |
| 6197 | RPS6KA3 | 1.8 | ribosomal protein S6 kinase, 90kDa, polypeptide 3 |
| 56647 | BCCIP | 1.8 | BRCA2 and CDKN1A interacting protein |
| 1943 | EFNA2 | 1.8 | ephrin-A2 |
| 284926 | LOC284926 | 1.8 | uncharacterized LOC284926 |
| 3014 | H2AFX | 1.8 | H2A histone family, member X |
| 100272217 | LOC100272217 | 1.8 | uncharacterized LOC100272217 |
| 28981 | IFT81 | 1.8 | intraflagellar transport 81 homolog (Chlamydomonas) |
| 5383 | PMS2P5 | 1.8 | postmeiotic segregation increased 2 pseudogene 5 |
| 54962 | TIPIN | 1.8 | TIMELESS interacting protein |
| 65125 | WNK1 | 1.8 | WNK lysine deficient protein kinase 1 |
| 7175 | TPR | 1.8 | translocated promoter region, nuclear basket protein |
| 7088 | TLE1 | 1.8 | transducin-like enhancer of split 1 (E(sp1) homolog, Drosophila) |
| 23246 | BOP1 | 1.8 | block of proliferation 1 |
| 54908 | SPDL1 | 1.8 | spindle apparatus coiled-coil protein 1 |
| 23528 | ZNF281 | 1.8 | zinc finger protein 281 |
| 9891 | NUAK1 | 1.8 | NUAK family, SNF1-like kinase, 1 |
| 84318 | CCDC77 | 1.8 | coiled-coil domain containing 77 |
| 3295 | HSD17B4 | 1.8 | hydroxysteroid (17-beta) dehydrogenase 4 |
| 642946 | FLVCR1-AS1 | 1.8 | FLVCR1 antisense RNA 1 (head to head) |
| 1660 | DHX9 | 1.8 | DEAH (Asp-Glu-Ala-His) box helicase 9 |
| 55844 | PPP2R2D | 1.8 | protein phosphatase 2, regulatory subunit B, delta |
| 28956 | LAMTOR2 | 1.8 | late endosomal/lysosomal adaptor, MAPK and MTOR activator 2 |
| 890 | CCNA2 | 1.8 | cyclin A2 |
| 25909 | AHCTF1 | 1.9 | AT hook containing transcription factor 1 |
| 51205 | ACP6 | 1.9 | acid phosphatase 6, lysophosphatidic |
| 1979 | EIF4EBP2 | 1.9 | eukaryotic translation initiation factor 4E binding protein 2 |
| 7283 | TUBG1 | 1.9 | tubulin, gamma 1 |
| 139322 | APOOL | 1.9 | apolipoprotein O-like |
| 3157 | HMGCS1 | 1.9 | 3-hydroxy-3-methylglutaryl-CoA synthase 1 (soluble) |
| 7203 | CCT3 | 1.9 | chaperonin containing TCP1, subunit 3 (gamma) |
| 9532 | BAG2 | 1.9 | BCL2-associated athanogene 2 |
| 5313 | PKLR | 1.9 | pyruvate kinase, liver and RBC |
| 10970 | CKAP4 | 1.9 | cytoskeleton-associated protein 4 |
| 9831 | ZNF623 | 1.9 | zinc finger protein 623 |
| 153769 | SH3RF2 | 1.9 | SH3 domain containing ring finger 2 |
| 554203 | JPX | 1.9 | JPX transcript, XIST activator (non-protein coding) |
| 100381270 | ZBED6 | 1.9 | zinc finger, BED-type containing 6 |
| 284669 | LOC284669 | 1.9 | uncharacterized LOC284669 |
| 5546 | PRCC | 1.9 | papillary renal cell carcinoma (translocation-associated) |
| 54675 | CRLS1 | 1.9 | cardiolipin synthase 1 |
| 9203 | ZMYM3 | 1.9 | zinc finger, MYM-type 3 |
| 22796 | COG2 | 1.9 | component of oligomeric golgi complex 2 |
| 55592 | GOLGA2P5 | 1.9 | golgin A2 pseudogene 5 |
| 8914 | TIMELESS | 1.9 | timeless circadian clock |
| 100289274 | DNAJC3-AS1 | 1.9 | DNAJC3 antisense RNA 1 (head to head) |
| 3570 | IL6R | 2.0 | interleukin 6 receptor |
| 2342 | FNTB | 2.0 | farnesyltransferase, CAAX box, beta |
| 9453 | GGPS1 | 2.0 | geranylgeranyl diphosphate synthase 1 |
| 84134 | TOMM40L | 2.0 | translocase of outer mitochondrial membrane 40 homolog (yeast)-like |
| 11198 | SUPT16H | 2.0 | suppressor of Ty 16 homolog (S. cerevisiae) |
| 9456 | HOMER1 | 2.0 | homer homolog 1 (Drosophila) |
| 55151 | TMEM38B | 2.0 | transmembrane protein 38B |
| 25953 | PNKD | 2.0 | paroxysmal nonkinesigenic dyskinesia |
| 9319 | TRIP13 | 2.0 | thyroid hormone receptor interactor 13 |
| 9582 | APOBEC3B | 2.0 | apolipoprotein B mRNA editing enzyme, catalytic polypeptide-like 3B |
| 11065 | UBE2C | 2.0 | ubiquitin-conjugating enzyme E2C |
| 2237 | FEN1 | 2.0 | flap structure-specific endonuclease 1 |
| 55532 | SLC30A10 | 2.0 | solute carrier family 30, member 10 |
| 2065 | ERBB3 | 2.0 | v-erb-b2 avian erythroblastic leukemia viral oncogene homolog 3 |
| 10134 | BCAP31 | 2.0 | B-cell receptor-associated protein 31 |
| 6573 | SLC19A1 | 2.0 | solute carrier family 19 (folate transporter), member 1 |
| 55313 | CPPED1 | 2.0 | calcineurin-like phosphoesterase domain containing 1 |
| 80010 | RMI1 | 2.0 | RecQ mediated genome instability 1 |
| 10198 | MPHOSPH9 | 2.0 | M-phase phosphoprotein 9 |
| 134553 | C5orf24 | 2.0 | chromosome 5 open reading frame 24 |
| 83692 | CD99L2 | 2.1 | CD99 molecule-like 2 |
| 27252 | KLHL20 | 2.1 | kelch-like family member 20 |
| 9761 | MLEC | 2.1 | malectin |
| 3664 | IRF6 | 2.1 | interferon regulatory factor 6 |
| 200765 | TIGD1 | 2.1 | tigger transposable element derived 1 |
| 84988 | PPP1R16A | 2.1 | protein phosphatase 1, regulatory subunit 16A |
| 5565 | PRKAB2 | 2.1 | protein kinase, AMP-activated, beta 2 non-catalytic subunit |
| 58497 | PRUNE | 2.1 | prune exopolyphosphatase |
| 360023 | ZBTB41 | 2.1 | zinc finger and BTB domain containing 41 |
| 9331 | B4GALT6 | 2.1 | UDP-Gal:betaGlcNAc beta 1,4- galactosyltransferase, polypeptide 6 |
| 163590 | TOR1AIP2 | 2.1 | torsin A interacting protein 2 |
| 79726 | WDR59 | 2.1 | WD repeat domain 59 |
| 5618 | PRLR | 2.1 | prolactin receptor |
| 6502 | SKP2 | 2.1 | S-phase kinase-associated protein 2, E3 ubiquitin protein ligase |
| 730102 | LOC730102 | 2.1 | quinone oxidoreductase-like protein 2 pseudogene |
| 7292 | TNFSF4 | 2.2 | tumor necrosis factor (ligand) superfamily, member 4 |
| 84327 | ZBED3 | 2.2 | zinc finger, BED-type containing 3 |
| 55204 | GOLPH3L | 2.2 | golgi phosphoprotein 3-like |
| 387103 | CENPW | 2.2 | centromere protein W |
| 100129917 | LOC100129917 | 2.2 | uncharacterized LOC100129917 |
| 202020 | TAPT1-AS1 | 2.2 | TAPT1 antisense RNA 1 (head to head) |
| 10535 | RNASEH2A | 2.2 | ribonuclease H2, subunit A |
| 100507316 | LOC100507316 | 2.3 | uncharacterized LOC100507316 |
| 55609 | ZNF280C | 2.3 | zinc finger protein 280C |
| 100507495 | SDCBP2-AS1 | 2.3 | SDCBP2 antisense RNA 1 |
| 100134822 | LOC100134822 | 2.3 | uncharacterized LOC100134822 |
| 127933 | UHMK1 | 2.3 | U2AF homology motif (UHM) kinase 1 |
| 51203 | NUSAP1 | 2.3 | nucleolar and spindle associated protein 1 |
| 9768 | KIAA0101 | 2.3 | KIAA0101 |
| 55796 | MBNL3 | 2.3 | muscleblind-like splicing regulator 3 |
| 10244 | RABEPK | 2.3 | Rab9 effector protein with kelch motifs |
| 2146 | EZH2 | 2.3 | enhancer of zeste homolog 2 (Drosophila) |
| 6334 | SCN8A | 2.3 | sodium channel, voltage gated, type VIII, alpha subunit |
| 7064 | THOP1 | 2.3 | thimet oligopeptidase 1 |
| 1030 | CDKN2B | 2.3 | cyclin-dependent kinase inhibitor 2B (p15, inhibits CDK4) |
| 28982 | FLVCR1 | 2.3 | feline leukemia virus subgroup C cellular receptor 1 |
| 29901 | SAC3D1 | 2.3 | SAC3 domain containing 1 |
| 79682 | MLF1IP | 2.4 | MLF1 interacting protein |
| 80317 | ZKSCAN3 | 2.4 | zinc finger with KRAB and SCAN domains 3 |
| 2305 | FOXM1 | 2.4 | forkhead box M1 |
| 100506328 | LOC100506328 | 2.4 | uncharacterized LOC100506328 |
| 642477 | FLJ39632 | 2.4 | uncharacterized LOC642477 |
| 2630 | GBAP1 | 2.4 | glucosidase, beta, acid pseudogene 1 |
| 1029 | CDKN2A | 2.5 | cyclin-dependent kinase inhibitor 2A |
| 9231 | DLG5 | 2.5 | discs, large homolog 5 (Drosophila) |
| 80303 | EFHD1 | 2.5 | EF-hand domain family, member D1 |
| 10472 | ZBTB18 | 2.5 | zinc finger and BTB domain containing 18 |
| 388722 | C1orf53 | 2.5 | chromosome 1 open reading frame 53 |
| 5174 | PDZK1 | 2.5 | PDZ domain containing 1 |
| 55732 | C1orf112 | 2.6 | chromosome 1 open reading frame 112 |
| 63979 | FIGNL1 | 2.6 | fidgetin-like 1 |
| 1031 | CDKN2C | 2.6 | cyclin-dependent kinase inhibitor 2C (p18, inhibits CDK4) |
| 400793 | C1orf226 | 2.6 | chromosome 1 open reading frame 226 |
| 134548 | SOWAHA | 2.6 | sosondowah ankyrin repeat domain family member A |
| 148738 | HFE2 | 2.6 | hemochromatosis type 2 (juvenile) |
| 91057 | CCDC34 | 2.6 | coiled-coil domain containing 34 |
| 23491 | CES3 | 2.6 | carboxylesterase 3 |
| 23314 | SATB2 | 2.7 | SATB homeobox 2 |
| 83461 | CDCA3 | 2.7 | cell division cycle associated 3 |
| 100506392 | LOC100506392 | 2.7 | uncharacterized LOC100506392 |
| 9837 | GINS1 | 2.7 | GINS complex subunit 1 (Psf1 homolog) |
| 348158 | ACSM2B | 2.8 | acyl-CoA synthetase medium-chain family member 2B |
| 6905 | TBCE | 2.8 | tubulin folding cofactor E |
| 9540 | TP53I3 | 2.9 | tumor protein p53 inducible protein 3 |
| 64151 | NCAPG | 2.9 | non-SMC condensin I complex, subunit G |
| 701 | BUB1B | 3.0 | BUB1 mitotic checkpoint serine/threonine kinase B |
| 284618 | RUSC1-AS1 | 3.1 | RUSC1 antisense RNA 1 |
| 221662 | RBM24 | 3.2 | RNA binding motif protein 24 |
| 10403 | NDC80 | 3.2 | NDC80 kinetochore complex component |
| 9133 | CCNB2 | 3.3 | cyclin B2 |
| 83540 | NUF2 | 3.4 | NUF2, NDC80 kinetochore complex component |
| 9055 | PRC1 | 3.4 | protein regulator of cytokinesis 1 |
| 112609 | MRAP2 | 3.4 | melanocortin 2 receptor accessory protein 2 |
| 1033 | CDKN3 | 3.5 | cyclin-dependent kinase inhibitor 3 |
| 24137 | KIF4A | 3.5 | kinesin family member 4A |
| 10112 | KIF20A | 3.5 | kinesin family member 20A |
| 983 | CDK1 | 3.6 | cyclin-dependent kinase 1 |
| 9833 | MELK | 3.6 | maternal embryonic leucine zipper kinase |
| 29127 | RACGAP1 | 3.7 | Rac GTPase activating protein 1 |
| 144455 | E2F7 | 3.8 | E2F transcription factor 7 |
| 4751 | NEK2 | 3.8 | NIMA-related kinase 2 |
| 8357 | HIST1H3H | 3.9 | histone cluster 1, H3h |
| 55872 | PBK | 4.0 | PDZ binding kinase |
| 891 | CCNB1 | 4.0 | cyclin B1 |
| 8353 | HIST1H3E | 4.1 | histone cluster 1, H3e |
| 1063 | CENPF | 4.2 | centromere protein F, 350/400kDa |
| 10486 | CAP2 | 4.3 | CAP, adenylate cyclase-associated protein, 2 (yeast) |
| 81494 | CFHR5 | 4.7 | complement factor H-related 5 |
| 9928 | KIF14 | 4.8 | kinesin family member 14 |
| 57512 | GPR158 | 4.8 | G protein-coupled receptor 158 |
| 23676 | SMPX | 5.2 | small muscle protein, X-linked |
| 54443 | ANLN | 5.5 | anillin, actin binding protein |
| 3161 | HMMR | 5.6 | hyaluronan-mediated motility receptor (RHAMM) |
| 7153 | TOP2A | 5.7 | topoisomerase (DNA) II alpha 170kDa |
| 81610 | FAM83D | 5.9 | family with sequence similarity 83, member D |
| 3209 | HOXA13 | 6.8 | homeobox A13 |
| 259266 | ASPM | 6.8 | asp (abnormal spindle) homolog, microcephaly associated (Drosophila) |
| 643911 | CRNDE | 7.7 | colorectal neoplasia differentially expressed (non-protein coding) |
| 84675 | TRIM55 | 11.7 | tripartite motif containing 55 |
| 2719 | GPC3 | 12.7 | glypican 3 |
| 6690 | SPINK1 | 19.3 | serine peptidase inhibitor, Kazal type 1 |
